# Supplementary material for: Deconstructing the geography of human impacts on species’ natural distribution
Source: Nat Commun. 2024 Oct 14;15:8852. doi: 10.1038/s41467-024-52993-0 (PMC11473693; doi:10.1038/s41467-024-52993-0)
Supplement: Supplementary file 1 — Supplementary Information [file 41467_2024_52993_MOESM1_ESM.pdf]

Deconstructing the geography of human impacts on species' natural distribution

Supplementary Figures

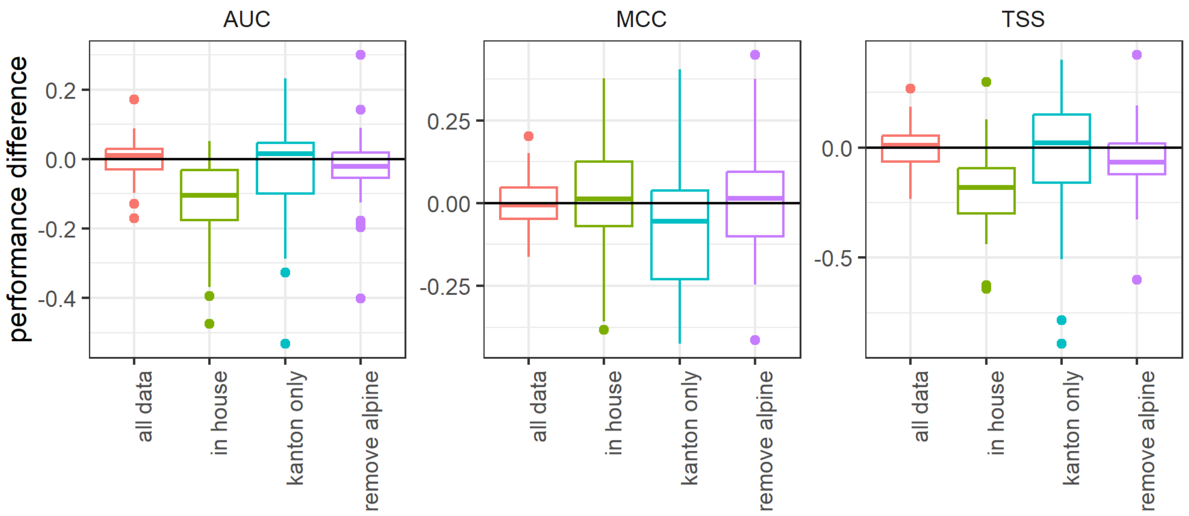

Figure S1. Difference in model performance depending on subsets of the complete compiled dataset. Each panel refers to a different performance metric as (a) area under receiver operator curve (AUC), (b) Matthews correlation coefficient (MCC) and (c) True Skill Statistic (TSS). We assessed the following subsets i) only "in-house" surveys based on scientific research teams and standardized national monitoring data, ii) Canton of Bern monitoring data, iii) all data but removing up-stream alpine catchments that are generally disconnected, cold and fast flowing rivers. We used analysis of variance to test for differences in the mean performance compared with models using all the data. We designed these subsets to check whether performance was increased by including additional cantonal data sources. The mean of inhouse performance for AUC and for TSS was significantly reduced compared to models including all the data ($p<0.001$). All other comparisons between subsets of the data and the full dataset were not significant. These results indicate that high model performance was not driven by different environments of the upstream alpine catchments. Secondly, these results also indicate that the model performance was driven largely by the high quantity of data provided by cantonal monitoring sources compared to in house monitoring. Note that the model evaluations are performed internally on the subset data sources (i-iii) rather than across all data sources. Note also that boxplots contain underlying 5-fold variation in blocked cross validations as well as between species variation in model performance. Boxplots indicate the median (solid line), inter-quartile range (hinges) and 1.5*IQR (whiskers).

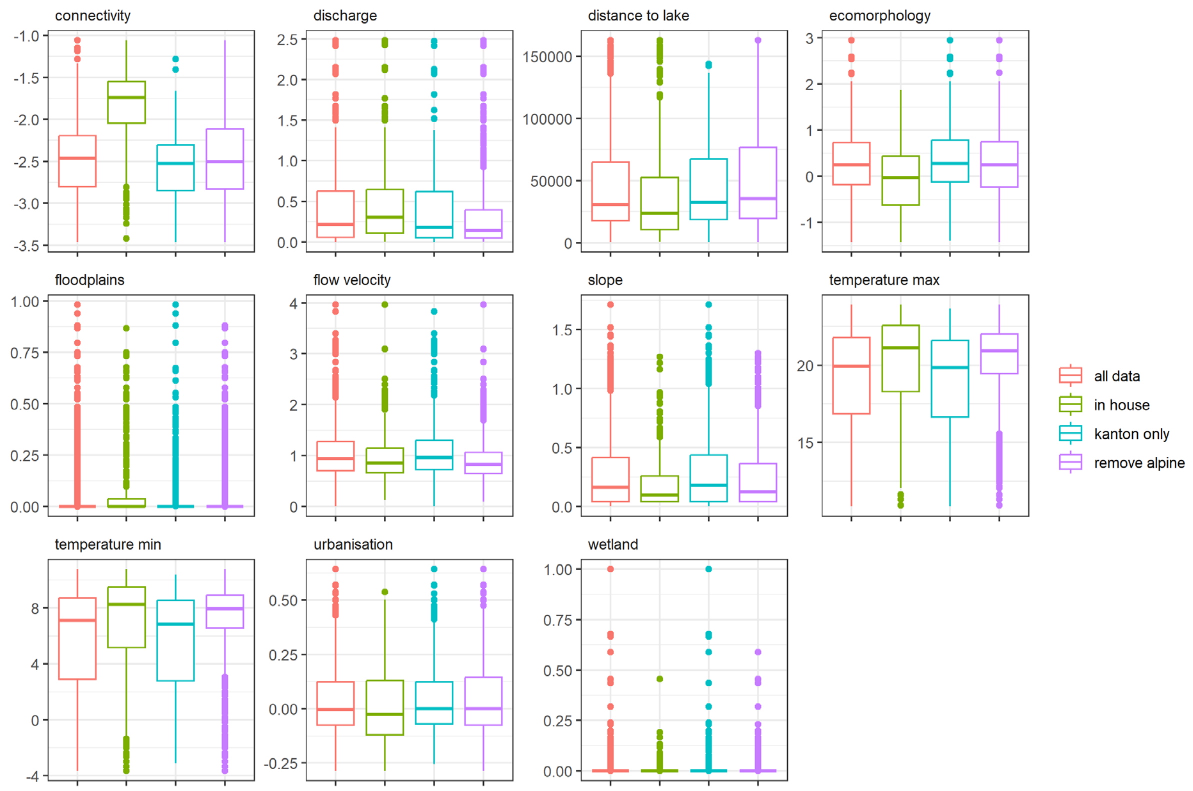

Figure S2. Coverage of environmental data in each data subset used for sensitivity testing. The differential coverage of environmental data supports our decision to combine data sources. Boxplots indicate the median (solid line), inter-quartile range (hinges) and 1.5*IQR (whiskers).

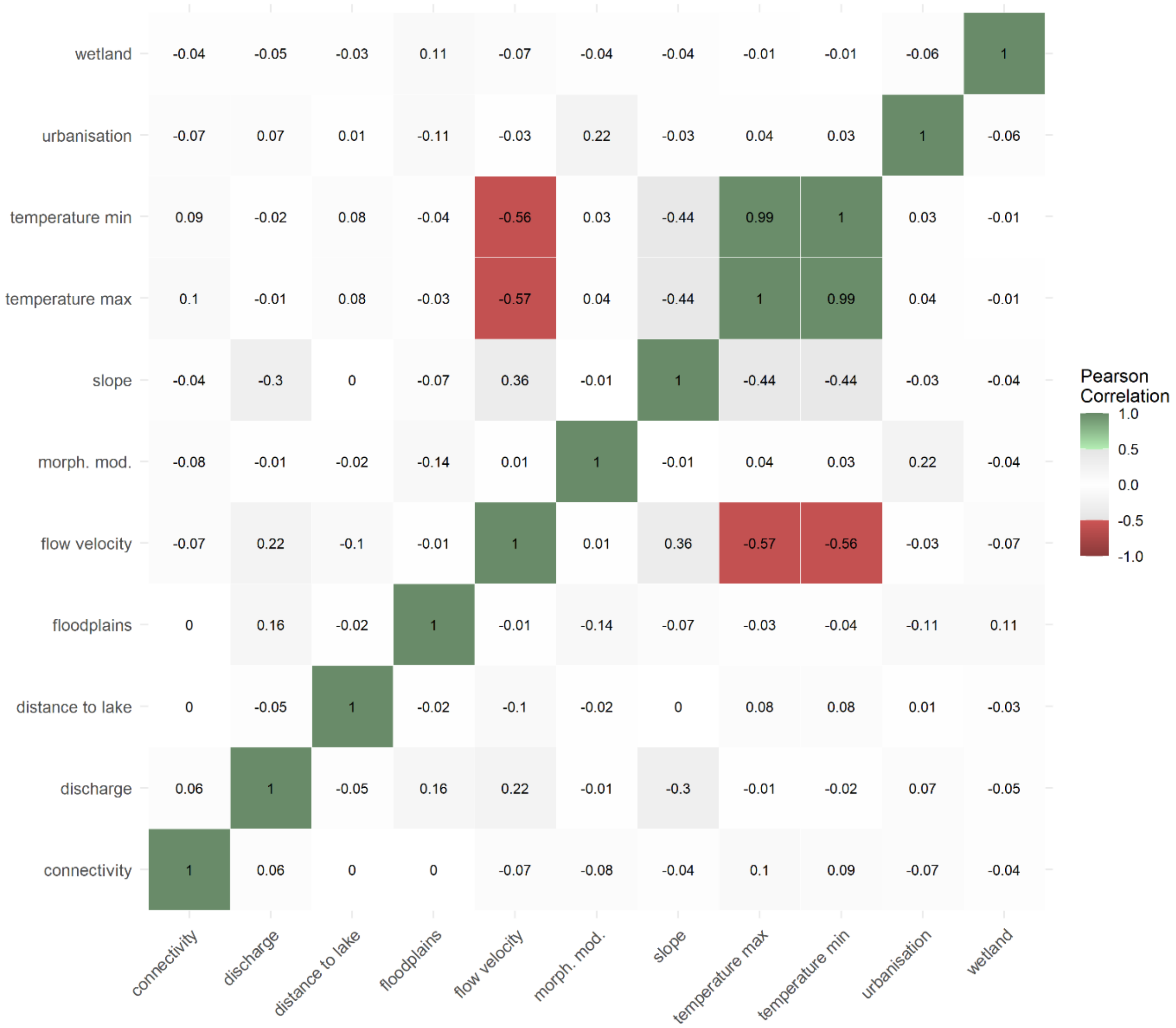

Figure S3. Low correlation among almost all environmental variables used in our species distribution models. Important for our manuscript, there is very low correlation between those which are considered as anthropic threats and those considered as natural abiotic factors. No threat factor is correlated > |0.5| with any natural abiotic factor.

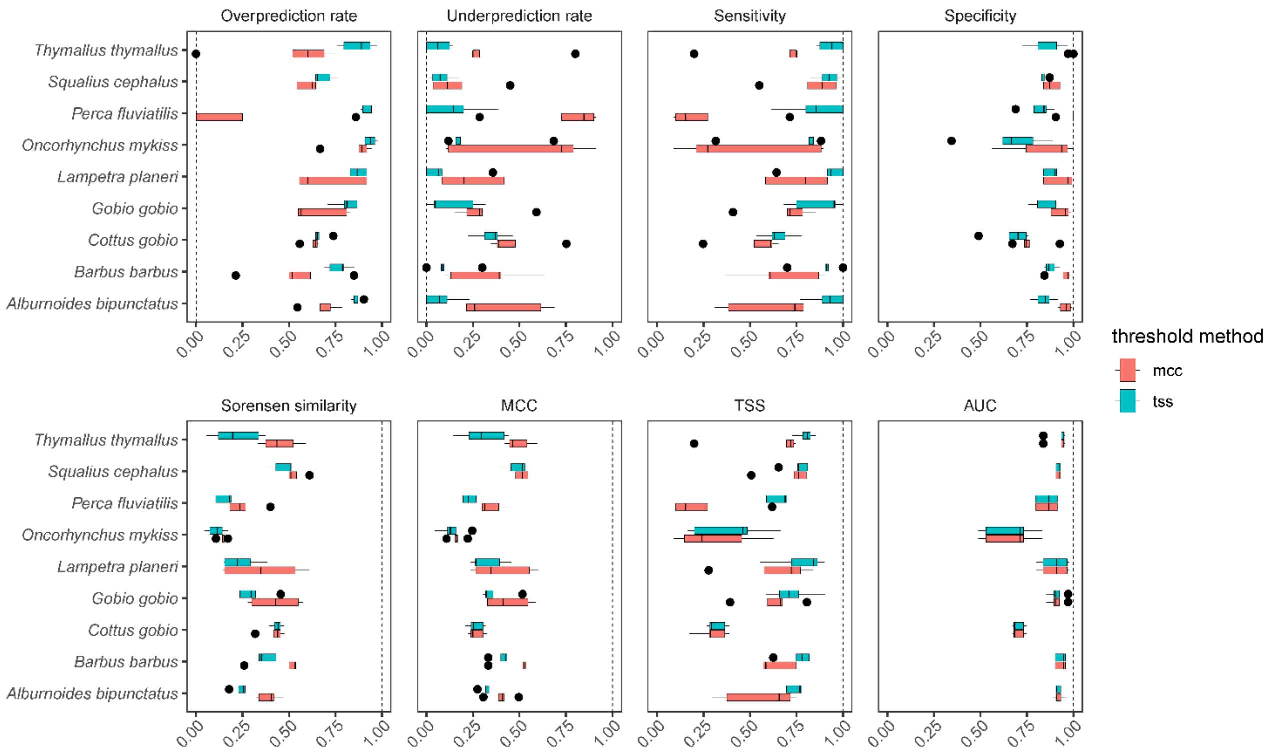

Figure S4. Boxplots of model performance across 8 model evaluation metrics (see Table S2-S3). Colour indicates which metric is optimised under the binary threshold method (either TSS or MCC). Note that AUC is a threshold independent performance metric. Boxplots indicate the median (solid line), inter-quartile range (hinges) and 1.5*IQR (whiskers)

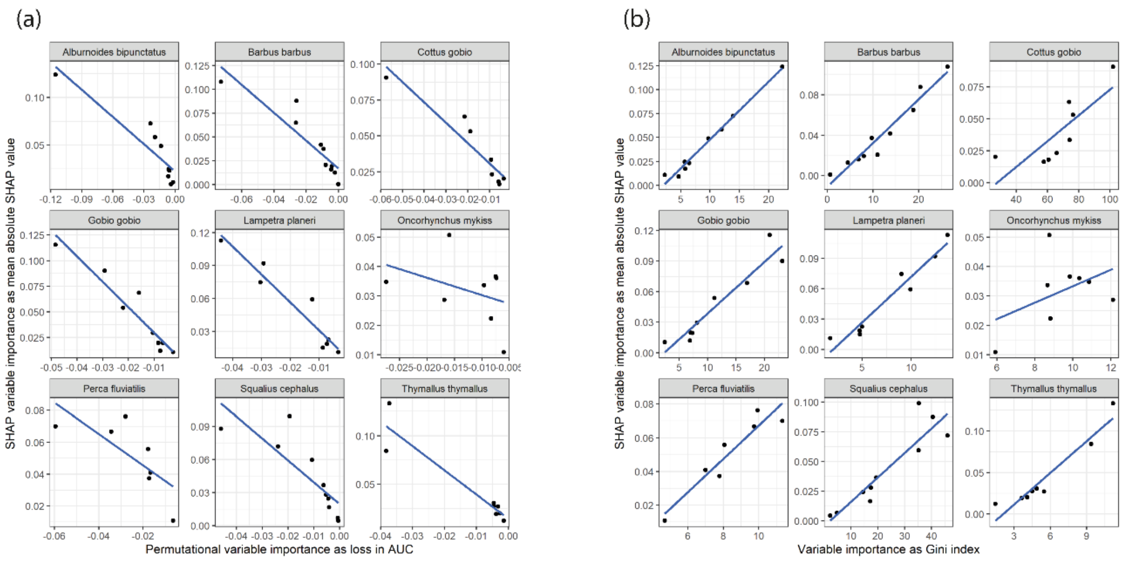

Figure S5. Comparison of SHAP global importance measures with traditional global variable importance measures. We compared SHAP global variable importance with two measures of global variable importance very frequently employed in analyses of random forests and SDMs. We used the model agnostic approach of permutational variable importance, and a random forest specific approach of Gini importance. Permutational variable importance measures the loss of model performance (AUC) when randomising a feature. Gini importance measures the total decrease in node impurity (i.e., improvement in split criteria) averaged over all trees in the ensemble. These measures of variable importance were strongly correlated with SHAP variable importance scores with a Pearson correlation of 0.88±0.24 for Gini index and -0.84±0.24 for permutational importance (negative due to being a loss).

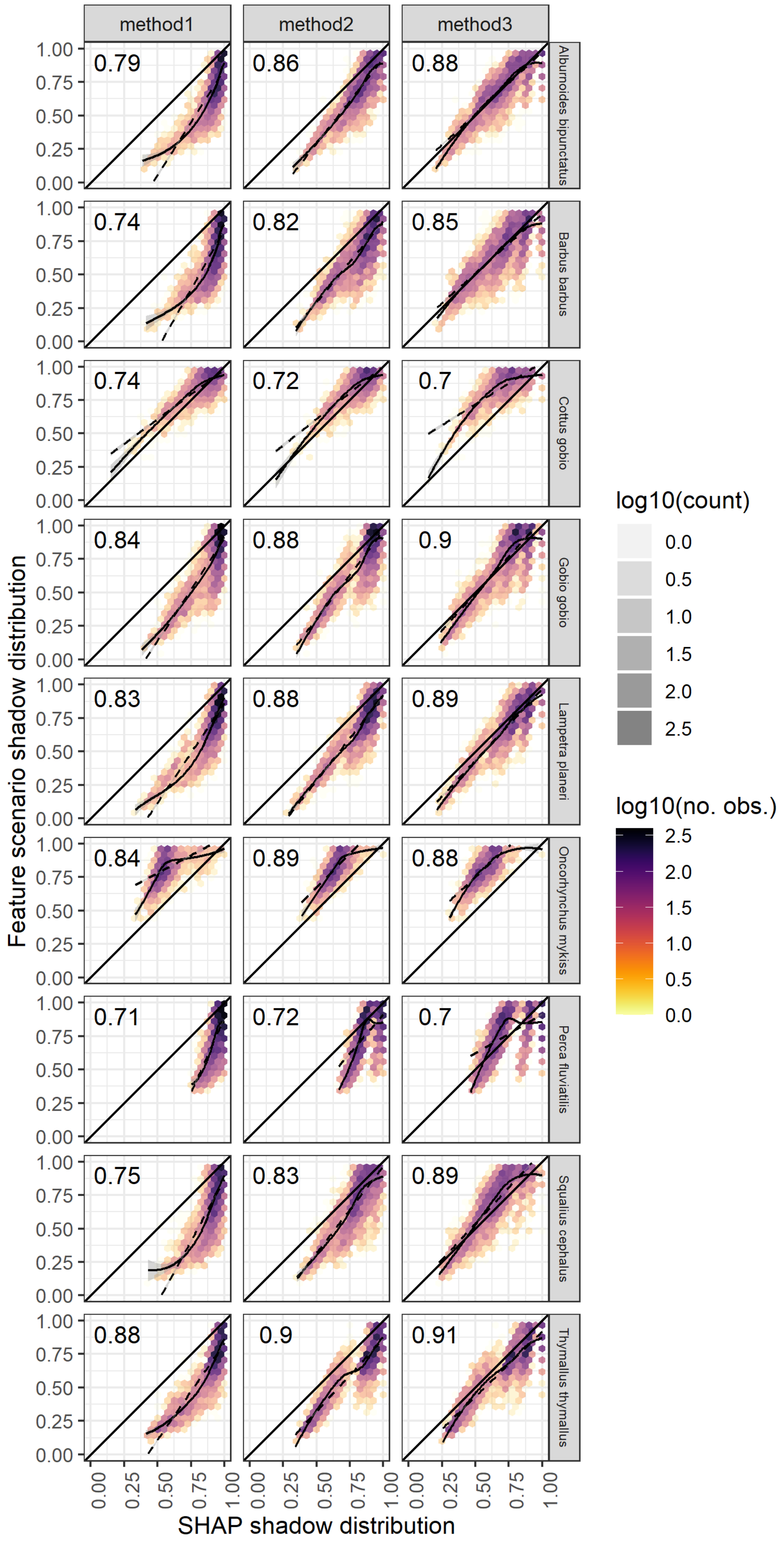

method1
method2
method3
Albumoides bipunctatus
Barbus barbus
Cottus gobio
Gobio gobio
Lampetra planeri
Oncorhynchus mykiss
Perca fluviatilis
Squalius cephalus
Thymallus thymallus
0.79
0.86
0.88
0.74
0.82
0.85
0.74
0.72
0.7
0.84
0.88
0.9
0.83
0.88
0.89
0.84
0.89
0.88
0.71
0.72
0.7
0.75
0.83
0.89
0.88
0.9
0.91
Feature scenario shadow distribution
SHAP shadow distribution
0.00
0.25
0.50
0.75
1.00
log10(count)
0.0
0.5
1.0
1.5
2.0
2.5
log10(no. obs.)
2.5
2.0
1.5
1.0
0.5
0.0

Figure S6. Comparison of shadow distributions estimated through SHAP and predicting habitat suitability of expected distributions after modifying threat features. Shadow distributions are indicated as the observed distribution / expected distribution. These were estimated using SHAP (x-axis) by three approaches ("method3" is presented in the main manuscript). Method1 converts negative SHAP values to 0 which indicates if threats no longer have a negative contribution to environmental suitability (but also do not support environmental suitability). Method2 converts negative SHAP values to the mean positive SHAP values. Method3 converts negative SHAP values to 95th quantile of positive SHAP values for that threat (i.e., best case scenario). The y-axis indicates shadow distributions estimated by simultaneously converting all feature values for threats to new values. In this approach, we replaced environmental values of threat features to be the 99th quantile if a high value represents an improved state, such as higher connectivity, or 1st quantile in the inverse case, such as lower morphological modification. We held non-threat variables at their local value. We then predicted suitability values using these new features to provide the "expected distribution", and compared this to the original habitat suitability predictions as the "observed distribution". This is similar to other scenario-based approaches to biodiversity change, such as modifying temperature under climate change and investigating changes to predicted habitat suitability. Both approaches appear very highly correlated giving a median and inter-quartile range of 0.79 (0.74-0.83) for method 1, 0.86 (0.82-0.87) for method 2, and 0.88 (0.85-0.89) for method 3. This indicates that, while there exists some uncertainty in the estimation of shadow distributions depending on the approach used, there appears strong correspondence between all approaches such that the broad scale summaries of shadow distributions are robust to the approach used.

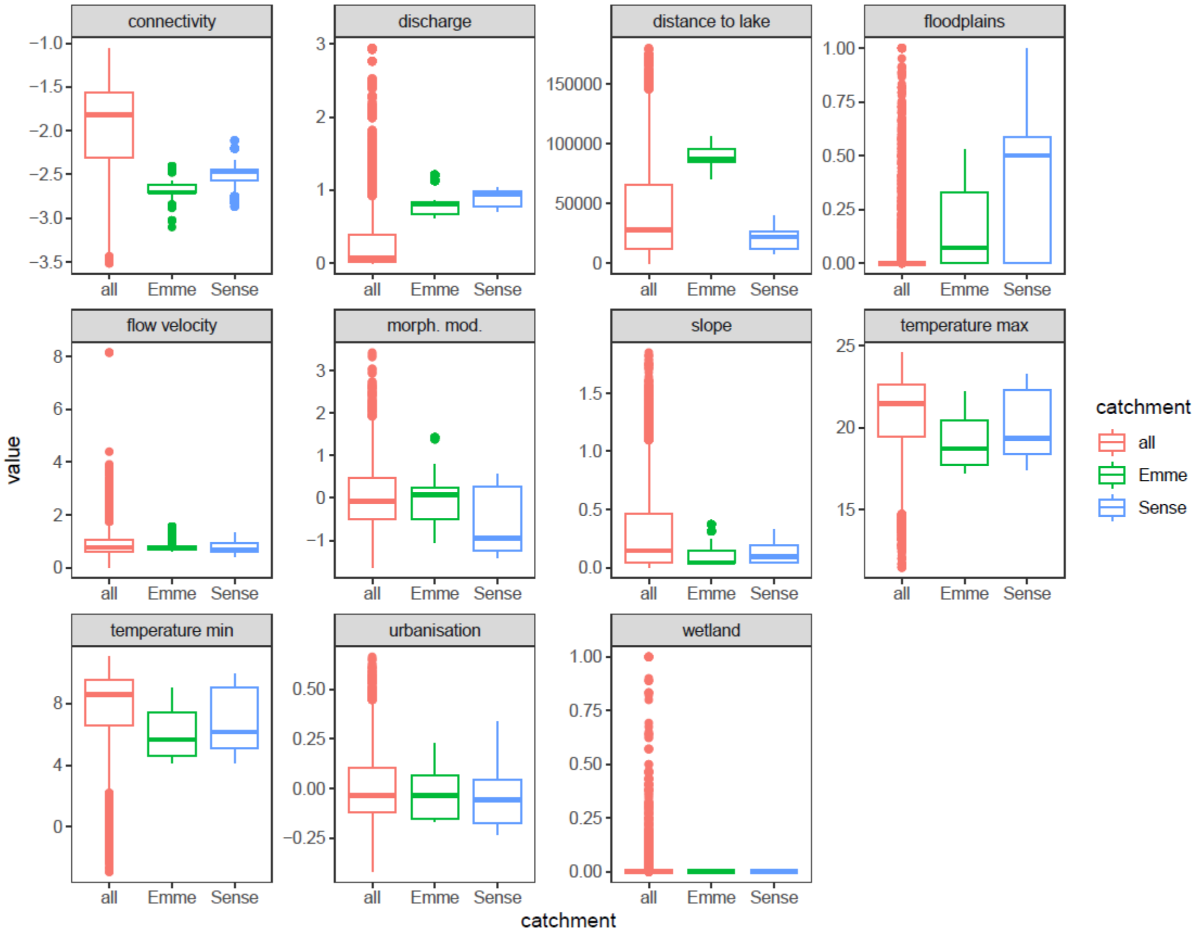

Figure S7. Comparison of environmental properties of Sense and Emme rivers. Boxplots indicate the median (solid line), inter-quartile range (hinges) and 1.5*IQR (whiskers)

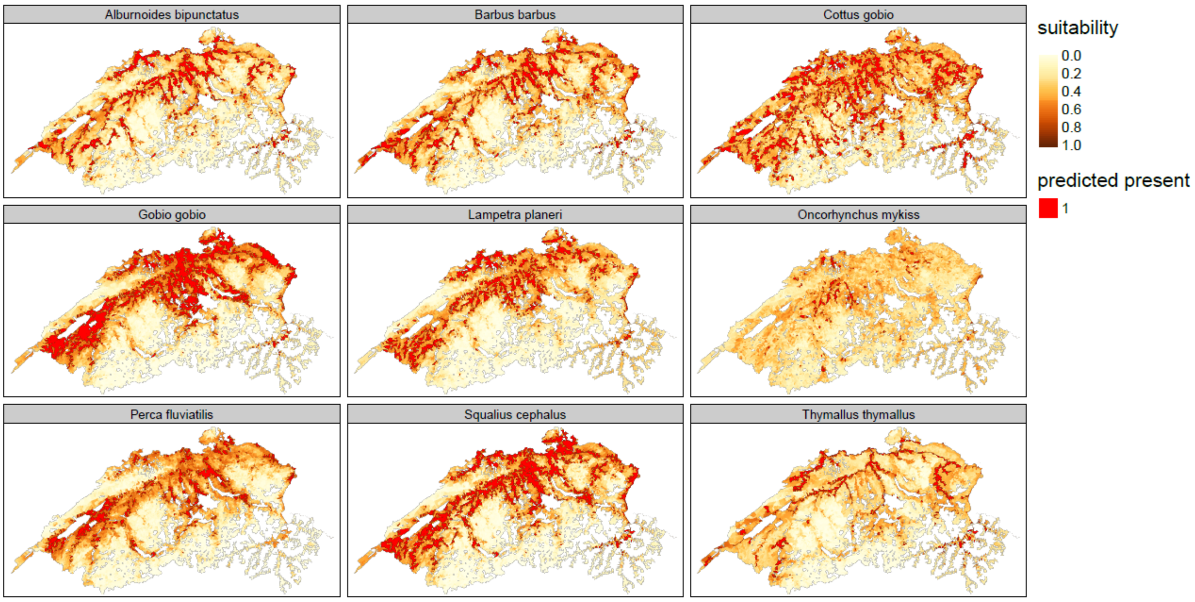

Figure S8. Spatial distribution of habitat suitability for the 9 focal species based on presence-absence random forest models. Red indicates areas where species are predicted as present based on a binary threshold optimising for the True Skill Statistic.

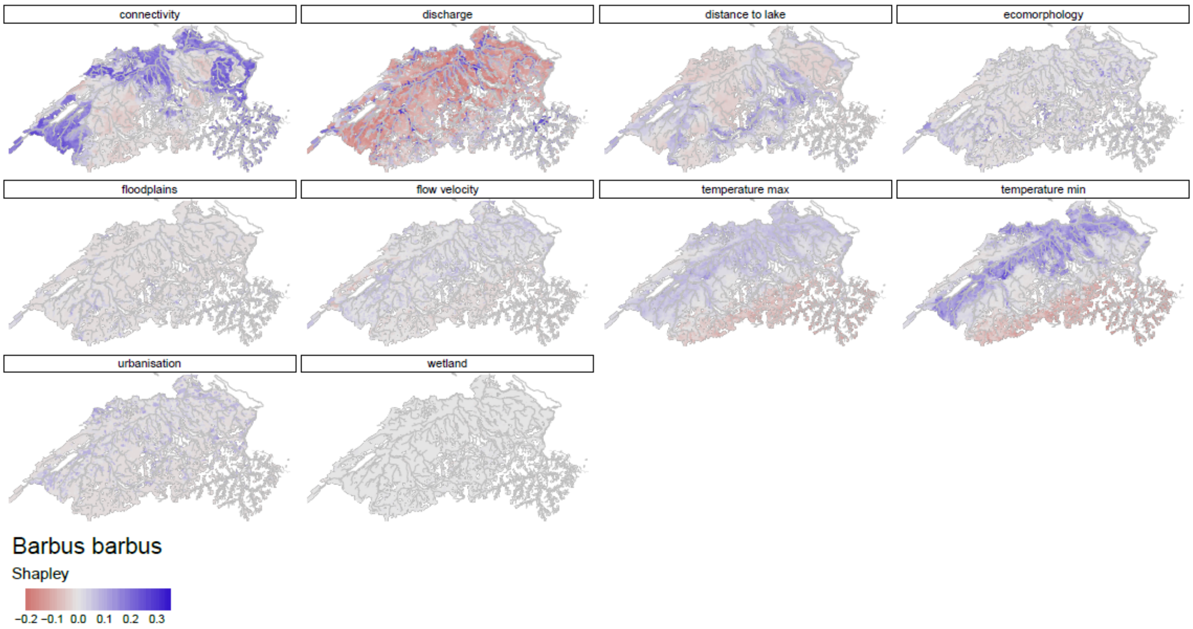

Figure S9. Comparison the spatial distribution of SHAP value distributions for all relevant variables for *Barbus barbus.*

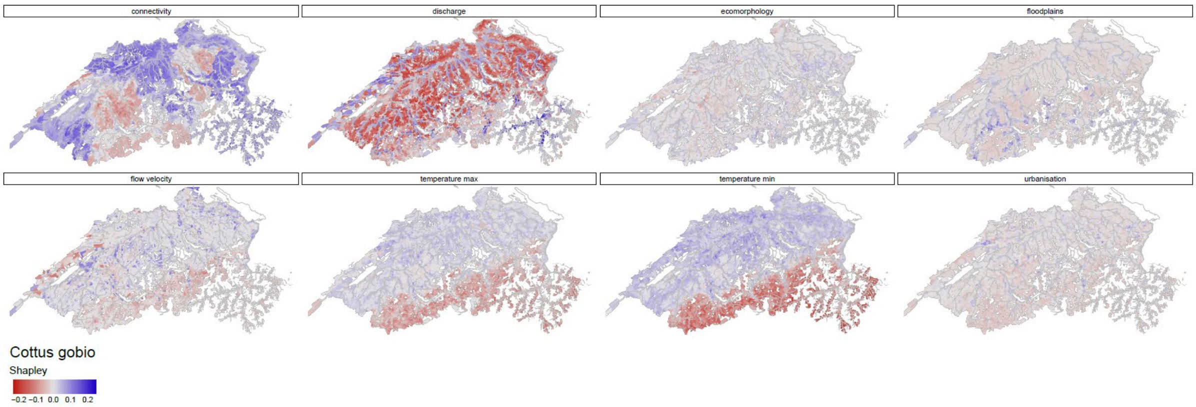

Figure S10. Comparison the spatial distribution of SHAP value distributions for all relevant variables for *Cottus gobio*.

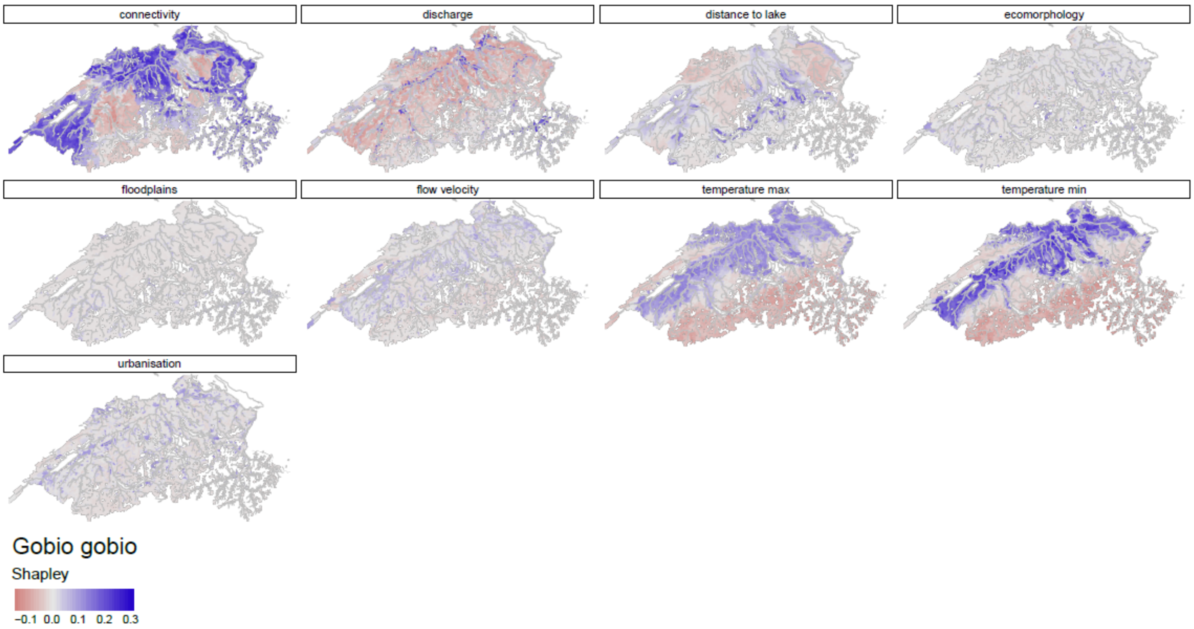

Figure S11. Comparison the spatial distribution of SHAP value distributions for all relevant variables for *Gobio gobio*.

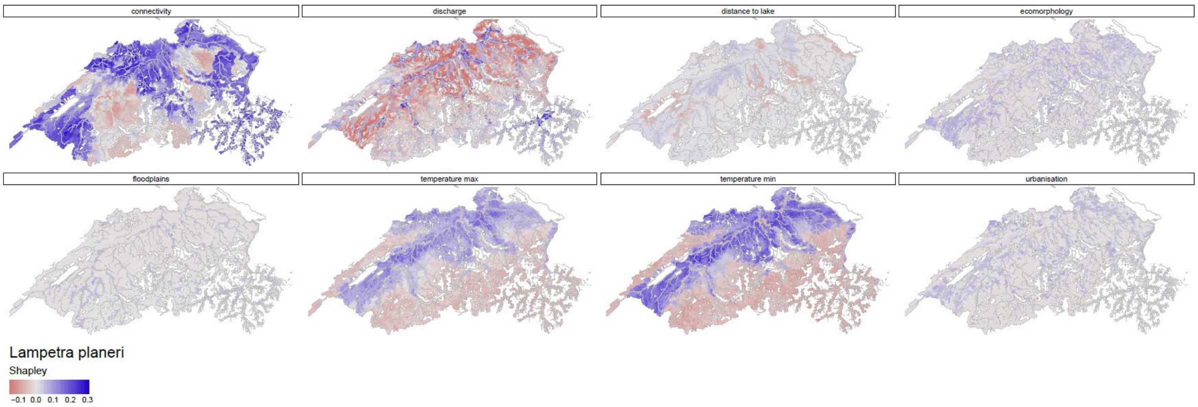

Figure S12. Comparison the spatial distribution of SHAP value distributions for all relevant variables for *Lampetra planeri*.

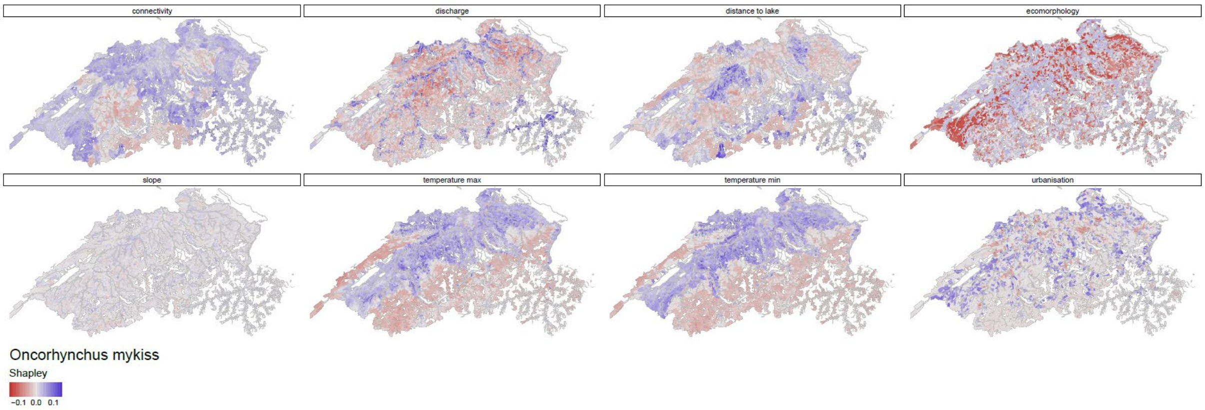

Figure S13. Comparison the spatial distribution of SHAP value distributions for all relevant variables for *Oncorhynchus mykiss*.

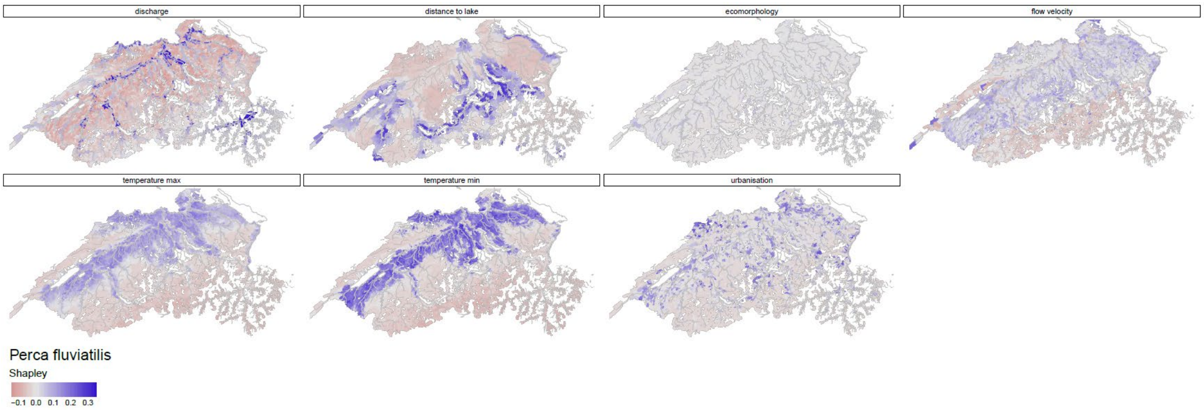

Figure S14. Comparison the spatial distribution of SHAP value distributions for all relevant variables for *Perca fluviatilis*.

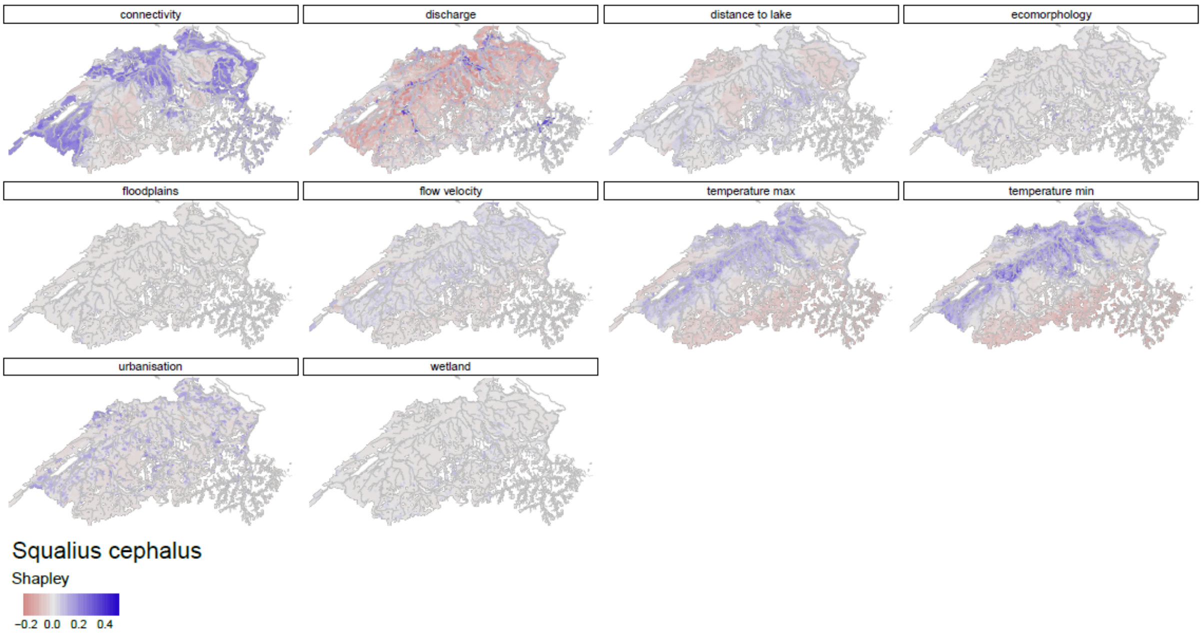

Figure S15. Comparison the spatial distribution of SHAP value distributions for all relevant variables for *Squalius cephalus*.

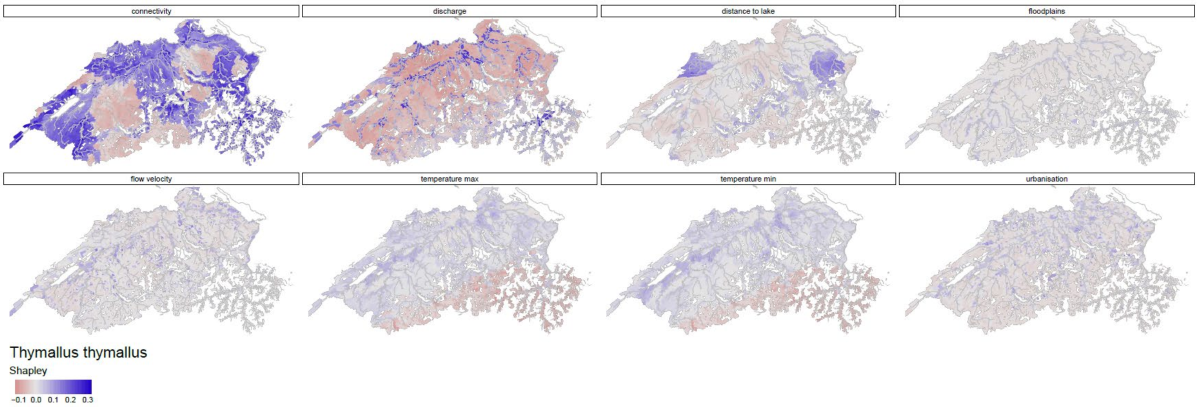

Figure S16. Comparison the spatial distribution of SHAP value distributions for all relevant variables for *Thymallus thymallus*.

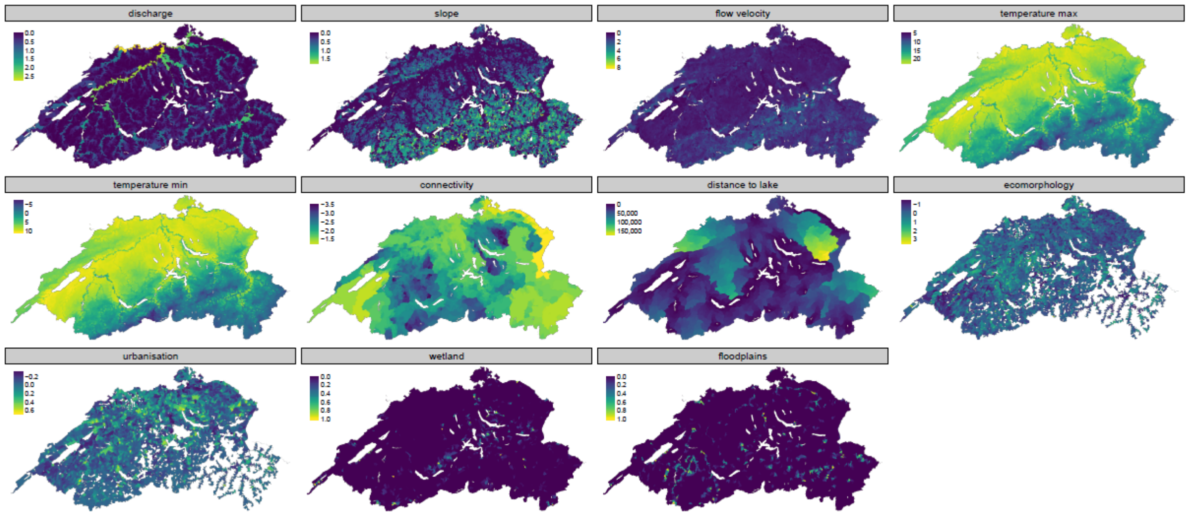

Figure S17. Spatial maps of environmental data used as covariates when fitting species distribution models. Full variable descriptions are provided in our ODMAP protocol (Appendix 1).

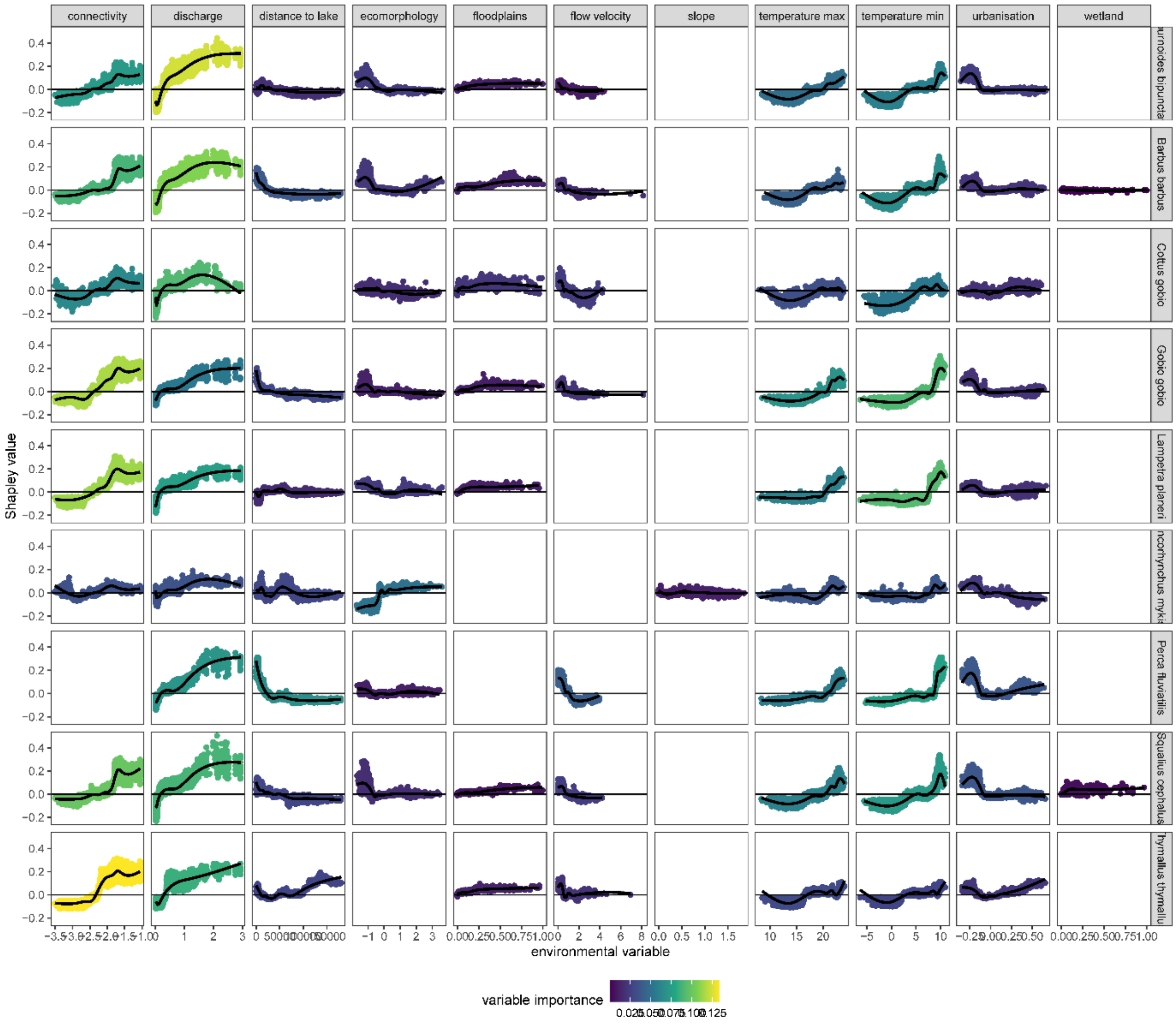

Figure S18. SHAP based response curves across all species and variables. Colour indicates variable importance estimated as the average absolute SHAP value.

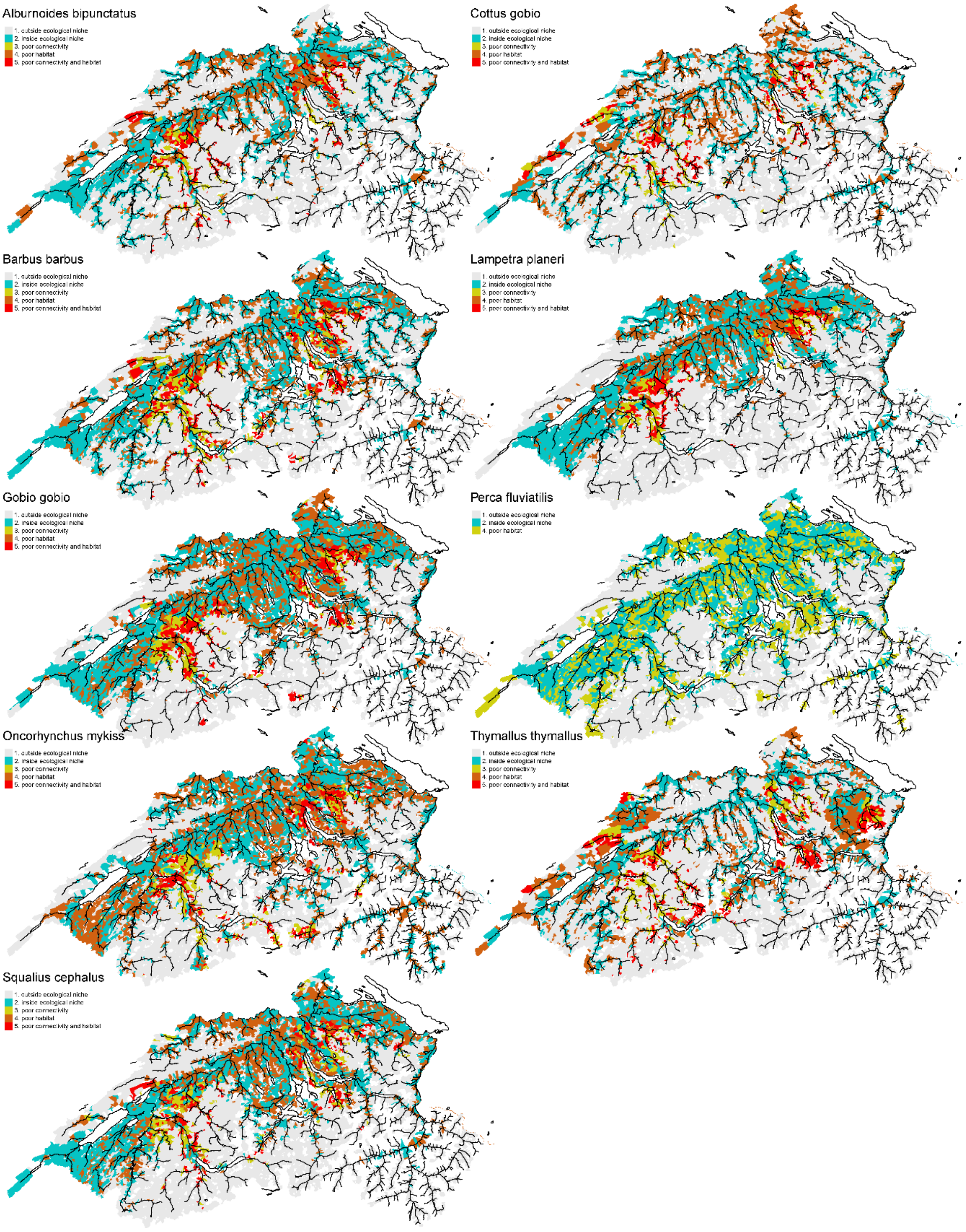

Figure S19. Qualitative shadow distributions for all species to understand the relative influence of anthropic threats within species' expected distributions. For full description see Figure 4 in the main manuscript which provides details for *A. bipunctatus.* Table S3 presents the summary statistics across these distributions.

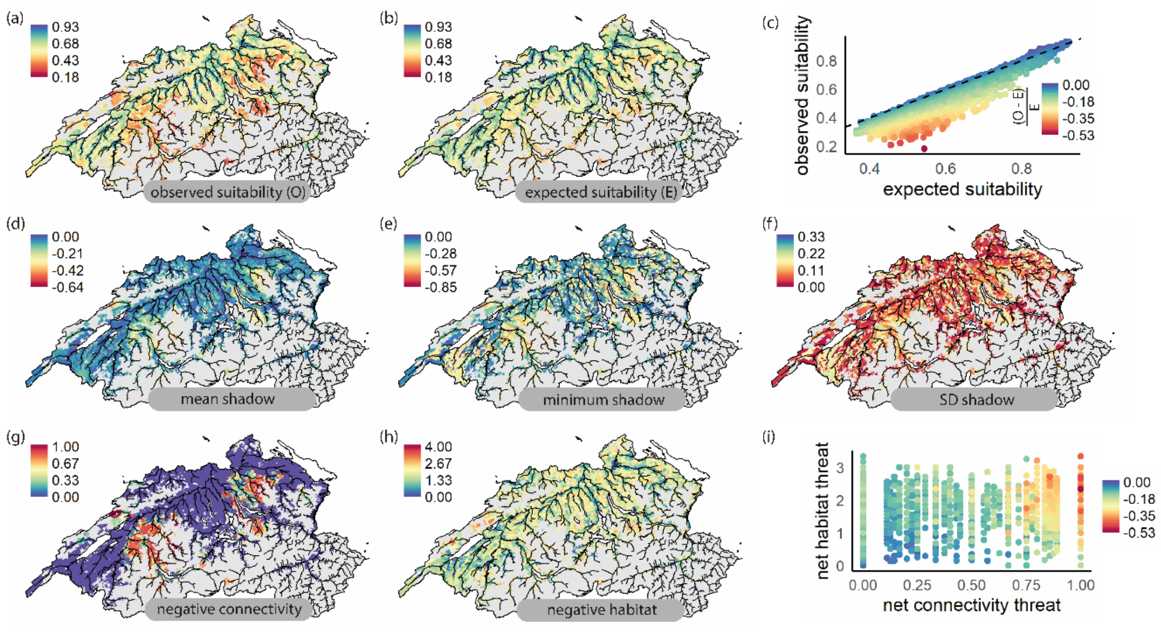

Figure S20. Multi-species average shadow distribution properties per sub-catchment, for full description see Figure 6 of the main manuscript. Here we calculate shadow distributions turning any negative SHAP values for threats to 0 (see methods). To summarise the key results as in the main text for this method Across all sub-catchments, we found environmental suitability in the observed distribution was reduced by 9.4% (averaged across species per sub-catchment = 0.38) compared to the expected distribution (0.47; t = -57; p<0.001). The lowest 10th quantile of catchments had an average suitability reduced by 29%. The most negatively impacted species in each sub-catchment had a habitat suitability reduction of 25% on average across sub-catchments.

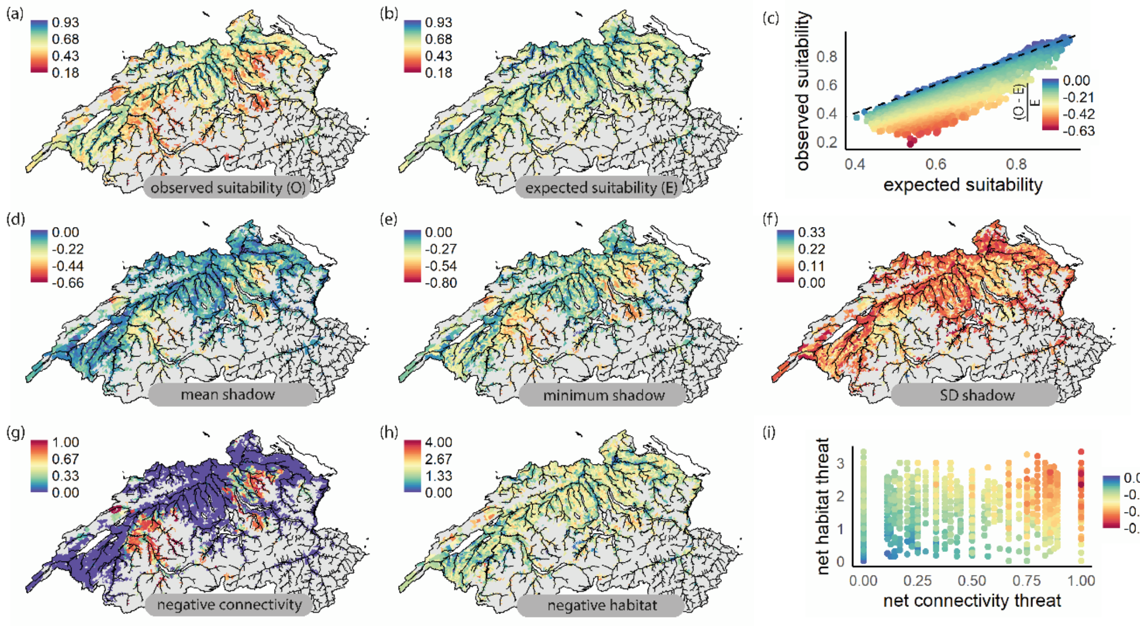

Figure S21. Multi-species average shadow distribution properties per sub-catchment, for full description see Figure 6 of the main manuscript. Here we calculate shadow distributions turning any negative SHAP values for threats to the mean positive contribution across all catchments (see methods). To summarise the key results as in the main text for this method Across all sub-catchments, we found environmental suitability in the observed distribution was reduced by 15% (averaged across species per sub-catchment = 0.38) compared to the expected distribution (0.49; $t = -73$; $p<0.001$). The lowest 10th quantile of catchments had an average suitability reduced by 39%. The most negatively impacted species in each sub-catchment had a habitat suitability reduction of 29% on average across sub-catchments.

Supplementary Tables

Table S1. Overview of species by survey data providing a summary of presence records across different monitoring schemes in our analysis. University of Bern 2022 refers to the summer sampling campaign undertaken by the manuscript authors. Projetto Fiumi was a Switzerland wide river biodiversity monitoring project coordinated by Jakob Brodersen and Ole Seehausen at EAWAG (1). Kanton Bern provided monitoring data undertaken by the fisheries authorities. Consultancies refers to data compiled by Dr. Pascal Vonlanthen and Dr. Sebastien Lauper from various datasets across private and federal surveys and monitoring initiatives (2).

	University of Bern 2022	EAWAG Projetto Fiumi[1]	Kanton Bern	Consultancies[2]	Total
Alburnoides bipunctatus	**10**	**7**	**32**	**61**	**110**
Barbus barbus	**12**	**24**	**34**	**99**	**169**
Cottus gobio	**30**	**59**	**558**	**247**	**894**
Gobio gobio	**11**	**11**	**34**	**79**	**135**
Lampetra planeri	**2**	**2**	**23**	**59**	**86**
Oncorhynchus mykiss	**2**	**3**	**66**	**11**	**82**
Perca fluviatilis	**6**	**8**	**33**	**33**	**80**
Squalius cephalus	**22**	**24**	**100**	**170**	**316**
Thymallus thymallus	**4**	**3**	**27**	**27**	**61**

1. Brodersen, J., Hellmann, J., & Seehausen, O. (2023). *Erhebung der Fischbiodiversität in Schweizer Fliessgewässern. Progetto Fiumi Schlussbericht*. Eawag: Swiss Federal Institute of Aquatic Science and Technology. https://doi.org/10.55408/eawag:30020

2. https://modul-stufen-konzept.ch/en/fish/

Table S2. Summary of species distribution model performance across all data when the threshold between predicted presence and absence is defined by optimizing the Matthews Correlation Coefficient.

Metrics	*Alburnoides bipunctatus*	*Barbus barbus*	*Cottus gobio*	*Gobio gobio*	*Lampetra planeri*	*Oncorhynchus mykiss*	*Perca fluviatilis*	*Squalius cephalus*	*Thymallus thymallus*
**TN**	590 (±51)	580 (±26)	490 (±88)	580 (±94)	570 (±52)	520 (±110)	610 (±41)	520 (±70)	620 (±45)
**FN**	6.4 (±3.4)	8.6 (±6.8)	72 (±25)	6.6 (±3.8)	3.8 (±4)	7.4 (±5.5)	8.4 (±2.7)	7.4 (±7.1)	2.8 (±1.1)
**TP**	10 (±6.1)	16 (±3.7)	82 (±27)	15 (±3.5)	8.6 (±3.2)	8 (±7.4)	3.4 (±3.8)	40 (±12)	6.2 (±4.3)
**FP**	26 (±20)	34 (±42)	140 (±56)	40 (±34)	51 (±58)	100 (±120)	12 (±27)	67 (±30)	10 (±6.7)
**MCC**	0.4 (±0.068)	0.49 (±0.091)	0.27 (±0.043)	0.44 (±0.12)	0.4 (±0.17)	0.16 (±0.041)	0.35 (±0.068)	0.52 (±0.051)	0.49 (±0.072)
**Overprediction rate**	0.68 (±0.088)	0.54 (±0.23)	0.63 (±0.04)	0.66 (±0.15)	0.67 (±0.24)	0.86 (±0.11)	0.22 (±0.37)	0.61 (±0.081)	0.51 (±0.3)
**Underprediction rate**	0.4 (±0.23)	0.33 (±0.22)	0.47 (±0.17)	0.31 (±0.17)	0.28 (±0.29)	0.53 (±0.39)	0.73 (±0.26)	0.16 (±0.17)	0.36 (±0.24)
**Sorensen index**	0.39 (±0.06)	0.48 (±0.12)	0.42 (±0.061)	0.43 (±0.14)	0.36 (±0.21)	0.14 (±0.023)	0.25 (±0.093)	0.52 (±0.061)	0.45 (±0.11)
**Jaccard index**	0.24 (±0.046)	0.32 (±0.096)	0.27 (±0.047)	0.28 (±0.11)	0.24 (±0.16)	0.077 (±0.014)	0.15 (±0.064)	0.35 (±0.056)	0.3 (±0.09)
**Sensitivity**	0.6 (±0.23)	0.67 (±0.22)	0.53 (±0.17)	0.69 (±0.17)	0.72 (±0.29)	0.47 (±0.39)	0.27 (±0.26)	0.84 (±0.17)	0.64 (±0.24)
**Specificity**	0.96 (±0.033)	0.95 (±0.06)	0.77 (±0.095)	0.93 (±0.051)	0.92 (±0.09)	0.84 (±0.19)	0.98 (±0.042)	0.88 (±0.056)	0.98 (±0.011)
**TSS**	0.56 (±0.21)	0.62 (±0.18)	0.3 (±0.084)	0.63 (±0.15)	0.64 (±0.22)	0.31 (±0.22)	0.25 (±0.22)	0.72 (±0.12)	0.62 (±0.24)
**AUC**	0.92 (±0.029)	0.93 (±0.036)	0.7 (±0.035)	0.91 (±0.043)	0.9 (±0.078)	0.66 (±0.14)	0.86 (±0.07)	0.92 (±0.026)	0.92 (±0.049)

Table S3. Summary of species distribution model performance across all data when the threshold between predicted presence and absence is defined by optimizing the True Skill Statistic.

Metrics	*Alburnoides bipunctatus*	*Barbus barbus*	*Cottus gobio*	*Gobio gobio*	*Lampetra planeri*	*Oncorhynchus mykiss*	*Perca fluviatilis*	*Squalius cephalus*	*Thymallus thymallus*
**TN**	520 (±50)	540 (±37)	420 (±80)	530 (±93)	550 (±31)	410 (±120)	510 (±71)	500 (±42)	540 (±74)
**FN**	1.4 (±1.5)	3 (±3.5)	54 (±14)	2.8 (±3)	1.4 (±2.1)	4.4 (±4.8)	1.8 (±2)	3.6 (±2.4)	0.6 (±0.55)
**TP**	15 (±5.3)	21 (±1.7)	100 (±17)	18 (±3.2)	11 (±2.1)	11 (±4.3)	10 (±1.9)	44 (±10)	8.4 (±4.4)
**FP**	97 (±36)	75 (±24)	210 (±74)	89 (±44)	77 (±34)	210 (±120)	110 (±47)	94 (±13)	85 (±59)
**MCC**	0.32 (±0.033)	0.41 (±0.053)	0.27 (±0.044)	0.36 (±0.087)	0.32 (±0.095)	0.14 (±0.073)	0.24 (±0.056)	0.49 (±0.064)	0.3 (±0.13)
**Overprediction rate**	0.86 (±0.027)	0.77 (±0.064)	0.67 (±0.04)	0.81 (±0.066)	0.86 (±0.067)	0.94 (±0.032)	0.91 (±0.029)	0.68 (±0.054)	0.87 (±0.09)
**Underprediction rate**	0.083 (±0.096)	0.11 (±0.11)	0.35 (±0.09)	0.13 (±0.14)	0.1 (±0.15)	0.26 (±0.24)	0.15 (±0.16)	0.082 (±0.063)	0.065 (±0.067)
**Sorensen index**	0.24 (±0.04)	0.36 (±0.072)	0.44 (±0.03)	0.31 (±0.09)	0.24 (±0.1)	0.11 (±0.051)	0.16 (±0.048)	0.47 (±0.067)	0.22 (±0.14)
**Jaccard index**	0.14 (±0.025)	0.22 (±0.054)	0.28 (±0.024)	0.18 (±0.066)	0.14 (±0.066)	0.058 (±0.029)	0.086 (±0.028)	0.31 (±0.056)	0.13 (±0.087)
**Sensitivity**	0.92 (±0.096)	0.89 (±0.11)	0.65 (±0.09)	0.87 (±0.14)	0.9 (±0.15)	0.74 (±0.24)	0.85 (±0.16)	0.92 (±0.063)	0.93 (±0.067)
**Specificity**	0.84 (±0.057)	0.88 (±0.034)	0.67 (±0.11)	0.86 (±0.071)	0.88 (±0.052)	0.66 (±0.2)	0.81 (±0.08)	0.84 (±0.019)	0.86 (±0.096)
**TSS**	0.76 (±0.074)	0.76 (±0.088)	0.32 (±0.054)	0.72 (±0.12)	0.78 (±0.14)	0.4 (±0.21)	0.67 (±0.13)	0.76 (±0.064)	0.8 (±0.048)
**AUC**	0.92 (±0.029)	0.93 (±0.036)	0.7 (±0.035)	0.91 (±0.043)	0.9 (±0.078)	0.66 (±0.14)	0.86 (±0.07)	0.92 (±0.026)	0.92 (±0.049)

Table S4. Summaries of species shadow distribution properties as an average and standard deviation across all species.

Property of SHAP values and/or shadow distribution	Mean	SD
% all subcatchments with a positive contribution of discharge	36	4.36
% all subcatchments with a positive contribution of flow velocity	53.43	7.98
% all subcatchments with a positive contribution of slope	53	
% all subcatchments with a positive contribution of distance to lake	50	7.95
% all subcatchments with a positive contribution of minimum temperature	55.89	12.55
% all subcatchments with a positive contribution of maximum temperature	56.22	8.87
% of subcatchments inside niche with 0 threats negative	12.44	8.53
% of subcatchments inside niche with 1 threats negative	32.44	10.6
% of subcatchments inside niche with 2 threats negative	32	6.34
% of subcatchments inside niche with 3 threats negative	18.62	8.38
% of subcatchments inside niche with 4 threats negative	8.33	6.74
% of subcatchments inside niche with 5 threats negative	4	0
% reduction in suitability in threatened areas within niche	-24.78	6.98
% sub-catchments inside ecological niche	45.44	7.13
% sub-catchments with positive SHAP values for all natural niche variables	7	2.29
% subcatchments inside niche with a negative contribution of river morphological modification	45.25	5.2
% subcatchments inside niche with a negative contribution of connectivity	17.62	4
% subcatchments inside niche with a negative contribution of floodplain proportion	70.29	7.41
% subcatchments inside niche with a negative contribution of urbanisation	50.89	5.4
% subcatchments inside niche with a negative contribution of wetland proportion	49.5	23.33
% subcatchments inside niche with a negative net effect of all threats summed	22.78	12.43
% subcatchments inside niche with at least one negative threat	87.56	8.53
% threatened catchments in niche with predicted absence	78.56	15.74
% unthreatened catchments in niche with predicted absence	50.11	26.21
mean suitability in threatened sub-catchments	0.48	0.07
mean suitability in unthreatened sub-catchments	0.63	0.08
number of times more absences in threatened compared to unthreatened base rate	2.04	1.18

Supplementary Methods

Here we provide an ODMAP protocol to improve reporting of species distribution modelling and ensure transparency and checking that our models are fit-for-purpose[1–4].

ODMAP protocol

Section 1: Overview

Authorship

Authors: Conor Waldock, Bernhard Wegscheider, Dario Josi, Barbara Calegari, Jakob Brodersen, Luiz Jardim de Queiroz, Ole Seehausen

Contact: conor.waldock@unibe.ch, conorwaldock@gmail.com

Webpage: *https://github.com/wyss-swiss-fish/xAI-demonstration*

Model objective

Model objective: We aimed to model the spatial distribution of fish species in Switzerland, identify the key factors explaining species distributions, and apply model-agnostic locally explainable artificial intelligence approaches to explain the how environmental factors spatially structure species geographic ranges.

Focal Taxon

Alburnoides bipunctatus, Barbus barbus, Cottus gobio, Gobio gobio, Lampetra planeri, Oncorhynchus mykiss, Perca fluviatilis, Squalius cephalus, Thymallus thymallus

Location

Switzerland.

Scale of Analysis

Spatial extent: Aare, Limmat, Reuss and Rhine catchments that form the majority of Switzerland's river network north of the Alps.

Latitude: 6.053483 to 10.230689 (EPSG:4326, WGS84)

Longitude: 46.325695 to 48.023463 (EPSG:4326, WGS84)

Temporal extent: Presence-absence records spanning from 2010 to 2023.

Boundary: Political and watershed boundaries of Switzerland.

Biodiversity data

Observation type: Presence-absence records came from quantitative and semi-quantitative field surveys conducted by scientific researchers, cantonal and national monitoring agencies, ecological consultancies.

Response data type: Presence-absence (0s and 1s).

Predictors

Predictor types: Bioclimate, habitat quality, water quality, flow regime, land-use, connectivity (see Table 3 for full details).

Hypotheses

Species environment relationships were expected to be multidimensional with multiple important factors that show spatial variation across species ranges. We expected variation between species in

responses to environmental factors. We expected relatively consistent negative effects of human impacts but with a few species that could benefit from human related factors, most likely non-native species. Generally, we expected positive effects of temperature on warmer affinity families (Cyprinids, Perciformes) with biogeographic histories of post-glacial alpine recolonization from warm refugia. We expected that agriculturally dominated lowland Switzerland may be particularly depleted in local biodiversity, and therefore have low predicted habitat suitability, due to the combined effects of land-use change, water pollution, and river morphological modification, despite having higher potential for biodiversity because rivers exhibit higher discharge, shallow slopes, slower flows, and warmer water temperatures. We expected at cold-range edges temperature will be a dominant structuring factor of species geographic distributions, however, we expected the capacity for species to exist at their natural range limits was altered by natural and anthropic barriers that reduced connectivity of these river stretches.

Assumptions

Relevant biological, ecological, and human-related drivers of species distributions, or proxy variables, were included.

Sampling was adequate and representative of the biological, ecological, and human-related drivers of species distributions.

Quantitative surveys had few important detection errors, these did not covary spatially, or covary with environmental conditions.

Species were at equilibrium with their environment (no dispersal limitation beyond those included as covariates).

Biological interactions had a limited influence on species distributions.

Algorithms

Modelling techniques: We used down-sampled random forests as fit in [5] and shown to be one of the best models of species' spatial distributions in a large benchmarked review of presence-only species distribution models [6]. These models are designed to overcome the class imbalance issue often associated with over-fitting in random forest models, for full descriptions see [5].

Model complexity: Random forests are complex algorithms but the down-sampling approach is designed to avoid overfitting that can occur in random forests due to class imbalance (many more 0s than 1s) and lead to poor out-of-sample performance. We used a low number of trees (1000) and standard 'mtry' parameter of the square root of the number of variables to further avoid overfitting.

Model averaging: None applied

Workflow

Model workflow: We extracted the environment at the coordinates of all presence-absence records. To this data matrix, we fitted down-sampled random forests. Given the environmental conditions of 2km sub-catchments across the Aare, Limmat, Reuss and Rhine river network (within the borders of Switzerland) we made predictions, given the fitted random forest. This provided a prediction of habitat suitability per sub-catchment per species. The performance of all models was cross-validated by spatially blocking our data into 5-folds. For the predicted habitat suitability in every 2km sub-catchment we applied SHAPLEY analysis, which assessed the local contribution of environmental variables to species habitat suitability prediction.

Software

Software: R version 4.1.0 (2021-05-18), randomForest 4.7-1.1

Code availability: https://github.com/wyss-swiss-fish/xAI-demonstration

Data availability: Data came from a variety of sources with different public availability. Projetto Fiumi data are available on request from Dr. Jakob Brodersen at EAWAG. Dr. Pascal Vonlanthen provided a compiled dataset of private fish monitoring data compiled for Switzerland. Kanton Berne data are

available on request from the responsible authorities. The authors of this document performed electrofishing surveys across the Aare catchment and data are available on request from the authors.

Section 2: Data

Biodiversity data

Taxon names: Taxa see Table 1 below.

Taxonomic reference system: We updated all taxonomic information based on primary literature and genetic barcoding of specimens from work completed at EAWAG and University of Bern in recent years to better delimit species distributions [7,8]. Note that "*Cottus gobio"* and "*Perca fluviatilis*" have high phenotypic and genetic variation between populations in Switzerland indicating distinct post-glaciation colonization waves [9,10] and likely presence of multiple unrecognized species [8].

Ecological level: Population level (i.e., assuming records come from independent populations and variation between populations in relative habitat suitability depends on environmental conditions).

Biodiversity data sources:

- *Project fieldwork data (CW, DJ, BW, BC, OS):* Data were collected in the context of the University of Bern, Federal Office for the Environmental, Kanton Bern and Wyss Academy for Nature LANAT-3 project titled "*Stopping the biodiversity loss of water bodies - despite climate change*". Data were collected under the Swiss animal experimentation licence (Nr national: 34546; Nr: BE11/2022).

- *Projetto Fiumi:* The main objective of Progetto Fiumi (Erhebung der Fischbiodiversität in Schweizer Fliessgewässern) was to survey fish biodiversity and its distribution in Swiss rivers and surveys were undertaken between 2013 and 2017, for full details see [7].

- *Module Stufen Konzept electrofishing data:* Compiled by Dr. Pascal Vonlanthen and Dr. Sébastien Lauper in the context of compiling data collected under the biomonitoring protocol 'Module Stufen Konzept' (https://modul-stufen-konzept.ch).

- *Kanton Berne electrofishing data:* The Canton of Bern has provided us with an extract from its fisheries database for the period 1994 to 2022.

See Tables 1 and 2 for an overview of species presence records and available data from different monitoring datasets.

Table 1. Summary of presence records across different monitoring schemes in our analysis. University of Bern 2022 refers to our summer sampling campaign undertaken by (BC, BW, CW, DJ, OS). Projetto Fiumi was a Switzerland wide river biodiversity monitoring project coordinated by Jakob Brodersen and Ole Seehausen at EAWAG (1). Kanton Bern provided monitoring data undertaken by.. Consultancies refers to data compiled by Dr. Pascal Vonlanthen and Dr. Sebastien Lauper compiling various surveys from private and federal surveys and monitoring initiatives conforming to standardised electrofishing surveys (2).

	University of Bern 2022	EAWAG Projetto Fiumi	Kanton Bern	MSK	Total
Alburnoides bipunctatus	10	7	32	61	110
Barbus barbus	12	24	34	99	169
Cottus gobio	30	59	558	247	894
Gobio gobio	11	11	34	79	135
Lampetra planeri	2	2	23	59	86
Oncorhynchus mykiss	2	3	66	11	82
Perca fluviatilis	6	8	33	33	80
Squalius cephalus	22	24	100	170	316
Thymallus thymallus	4	3	27	27	61

Table 2. Summaries of records in across databases.

	Number of species	Number of locations	Number of populations	First year	Median year	Last year	95th-percentile year
**1. University of Bern 2022**	9	38	99	2022	2022	2022	2022
**2. EAWAG Projetto Fiumi**	9	70	141	2013	2014	2017	2013
**3. Kanton Bern**	9	655	871	2010	2016	2022	2010
**4. Module Stufen Konzept**	9	215	570	2010	2018	2020	2011

Sampling design:

- *Project fieldwork data (DJ, BW, BC, CW, OS):* Electrofishing campaign from August to October 2022. We stratified sampling of sites based on 9 classes combining three human impacts levels (low, medium, high) and three elevation strata (low, medium, high) based on equal sized strata. From these strata, we sampled 46 sites and attempted to balance site selection across strata. We fished 100 meter stretches of wadeable streams with electrofishing at 46 sites in Switzerland. All fish were identified in the field, as best as possible, to species level, measured, and weighed. The determination of species from difficult groups was later confirmed in the laboratory or on the basis of the photos. Approximately 10-15 individuals of all species (license permitting) were retained to form long-term research collections at the Naturhistorisches Museum Bern.

- *Projetto Fiumi:* Sites were surveyed between 2013 and 2017. Site selection was intended to representatively survey the diversity of Swiss watercourses. Surveys were conducted in September and October each year. A stretch of approximately 100 meters was quantitatively fished when rivers were small. Waterbodies that were too wide, too deep, or too fast-flowing were fished qualitatively, taking into account all habitats as much as possible. Non-wadeable, large rivers were sampled by boat equipped with an anode rake using the strip fishing method. Captured fish were stocked separately by strip, or by passage in the case of quantitative surveys. All fish were identified in the field, as best as possible, to species level, measured, and weighed. The determination of species from difficult groups was later confirmed in the laboratory or on the basis of the photos.

- *Module Stufen Konzept electrofishing data:* These data were compiled if they conformed to the electro-fishing component of the 'Module Stufen Konzept' (modular stepwise procedure) providing methods for the analysis and assessment of surface waters in Switzerland (https://modul-stufen-konzept.ch). These methods are developed to ensure assessment of watercourse status is in accordance with water protection legislation and was developed in collaboration with the federal government, cantonal government, EAWAG and the VSA. All data provided in this compilation had at least two electrofishing 'runs' recording all fish species encountered with the aim to describe the present species community (personal communication Dr. Pascal Vonlanthen and Dr. Sebastien Lauper).

- *Kanton Bern electrofishing data:* This mainly contains data collected at all construction sites during this time-period as well as some data from long-term monitoring sites.

Sample size:

Alburnoides bipunctatus (110); Barbus barbus (169); Cottus gobio (894); Gobio gobio (135); Lampetra planeri (86); Oncorhynchus mykiss (82); Perca fluviatilis (80); Squalius cephalus (316); Thymallus thymallus (61)

Absence data:

Combining all surveys together, we obtain 3,216 sampling events that provided absence records if species were not recorded on a survey.

- *Project fieldwork data (CW, BW, DJ, BC, OS):* As far as logistically possible, we applied standardized fishing protocol that had to be adapted depending on river width, flow velocities and depth. Given that we collected all observed individuals and identified these at a species level we assume that missing species are absent from sampling events, and therefore sites.

- *Projetto Fiumi:* For absence data, we filtered Projetto Fiumi data to only quantitative and semi-quantitative sampling events, rather than non-systematic qualitative sampling events. Given that all observed individuals were identified at a species level we assume that missing species are absent from sampling events, and therefore sites.

- *Module Stufen Konzept electrofishing data:* All data provided in this compilation had at least two electrofishing 'runs' recording all fish species encountered with the aim to describe the present species community and as such we assumed all missing species were absent from sampling events.

- *Kanton Bern electrofishing data:* As far as we are aware, all species recovered were reported in electrofishing events, however, sampling occurred for different purposes depending on the monitoring needs that may introduce differences in the detectability of different species.

Background data: We used presence-absence data, so no background data were generated.

Data partitioning

Training data: To evaluate our models, we split our presence-absence datasets into 5-fold spatially blocked cross validation sets using the blockCV package in R (version 2.1.4; [11]). We set the size of blocks to 10 km which was identified as the spatial scale of spatial autocorrelation in our environmental variables during preliminary analyses in our predictor set. We optimized our blocking grid to provide the most equal split in presence-absence across folds selected blocks, and arranged these blocks randomly.

Validation data: We did not use external validation data, but instead validated our models on the held-out cross validation set described above.

Predictor variables

For spatial distributions of relevant predictor variables see Figure S14 in Appendix 2. These are further summarized in detail below in Table 3. Our final models included 11 potential variables for model fitting, see Section 3 on model fitting below for further details on variable selection.

Table 3. Relevant details on predictor variable and processing steps. Coordinate reference systems were different amongst data products and harmonized to epsg:3035 equal area projection of Europe (ETRS89-extended / LAEA Europe). Final predictors used in our models are highlighted in bold.

Variable	Unit	Source	Time period	Spatial scale raw data	Temporal aggregation	Spatial scale in model	Spatial aggregation	Transformation
**Monthly minimum temperature**	**degrees C**	**CHELSA V2.1 climatology**	**1981-2010**	**1km**	**Minimum across years**	**STARS entire upstream catchment**	**NA**	**NA**
**Monthly maximum temperature**	**degrees C**	**CHELSA V2.1 climatology**	**1981-2010**	**1km**	**Maximum across years**	**STARS entire upstream catchment**	**NA**	**NA**
Monthly mean temperature	degrees C	CHELSA V2.1 climatology	1981-2010	1km	Mean across years	STARS entire upstream catchment	NA	NA
**Maximum discharge**	**m3/s**	**MQ-GWN-CH**	**1981-2000**	**river reach**	**Mean across years**	**BAFU subcatchment**	**Maximum per subcatchment**	**Log10+1**
**Minimum slope**	**%**	**swissALTI3D**	**NA**	**river reach**	**NA**	**BAFU subcatchment**	**Minimum per subcatchment**	**Log10+1**
**Flow velocity**	**m/s**	**derived**	**NA**	**river reach**	**NA**	**BAFU subcatchment**	**Mean weighted by river length per subcatchment**	**log+0.01**
**River morphological modification**	**Unitless index**	**BAFU Ecomorphology-F**	**2013-2016**	**river reach**	**NA**	**BAFU subcatchment**	**Mean weighted by river length per subcatchment**	**NA**

Cropland cover	%	Corine Land Cover (CLC) 2018, Version 2020_20u1	2017-2018	25ha	NA	STARS reach contributing area	Proportion cover	NA
**Floodplain**	**presence-absence**	**BAFU**	**2017**	**Polygon of object**	**NA**	**Local**	**Mean coverage per sub-catchment**	**NA**
**Wetland**	**Presence-absence**	**BAFU**	**2017**	**Polygon of object**	**NA**	**Local**	**Mean coverage per subcatchment**	
Livestock unit density	500kg livestock per km2	BFS GEOSTAT (2008), Landwirtschaftliche Betriebszählung / Census of farming, Bundesamt für Statistik, CH-2010 Neuchâtel and Corine Land Cover (CLC) 2018, Version 2020_20u1	2008	25ha (CLC); 2km catchment (BAFU)	NA	STARS reach contributing area	Mean	NA
Tree cover density	%	High Resolution Layer - Tree Cover Density (TCD) 2018	2018	10m	NA	Local	NA	NA
**Imperviousness density (urbanization)**	**%**	**High Resolution Layer - Imperviousness Density (IMD) 2018**	**2017-2019**	**10m**	**NA**	**Local**	**NA**	**NA**
Diffuse nitrogen inputs in waters	total loss (kg/ha*a)	BAFU	2004-2009	100m	Model reference year 2010	STARS reach contributing area	Mean	NA
Diffuse phosphorous inputs into water	total loss (kg/ha*a)	BAFU	2004-2009	100m	Model reference year 2010	STARS reach contributing area	Mean	NA
Insecticide application rate	treatments per % cropland	BFS GEOSTAT (2008), Landwirtschaftliche Betriebszählung / Census of farming, Bundesamt für Statistik, CH-2010 Neuchâtel	2008	Polygon of municipality	NA	STARS reach contributing area	Mean	NA
Wastewater concentration	m3/year	BAFU Gewässerabschnittsbasierte Einzugsgebietsgliederung der Schweiz GAB-EZGG-CH	2014	2km catchment	NA	STARS reach contributing area	Mean	log10
**Asymmetric colonization index (connectivity)**	**relative connectivity**	**derived from EU hydro data**	**NA**	**river reach**	**NA**	**BAFU subcatchment**	**NA**	**Log10+0.001**
**Distance to lake**	**Meters**	**derived from EU hydro data and BAFU barrier dataset**	**NA**	**river reach**	**NA**	**BAFU subcatchment**	**NA**	**Log10+1**

Data processing

We compiled data on the spatial distribution of 19 environmental variables representing bioclimate, flow regime, land use, habitat quality, water quality and river connectivity from publicly available sources. In addition to selected variables, we evaluated the spatial scale of effects on freshwater fish species distributions based on review, elicitation, and discussion amongst co-authors and processed data according to the expected scale of effect (Table 3). Depending on the variable and the dataset, we calculated variables at four potential spatial scales, i) the local values 100m buffered from river, ii) aggregated to "reach contributing area" derived from openSTARS, iii) aggregated to "catchment area" derived from openSTARS and iv) if data were originally provided aligned to Switzerland's Federal Office for the Environment geodata sub-catchments (~2km^2) they are aggregated to this product for consistency. We processed our data to account for the directionality and structure of river networks by using the openSTARS workflow, an open source implementation of STARS using R and GRASS GIS [12,13]. We first derived a topologically correct river network from the European Digital Elevation Model (v1.1) with a burn-in defined by the EU-Hydro River Network Database (v1.3) setting the accumulation threshold parameter to 1500 and burn-in depth of 50m. We used openSTARS to identify the adjacent land area of each segment that provides the lateral overland flow, known here the "reach contributing area". We also derived the full upstream "catchment area" of each river segment, representing a larger spatial scale than reach contributing areas. We calculated the mean, sum and/or proportion of area at these two spatial scales. In addition to the openSTARS workflow, we used spatial operations in R to buffer stream segments by 100m and calculated the mean, sum, or proportion of area of each relevant variable at a local scale. Finally, we aggregated data to the 2km sub-catchment scale if provided by the Swiss geodata portal (e.g., BAFU Ecomorphology-F dataset).

A fundamental aim of our work is explainability and interpretability of our model outputs, and collinearity induces challenges in interpreting the independence of variable effects and SHAP values. We used several strategies to limit the effects of multicollinearity in our modelling including both pre-processing variables and model selection. First, by investigating bi-plots and Spearman's rank correlations between variables we identified potentially confounding factors that would lead to misinterpretation of focal variable effects. Specifically, multiple covariates were correlated with elevation, discharge, slope and distance to lakes (e.g., river morphological modification index, mean surface imperviousness, proportion cropland cover, mean tree cover density, mean livestock unit density, mean diffuse nitrogen inputs, mean diffuse phosphorous inputs and mean insecticide application rates). Random forests do not recover independent effects of each variable, and as such, we used the residuals of a generalized additive model relating each potentially confounded covariate to all confounders. GAMs were fitted using the R package 'mgcv' (version 1.8-38)[14] with elevation, discharge, slope and distance to lakes modelled as penalized regression splines. For river morphological modification index and mean surface imperviousness variables, we used the residuals of these models as a predictor variable in the species distribution model. This pre-processing step changes the interpretation of these variables, now interpreted as the relative value of the variable given the site's elevation, discharge, slope and distance to lake. For the remaining 6 variables (livestock, nitrogen, phosphorous, insecticide, cropland, trees), potentially confounding effects could not be successfully removed. As such, we thought it more conservative to remove these variables from further assessment because our approach was dependent on having high confidence in the recovered response of species occurrence to environmental gradients, and we had difficulties interpreting these variable effects in a logical way.

From our final pre-selected set of variables, we then identified and used only those that were statistically supported using the BORUTA algorithm in the R package 'BORUTA' (version 7.0.0) [15]. This method was developed to provide a statistically valid approach to remove variables that do not sufficiently improve the fit of random forest models [15].

Transfer data

We did not transfer these models to new temporal domains.

Section 3: Model

Multicollinearity

Multicollinearity: We used several strategies to limit the effects of multicollinearity in our modelling. However, we also recognized the inherent uncertainty in correlative statistical approaches to predict spatial ecological patterns which cannot perfectly accommodate causal inference from observational data [16]. As such, 'removing multicollinearity' by removing variables can artificially decrease uncertainty [17]. We therefore favored simpler models that were interpretable over more predictive models that had poor links between biological mechanisms for how each environmental factor affects species' populations and thus habitat suitability predictions. As such, we aimed to include the most direct drivers possible, but note that some known to be important variables were not available at the broad spatial scale required for creating spatially continuous predictions from species distribution modelling, and so could not be included in our analyses (e.g., cover of in-stream vegetation, river-bed properties, fine-scale spatial and temporal flow dynamics). We also chose not to reduce the dimensionality of our variables because we want to interpret the relationships more directly between drivers and species occurrences.

We ranked an initially more comprehensive list of variables (n=31) by importance and identified the expected spatial scale of importance (n=3; local scale, reach contributing area, upstream catchment). We selected in our final set of variables the highest-ranking variables that also represented independent ecological gradients without redundancy in variables, leaving 18 variables for further consideration (e.g., we excluded coniferous and deciduous tree cover in favor of overall tree cover, we excluded the land use class 'pasture' in favor of a more quantitative livestock unit density, we removed elevation due to covariation with land use, connectivity and temperature).

We removed 6 of these selected variables which were strongly confounded with elevation, slope, river discharge or distance to lakes (see main manuscript). We removed these variables only when

confounding effects could not be removed by the two-step modelling approach described in the main manuscript.

In the modelling steps, after the variable pre-selection, we applied the BORUTA algorithm to automatically select relevant variables when using random forest models. This algorithm was specifically developed to find all relevant variables, instead of building a predictive black-box model [15]. The BORUTA algorithm iteratively adds "shadow features", which are randomized versions of original features, to random forests and removes real features with a lower Z-score than the maximum Z score of shadow features (full details in [15]). We retained all features identified as 'important' using BORUTA R package (version 7.0.0).

Model settings

Model settings (fitting): We fitted down-sampled random forests using the randomForest package in R (version 4.7-1.1). The number of trees was set to 1000. The number of parameters in each tree (mtry) was set as the default for classification problems (square-root of the number of variables). Most importantly, to fit down-sampled random forests, we set the 'sampsize' parameter to ensure both classes in the response data (0 and 1) had a sample size equal to lowest number of records in either class (as in [5]). Note that randomForests for SDMs are not automatically implemented this way in standard SDM software (e.g., [18]), but is easily completed using the instructions in [5].

Model estimates

Coefficients:

Random forests do not output coefficients, but we interpret variable importance and effect using Shapley values. [19,20]

Variable importance:

We calculated an indicator of global variable importance by calculating the mean absolute Shapley value per variable [19–21].

Variable effects:

We determined variable effects by plotting Shapley values against the original environmental values [19–21].

Analysis and Correction of non-independence

None

Threshold selection

For binary predictions, we selected the threshold that maximized the TSS (only used in Figure S5)

Section 4: Assessment

Performance statistics

We derived a set of metrics from contingency matrix containing true positives (TP), true negatives (TN), false positives (FP) and false negatives (FN):

- Overprediction rate = FP / TP + FP = proportion of all presence predictions that are incorrect.
- Underprediction rate = FN / TP + FN = proportion of all absence predictions that are incorrect.
- Sorensen index = (2TP) / (FN + 2TP + FP) = ignoring true negatives, what is the overlap between true and estimated classes.
- Jaccard index = TP / FN + TP + FP = ignoring true negatives, what is the ratio between true and estimated classes.
- Sensitivity = TP / TP + FN = proportion of all presence values that are correctly predicted

- Specificity = TN / TN + FP = proportion of all absence values that are correctly predicted
- True skill statistic = sensitivity + specificity – 1 = balance of sensitivity and specificity
- Matthew's correlation coefficient = $\frac{TP\ x\ TN - FP\ x\ FN}{\sqrt{(TP+FP)\ x\ (TP+FN)\ x\ (TN+FP)\ x\ (TN+FN)}}$ = contingency matrix version of Pearson's product moment correlation between predictions and observations.

In addition, we estimated the threshold-independent area under the receiver operative characteristic curve.

Plausibility check

Response shapes: We reviewed all response curves within the project team to ensure plausibility and refitted the species modelling pipeline removing variables that had implausible relationships. We did not remove variables that were against expectations but had theoretical foundations (i.e., while we expected human stressors to have, on average, a negative effect, we did not remove cases with positive effects of human stressors can occur through indirect predator release). Instead, we removed highly non-linear responses that have no theoretical foundations or plausible explanation.

Expert judgement: All range maps were reviewed by the project team against input data and expert knowledge.

Section 5: Prediction

Prediction output

Prediction unit: We interpret the predicted response value as an environmental suitability index, although it is built on presence-absence data. This is because of our down-sampling procedure balances the presences and absences within each tree of the random forest such that the model predictions do not represent probability of occurrence.

Uncertainty quantification

Scenario uncertainty: We did not quantify future scenarios.

Novel environments: We did not quantify future scenarios so no novel environments exist.

Supplementary Note 1

Technical details of Shapley values

The mathematical foundation of Shapley values was derived from coalition game theory attempting to decompose deviations in cooperative game scores into players contributions per game [22]. These insights have been applied to interpret statistical and machine learning models by attributing the contribution of a variable to the difference between a mean model prediction and the actual prediction for a particular observation in the model. This observational level explanation is the key insight provided by Shapley values. We provide a brief summary of the Shapley value, but for complete mathematical derivation of the approach used see [23] with [24] providing mathematical proofs, see also section 2.9 in [25] and see [26] for an accessible overview of concepts and available software and [21] for a recent application to soil organic carbon content and applications in ecology more generally [19,20], and note multiple applications outside of ecology (e.g., [27]). We summarise the information in these references here.

We are interested in isolating the effect of a single variable in a single location, which is the observation level in our model. We want to calculate how this variable causes a deviation in prediction for this observation from the mean prediction of our model. This concept is termed the situational importance. In the simpler case of an additive linear model, e.g.,

(1) $$f(x) = f(x_1, \ldots, x_n) = \beta_0 + \beta_1 x_1 + \cdots + \beta_n x_n,$$

the situational importance of $X_i = x_i$ is given by the expression

(2) $$\varphi_i(x) = \beta_i x_i - \beta_i \mathrm{E}[X_i].$$

However, in the linear model case, variables do not interact and so the contribution of x_i is the same across all observations, and as such the model is easy to interpret. This assumption of additivity is unrealistic in most ecological applications of phenomenological models because the model is often non-additive, can have unknown and unspecified functional form, and few assumptions are made on the potential effects of variables and their interactions. This is especially true in machine learning applications aiming to predict and explain ecological properties using spatial environmental variation, a domain where machine learning models have high predictive accuracy but often limited interpretability. In addition, model agnostic approaches to situational importance are beneficial where ensembles of models are used to make predictions, each with different statistical foundations and mathematical approach to model fitting.

To overcome these challenges, [23] developed a general approach to quantify situational importance, equivalent to Shapley values, for any model based on perturbation of subsets of variables and observing deviation of model outputs for a given observation. Because variables interact, every subset of possible variables must be considered when calculating situational importance of x_i. Equation (4 & 5) in [23] defines the model prediction conditional to only a subset of features values being known:

(3) $$f_Q(x) = E[f \mid X_i = x_i, \forall i \in Q],$$

where $Q \subseteq S = \{1, 2, \ldots, n\}$ is a subset of features. For any given combination of features ($f_{\{\}}(x)$), the contribution of a subset of feature values can be defined as the change in prediction caused by observing the values of a certain subset of features for a given observation:

(4) $$\triangle_Q (x) = f_Q(x) - f_{\{\}}(x).$$

After defining the contributions of interactions, see equations 6-8 in [23], the contribution of the variable to the observed prediction is explicitly defined as:

(5) $$\varphi_i(x) = \sum_{Q \subseteq S-i} W \cdot \left\{\triangle_{Q \cup \{i\}} (x) - \triangle_Q (x)\right\}$$

which calculates the marginal contribution of variable x to the prediction observation i as a weighted mean across all n^2 combinations of variable observations, exclusive of i. W is a weighting factor,

calculated to upweight the variable combinations that include more variables [23]. This formulation of interpreting observation-level variable contributions has benefits over other interpretable machine learning approaches, such as LIME, by satisfying the efficiency, symmetry, dummy and additivity properties [22,23], see [26] for further discussion.

To calculate the exact Shapley value is extremely computationally challenging because the number of possible coalitions increases exponentially with the number of variables, and predictions from all possible combinations of variables must be calculated with and without the focal feature. We used [23]'s Monte-Carlo approach to approximate Shapley values with 10,000 simulations using the R package '*fastshap'* [28]. As with any method, alternatives implementations exist, but for a highly useable package see also 'SHAP' (Shapley Additive exPlanations) by [29] and kernelshap (https://github.com/ModelOriented/kernelshap) provide additional overviews of the general approach and potential outputs.

Supplementary Note 2

Explainable AI for Species Distribution Models

Quantifying expected and shadow distributions using SHAP values

Abstract

Appendix to the manuscript Waldock et al (2024) article titled "Deconstructing the geography of human impacts on species' natural distribution". Here we outline the application of Explainable AI tools to species distributions models and show the code for how we derived the shadow distributions and expected distributions in Waldock et al (2024) with more detailed descriptions.

Background

Using SDMs in fundamental and applied ecology Fundamental questions in basic and applied ecology depend on understanding why species live in certain locations, and not in others. The environmental conditions of a location are an important determinant supporting or preventing species occurrence. Species distribution models (SDMs) combine the distribution of environmental conditions and the distribution of species occurrences through a statistical model with the aim of calculating maps of species distributions. Note that such spatial models can also use other available biological response variables such as presence-absence, abundance or population growth rate (Figure 1).

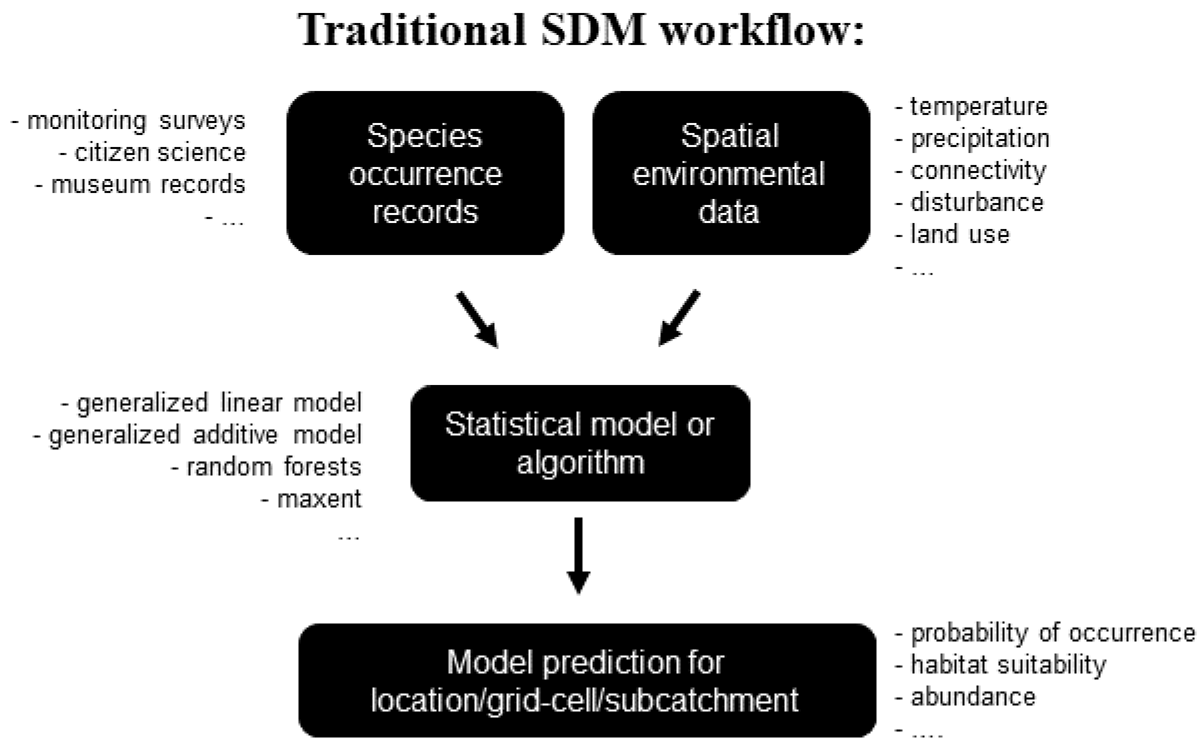

Figure 1. Broad overview of species distribution modelling frameworks

This workflow generates broad insights when assessing the overall model outputs and properties across all input observations in the model. This is referred to in the interpretable machine learning literature as investigating the *global model* scale, which commonly provides the following outputs:

- Mapping model predictions for unobserved areas to define a continuous map of the spatial variation in environmental suitability for a species.
- The overall importance of each variable for the models predictive accuracy e.g., permutation variable importance.
- The overall response of the species to each environmental factor.

Limitations of SDMs for fundamental and applied ecology Many ecological and conservation related questions demand a more detailed understanding of species distributions than the above scale of investigation can provide. For example, explaining why a population is expected to be present in a given location (with a high environmental suitability score) is very challenging. We simply know that a location has a high suitability, but it could be due to any of the environmental factors included in the model that have a positive effect on the species. Likewise, environmental managers often want to understand what would happen with a change in a given location. However, it is challenging to quantify why a species is expected to be present in a given location in the first instance, and therefore, it is hard to predict what an environmental manipulation might achieve and whether the manipulated factor is the main factor limiting a species locally.

We, therefore, lack knowledge on the importance of local ecological constraints and threat effects. Without this knowledge, it remains more difficult to effectively manage biodiversity. It would be useful to obtain localised insights from these broad scale models that highlight the influence of multiple co-occurring environmental factors on species distributions. This is the main challenge we attempt to provide one solution in Waldock et al. 2024.

To overcome this challenge and help explain why models make certain predictions, local explainable artificial intelligence approaches have been developed but have been rarely applied to ecological systems (but see 19, 20)). These approaches aim to provide explanations at the observation level of the model, elucidating why specific values for the variables in a model led to the prediction for that location. In our manuscript, we utilized SHAP values as they are model-agnostic and often yield more reliable estimates of local variable contributions compared with other methods (29).

For a readable overview of local interpretations from explainable artificial intelligence and machine learning, please refer to the Local interpretable models chapter in the book by Christoph Molnar. For a detailed mathematical overview of the SHAP approach we implement see (23), and also (27) for an alternative approach. Excellent tutorials for SHAP and kernelshap exist which provide overviews of the general approach and potential outputs. See also (21) for an application of SHAP values to ecosystem properties and (29) for medical applications.

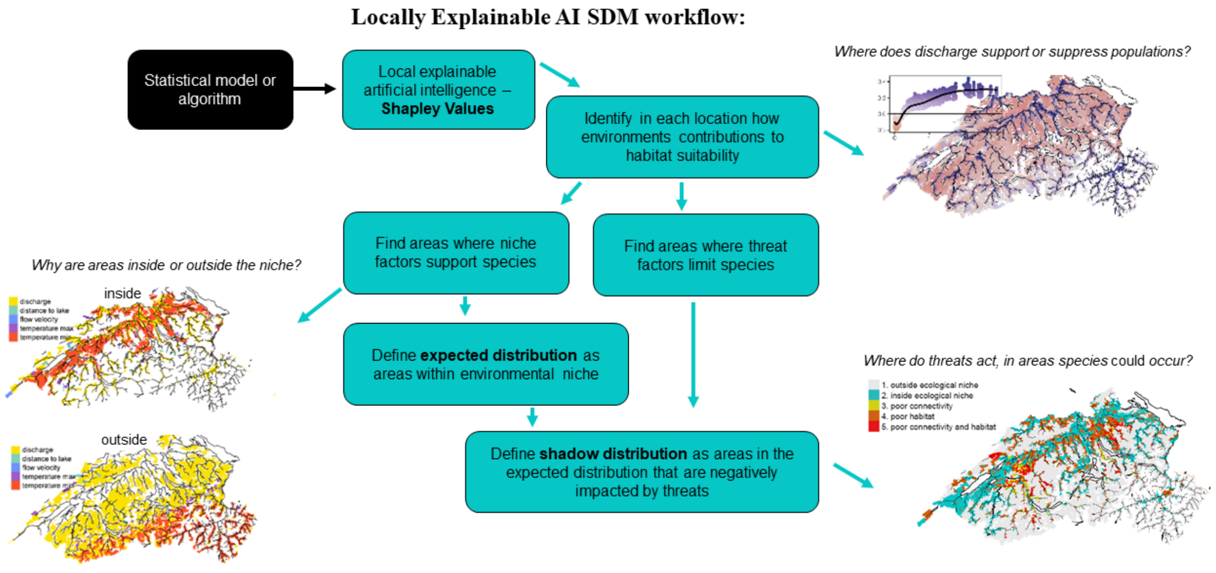

Figure 2. Overview of our application of explainable artificial i ntelligence t o b etter u nderstand d rivers of species distributions.

We demonstrate in the code below how to implement the workflow from Waldock et al 2024. We briefly generate a species distribution model but focus on the analysis after the SDM has been created. We show the software to estimate SHAP values and summarize, for each observation level in our work (a river subcatchment), how we generated SHAP values that provide insights into the local contribution of each environmental variable to the overall model prediction. We then separated our variables into those assumed to characterize the natural niche of the species and those characterizing the threats expected to impact a species distribution. We then defined two new distributional concepts that are, to our knowledge, not quantifiable through traditional species distribution model workflows. We quantified the ***"expected distribution"***, defined as the areas within the natural niche of the species. We then identified the areas where threats contributed nega-tively to species distributions within this expected distribution. We refer to this as the "shadow distribution," as this property measures areas where species are living in the shadow of human influences.

Section 1. Traditional species distribution modelling approach

We first briefly outline the main inputs to species distribution modelling and generate spatially continuous predictions of environmental suitability in unsampled locations. We then later apply SHAP value analysis to the fitted species distribution model. As in our manuscript, we focus on the species Alburnoides bipunctatus shown below because it is a relatively widespread species in our catchments and is classified as 'Vulnerable' based on apparent population reduction and decline in the area and quality of habitat.

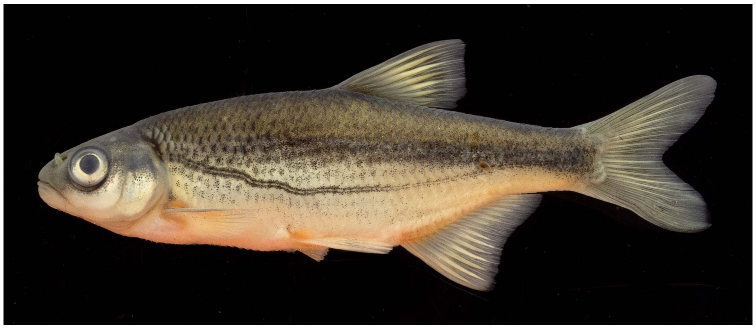

Load packages We first need to load the various packages used in this script. For full documentation of the package version used see the session info at the end of the document.

```
## LOAD PACKAGES

# local in pacman
if(!"pacman" %in% rownames(installed.packages())){install.packages("pacman")}
library(pacman)

# load in packages - version of packages used are displayed at the end of the workflow
p_load(tidyverse, terra, tmap, sf, randomForest, Boruta)
```

```
## LOAD OCCURRENCE AND ENVIRONMENTAL DATA

# occurrence dataset
pa <- st_read("data/data example/pa.shp", quiet  = T)

# environmental dataset
env_data <- rast("data/data example/env_data.tif")

# complete combined dataset
full_data <- readRDS(file = "data/data example/sp_example.rds")

# load pre-made spatial objects for plotting
load(file = "data/data example/all_spatial_Waldock2023.RData")
```

1a. Species occurrence records The response variable in our modelling framework is the presence (1) or absence (0) of a species from a location. Our dataset has been compiled from electrofishing surveys where all species recovered from streams were identified. The SHAP value analysis, calculation of local variable contributions and the shadow distribution framework could also be applied to other types of data on ecological and biological responses to environmental gradients (such as presence-only, abundance, body size, trait properties, demographic rates etc).

```
## [1] "number of records = 3229"

## [1] "number of presence = 85"

## [1] "number of absence = 3144"
```

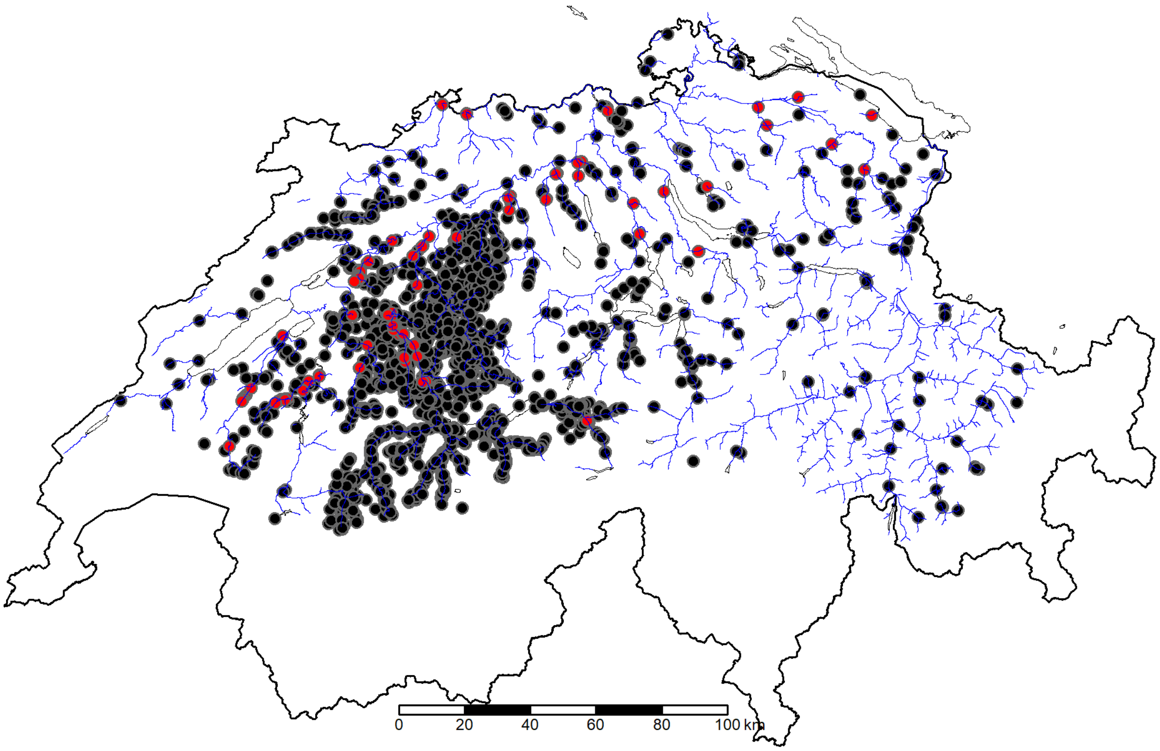

Figure 3. Spatial distribution of presence (red) and absence (black) points across Aare-Rhine riverscape.

1b. Spatial environmental data The second component of a species distribution model is the spatial environmental data. In our work, we compile freshwater specific variables for Switzerland that were available across the Aare catchment. However, there are many available data layers that have been used for SDM modelling purposes. This data has two purposes for SDM modelling: i) to provide explanatory variables for building relationships between occurrence and environmental gradients, ii) to provide spatially continuous predictions of occurrence probability or environmental suitability using the environmental values across full domain of interest (i.e., beyond the sampling locations). The environmental data are also important to the Shapley estimation as an input to estimate the local contribution of each environmental variable to the predicted suitability value.

Full name	Short name	Natural or threat
Monthly minimum temperature across 1981-2010	Minimum temperature	Natural
Monthly maximum temperature across years from 1981-2010	Maximum temperature	Natural
Maximum discharge in a subcatchment across years 1981-2000	Discharge	Natural
Minimum slope of river reach in subcatchment	Slope	Natural
Flow velocity of river reachs weighted-averaged within subcatchment	Flow velocity	Natural
Distance to lake	Distance to lake	Natural
River morphological modification (BAFU-Ecomophology F) weighted-average per subcatchment	Morphological modification	Threat
Floodplain proportion cover in subcatchment	Floodplains	Threat
Wetland proportion cover in subcatchment	Wetland	Threat
Imperviousness density COPERNICUS high resolution layer	Urbanisation	Threat
Asymmetric colonization index of river reach	Connectivity	Threat

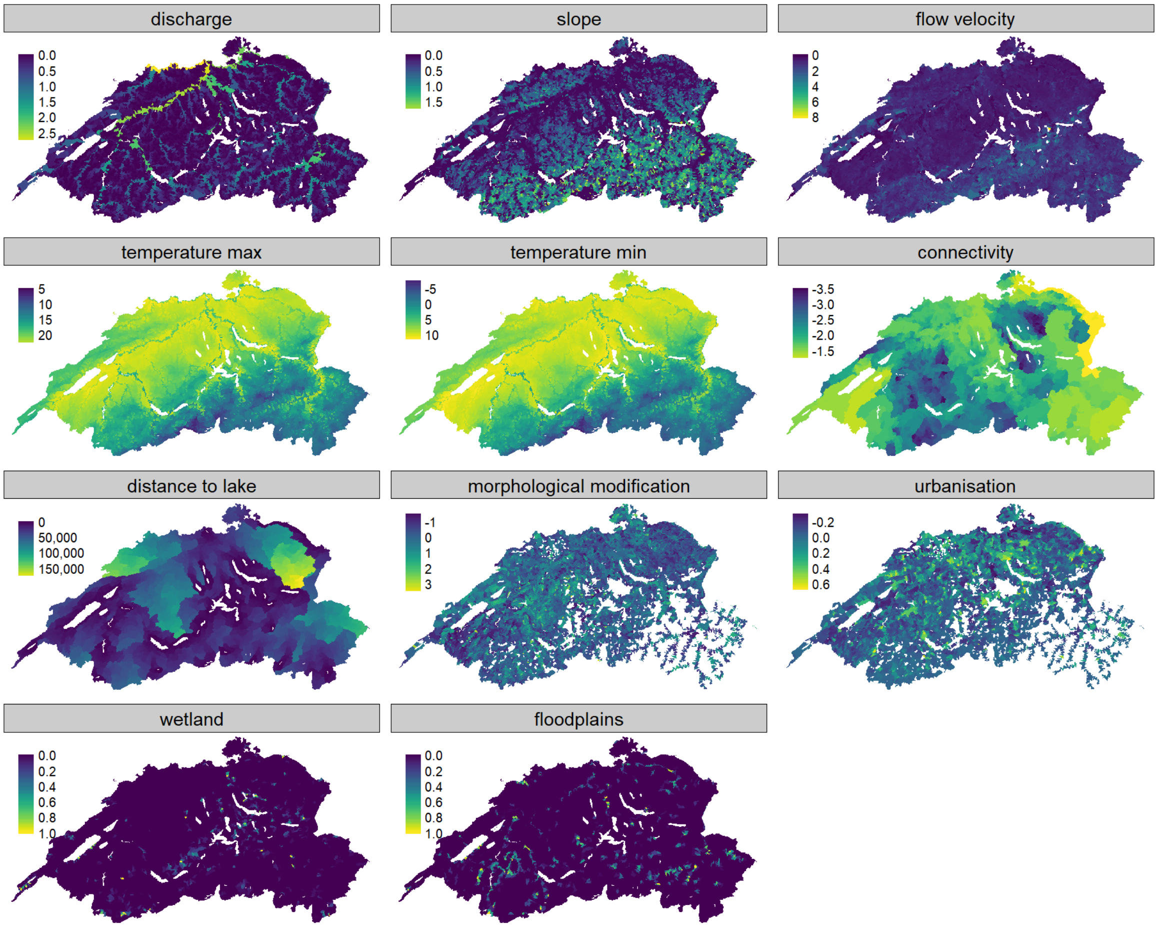

Figure 4. Map of the spatial environmental variables used to fit the species distribution model

1c. Species distribution model Here we used down-sampled random forests as in (5, 6) which provides good model performance when there are many more absences compared with presences. The explainable AI approach used here, SHAP values implemented in "fastshap," is agnostic to the exact underlying model. This is a major benefit as it could be applied to the commonly used ensemble modelling approaches e.g., (18). In the below script, we apply a custom wrapper for fitting down-sampled random forests using the approach in (5) and using the "boruta" method for selecting variables (15).

```
### RUN RANDOM FOREST MODEL

# Source functions that are wrappers to run random forests
# and variable selection available in this GitHub repository
funs <- lapply(list.files("data/data example/functions", full.names = T),
               function(x) source(x, echo = F))

# View data containing species occurrences and the relevant covariates
str(full_data, 2)
```

```
## 'data.frame':    3184 obs. of  14 variables:
##  $ occ                          : Factor w/ 2 levels "0","1": 2 1 1 1 1 1 1 1 1 1 ...
##  $ X                            : num  4141621 4121533 4159747 4123049 4119849 ...
##  $ Y                            : num  2718789 2670654 2717452 2635659 2642558 ...
##  $ ecoF_discharge_max_log10     : num  1.2977 0.0719 0.1931 0.0719 0.5289 ...
##  $ ecoF_slope_min_log10         : num  0.121 0.1404 0.0414 0.3964 0.0414 ...
##  $ ecoF_flow_velocity_mean      : num  1.606 0.778 1.046 0.494 0.589 ...
##  $ stars_t_mx_m_c               : num  21.2 23.1 22.9 19.3 20.2 ...
##  $ stars_t_mn_m_c               : num  8.25 9.9 9.59 6.27 7.16 ...
##  $ local_asym_cl_log10          : num  -1.78 -2.26 -1.69 -2.46 -2.46 ...
##  $ local_dis2lake               : num  83140 16145 52609 32454 23181 ...
##  $ ecoF_eco_mean_ele_residual   : num  0.99 -0.97 -0.342 -0.418 -0.618 ...
##  $ local_imd_log10_ele_residual: num  0.261 -0.102 0.049 -0.093 -0.199 ...
##  $ local_wet                    : num  0 0 0 0 0 ...
##  $ local_flood                  : num  0 0 0 0.303 0.485 ...
##  - attr(*, "na.action")= 'omit' Named int [1:45] 3 18 19 22 24 29 84 91 99 118 ...
##   ..- attr(*, "names")= chr [1:45] "3" "18" "19" "22" ...
```

```
# Generate species distribution model
var_selection_method = "boruta"
pa_rf_final <- rf_wrapper(full_data)
pa_rf_final
```

```
##
## Call:
##  randomForest(formula = occ ~ ., data = x[c("occ", rf_vars)],      ntree = 1000, sampsize = spsize, 
##                Type of random forest: classification
##                      Number of trees: 1000
## No. of variables tried at each split: 3
##
##         OOB estimate of  error rate: 9.39%
## Confusion matrix:
##      0   1 class.error
## 0 2814 286  0.09225806
## 1   13  71  0.15476190
```

```
##
## Call:
## roc.default(response = full_data$occ, predictor = predictions)
##
## Data: predictions in 3100 controls (full_data$occ 0) < 84 cases (full_data$occ 1).
## Area under the curve: 0.9912
```

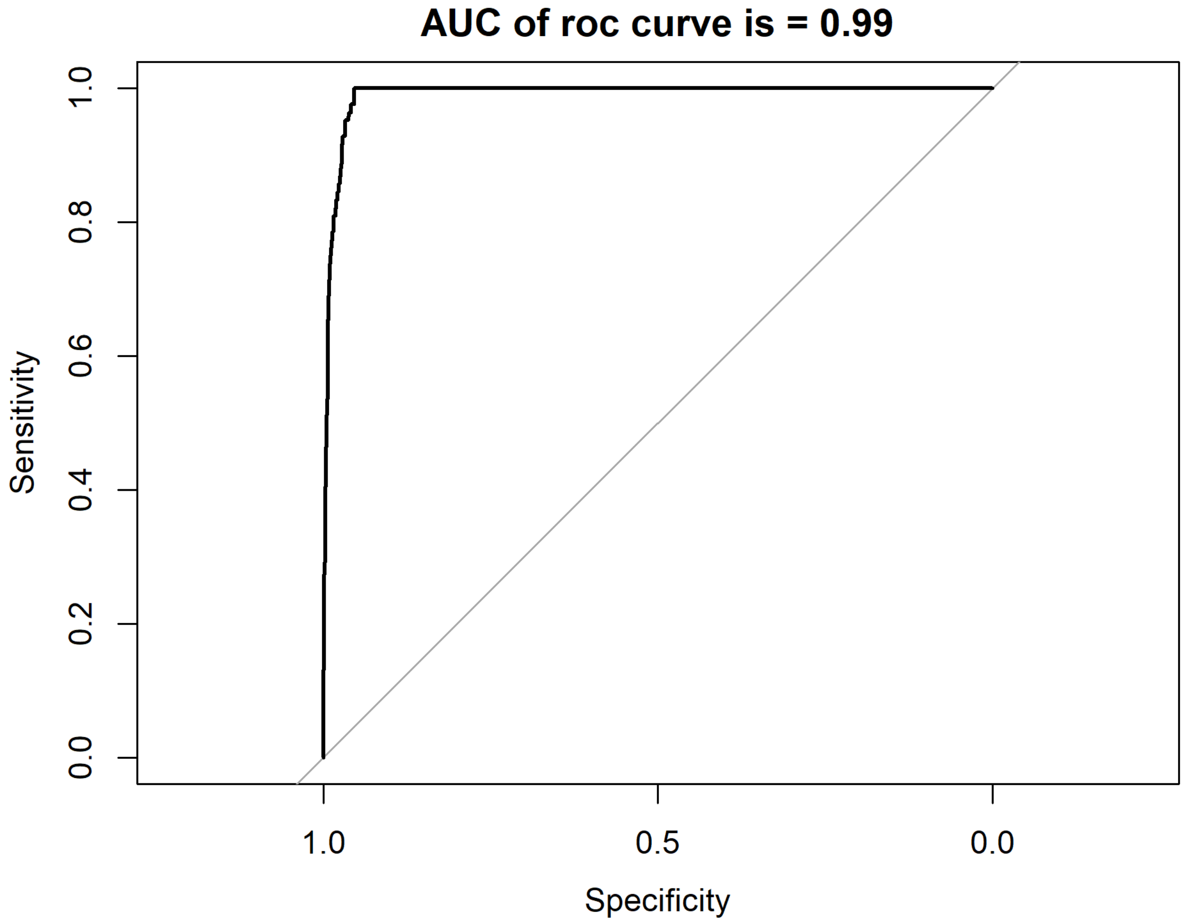

Figure 5. ROC curve of random forest SDM indicating within-sample performance

We do not aim to provide an in-depth overview of fitting and evaluating species distribution models and assume users are familiar with the biases, choices, and pitfalls involved (e.g. 1, 2, 3). In our manuscript, we use spatially-blocked cross validations and evaluate multiple metrics of model performance to assess model adequacy. However, our focus here is to demonstrate how to apply Shapley value analysis to SDMs in order to derive local contributions to environmental suitability scores and then quantify species shadow distributions.

1d. Environmental suitability predictions A key output of species distribution modelling is a spatially continuous map of the modelled response variable (e.g., occurrence, abundance). The name of this variable depends on the structure of the data and model, but is often called the "environmental suitability," "environmental suitability," or "probability of occurrence." Here, we used "environmental suitability"

```
### MAKE MODEL PREDICTIONS OF ENVIRONMENTAL SUITABILITY

# generate threshold using ecospat based on TSS
thresh <- ecospat::ecospat.max.tss(as.numeric(predict(pa_rf_final,
                                                      type = "prob")[,2]),
                                   as.numeric(pa_rf_final$y)-1)

# make prediction of environmental suitability from random forest model
habitat_suitability <- terra::predict(env_data, pa_rf_final,
                                      type = "prob")[[2]]
threshold_suitability <- as.numeric(habitat_suitability > thresh$max.threshold)

# extract the value of suitability per subcatchments (TEILEZGNR)
suit_sp <- terra::extract(habitat_suitability,
                          terra::vect(subcatchments_final),
                          fun = function(x) mean(x, na.rm = T),
                          touches = T)

# rename
suit_sp <- suit_sp %>%
  cbind(., subcatchments_final %>% select(TEILEZGNR)) %>%
  rename(., c("suitability" = "X1"))
```

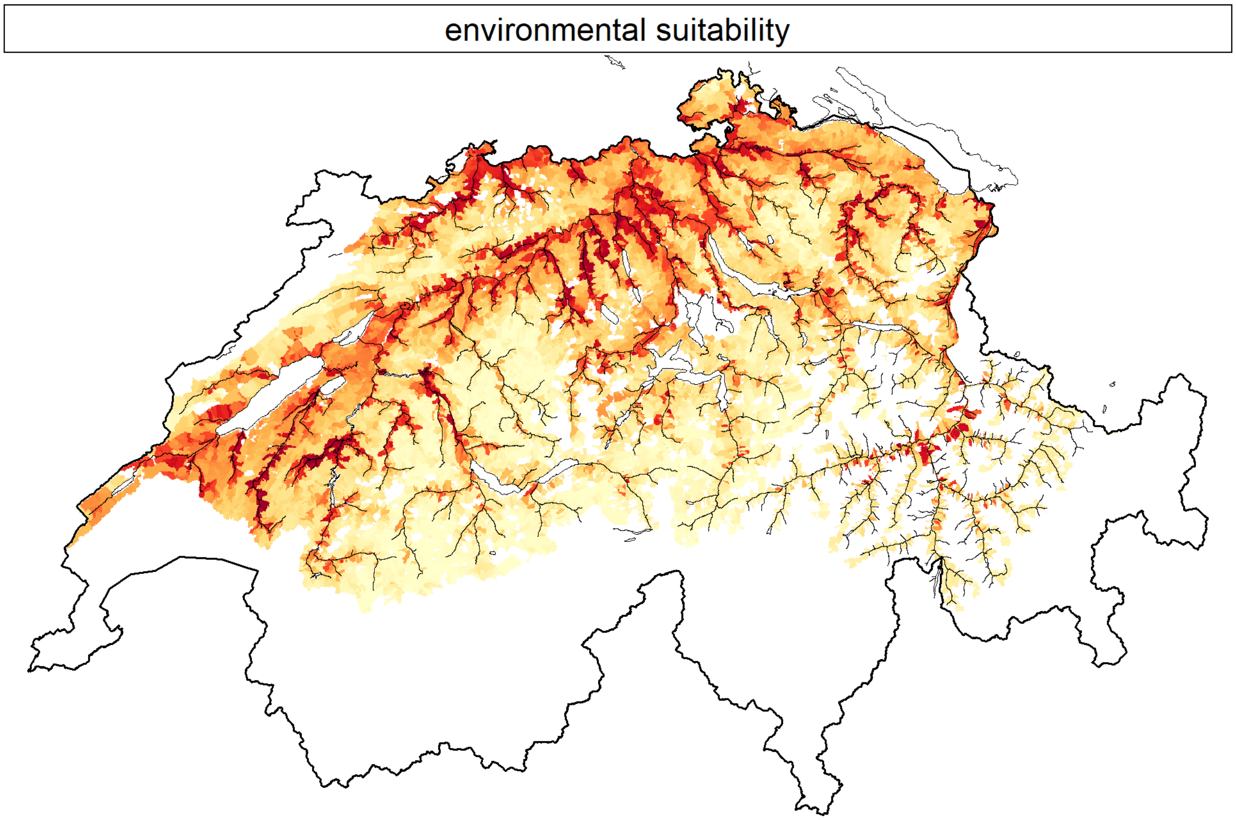

Figure 6. Environmental suitability prediction as the predicted values from our fitted species distribution model

One of the main limitations with the insight gained from a map of environmental suitability scores is that we do not know why the environmental suitability is high or low in particular areas. We only have a measure of how potentially suitable the habitat is in a specific location For many fundamental and applied questions it would be important to determine what are the specific environmental factors that contribute to a location being suitable or unsuitable. For example, under climate change predictions, it would be important to know that at range edges species are actually limited by fast flow velocities in rivers but not temperature. Or alternatively, before restoring a river, it would be important to know that low habitat complexity is an important factor leading to low environmental suitability, rather than cold water temperatures limiting the species locally.

Section 2. Shapley analysis of a Species Distribution Model

Here we calculate the SHAP values for all subcatchments based on the subcatchments environmental values and the random forest models. We aim to explain why the model makes a prediction of environmental suitability for a given subcatchment given the specific set of environmental conditions in that particular subcatchment.

2a. Calculate Shapley values of the random forest model Before running the SHAP analysis we need to set up some technicalities. We must load the fastshap package (28) and define the prediction function used by the model. In addition, we must create a vector of all features/covariates names in the model.

```
### PREAMBLE TO SET UP SHAPLEY ANALYSIS

# load in the fast shap package used to calculate shapley values
p_load(fastshap)

# define the prediction function to use in fastshap
pfun <- function(object, newdata) {
 as.numeric(as.character(predict(object, newdata = newdata,
                                  type = "prob")[,2]))
}

# get the variables in a way that is model specific
vars <- colnames(attr(pa_rf_final$terms, "factors"))
```

We use the function `fastshap::explain()` in the fastshap package to run the Shapley analysis. This function takes the model object, the names of the features to explain, the data used to fit the model, new data to predict Shapley values, the prediction function to use, and the number of simulations to generate to create average Shapley values. In our main manuscript, we set the number of simulations to 10,000 and this number should be set as high as computationally possible to obtain the most accurate Shapley values. One simulation is the number of Monte Carlo repetitions used to generate the random coalition of variables used to generate the local contribution of any given variable as explained in the helpfile `?fastshap::explain` and in (29).

```
### RUN SHAPLEY VALUE ANALYSIS

# get the shapley values
shapley_pa <- fastshap::explain(

  # model object
  object = pa_rf_final,

  # names of features to explain
  feature_names = vars,

  # X data used to fit the model
  X = full_data[vars],

  # new data to predict on
  newdata = all_env_subcatchments[vars],

  # predictive function
```

```
  pred_wrapper = pfun,

  # number of replicates
  nsim = 100 # here should be set to as high as possible.
  # In the manuscript we used 10,000 taking a couple of days to run each time.

  )
```

The output of the Shapley analysis has the same column names as the covariates put in. To make it easier to process this data, we append the Shapley columns with "_SHAP" to differentiate from the environmental data values.

We create a combined spatial dataset with the subcatchments (TEILEZGNR), suitability scores from the random forests, the raw environmental data values, and the Shapley values.

```
### ORGANISE OUTPUT OF SHAPLEY ANALYSIS

# convert to a dataframe
shapley_pa <- shapley_pa %>% data.frame

# rename shapley data so it doesn't have the same names as the variables
names(shapley_pa) <- paste0(names(shapley_pa), "_SHAP")

# bind back in with the subcatchment environmental values
sp_shapley_pa <- cbind(all_env_subcatchments[c("TEILEZGNR", vars)],
                       shapley_pa)

# join in the spatial subcatchments in the Aare river catchment
# with the env. values and the shapley values.
shap_final <- left_join(left_join(subcatchments_final["TEILEZGNR"],
                                  suit_sp),
                        sp_shapley_pa)
```

Here we now have a dataframe that provides for each subcatchment the value of the environment, the modelled environmental suitability, and the effect of the subcatchment environment on the environmental suitability score (the Shapley value).

Section 3. Insights gained from Shapley values

Here we generate multiple summaries based on Shapley values including:

- 3a Variable contribution importance
- 3b Variable contribution direction
- 3c Species response curves
- 3d Spatial distribution of variable contributions
- 3e Relative contribution of variables in one location

3a. Average Shapley value across all subcatchments (variable contribution importance) The average absolute value of Shapley values gives an estimate of the overall variable importance in the model. For each subcatchment the Shapley value is turned to an absolute value so that the direction of the contribution (positive or negative) is ignored when calculating the overall importance. A variable has a low overall importance when most of the Shapley values have a low value. This is because this variable is not contributing significantly to a change in the environmental suitability score on average, and is therefore interpreted as globally unimportant. Note that, this global interpretation does not exclude that in some local subcatchments a variable that is unimportant on average can have a very strong local effect.

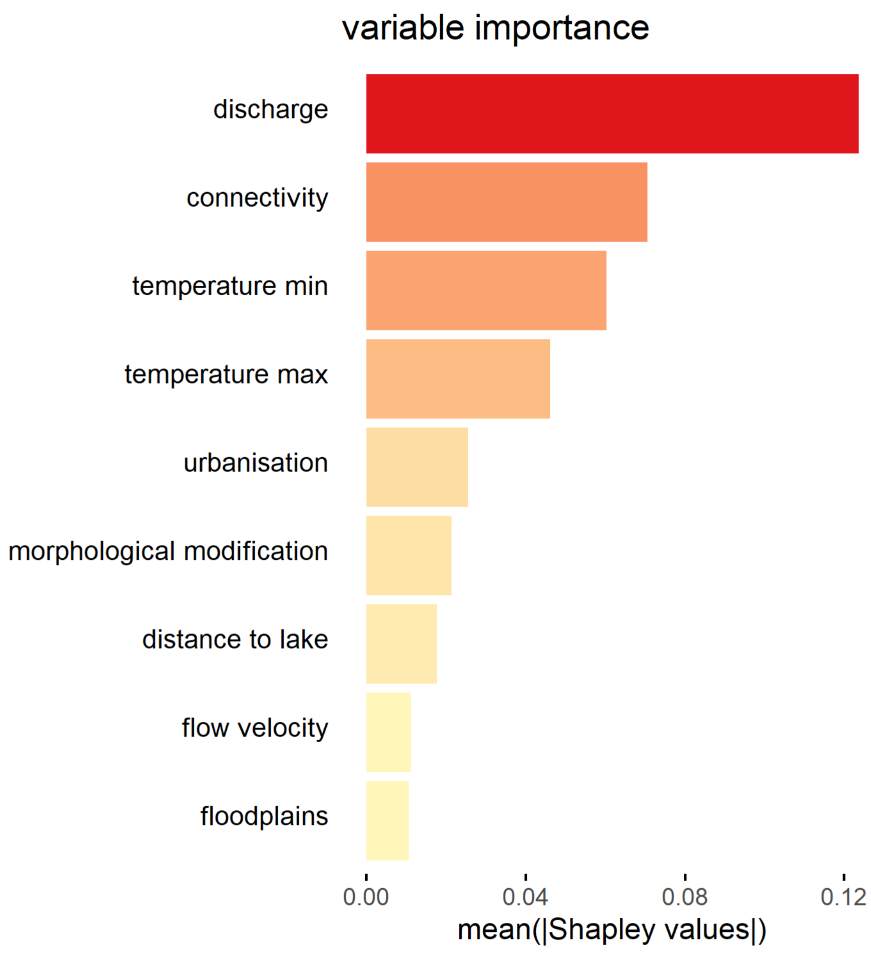

Figure 7. Shapley based variable importance scores

3b. Variation in Shapley values per environmental gradient (variable contribution direction) We can also show the distribution of the Shapley values per variable which shows if the overall effect of the variable is generally positive or negative. This approach does not show the relationship between the variables but instead the general impression of negative or positive contributions. For example, in the below plot we see that discharge contributes negatively in most subcatchments to environmental suitability. In contrast, temperature variables tend to have a more balanced distribution of effects across all subcatchments, some positive and some negative.

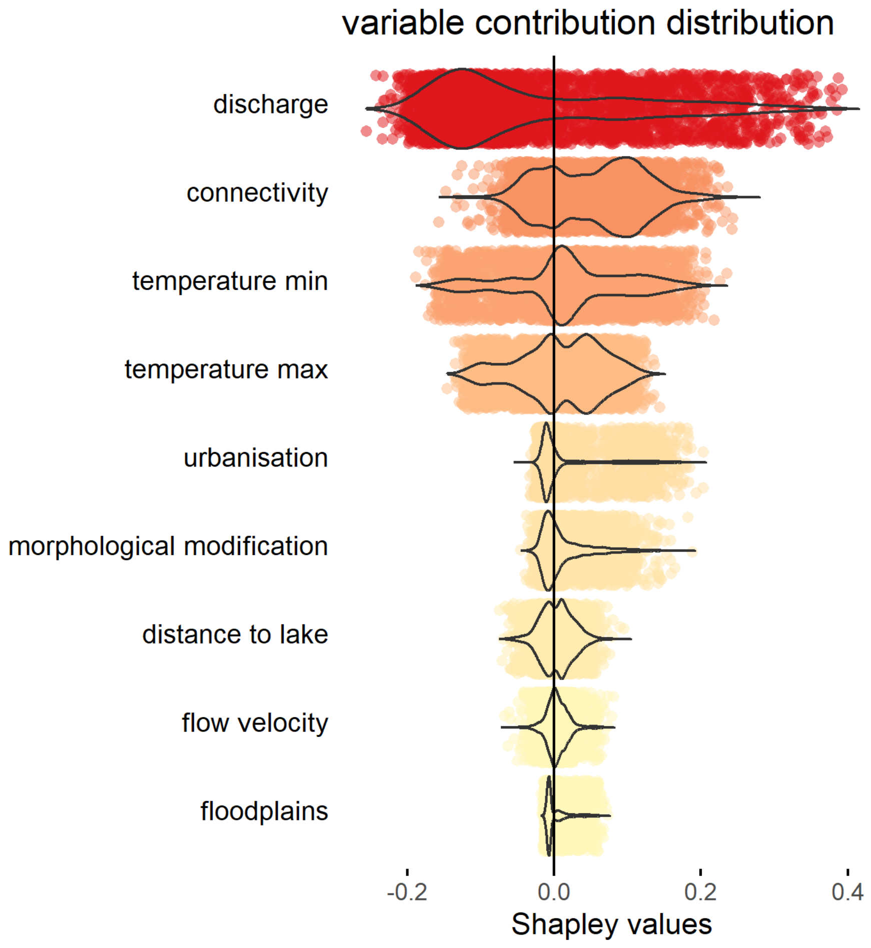

Figure 8. Shapley based distribution of variable contribution scores

3c. Species responses to environmental gradients (response curves) The Shapley value at a given environmental value indicates the contribution to the overall suitability, as summarised in 3a and 3b. We can also look at whether a variable has a positive or negative effect on environmental suitability using Shapley values (i.e., species response to environmental gradients).

When a variable has a positive overall effect on environmental suitability this indicates higher Shapley values at higher values of the environmental gradient (and vice versa). In this way, Shapley values plotted against environmental values indicate response curves much like other techniques for plotting these curves (e.g., predictions from models over all values of environmental gradients, Accumulated Local Effect plots).

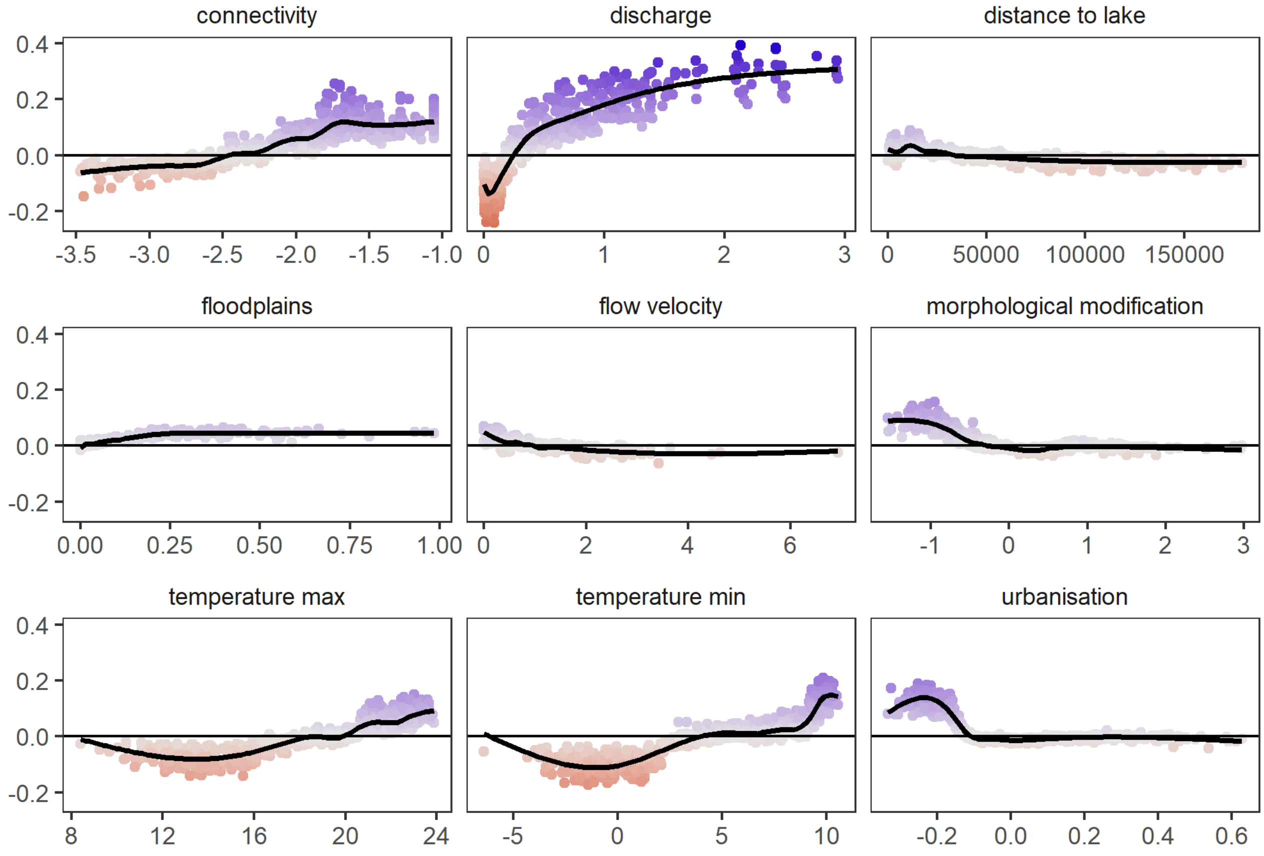

Figure 9. Shapley based response curves

3d. Spatial distribution of Shapley contributions to environmental suitability Given that we estimate the Shapley value per subcatchment, and we have spatial information on the subcatchments across the whole landscape, we can then predict the spatial distribution of the effect of a given variable on the environmental suitability. This can be interpreted as the *spatial distribution of species sensitivity* to each environmental variable.

This insight can be used to generate fundamental knowledge on the main ecological niche constraints on species distributions across a landscape. If used in more applied domains, we can see the main areas that are impacted by each threat which could help inform conservation decision making to find locations where species populations respond negatively to threats.

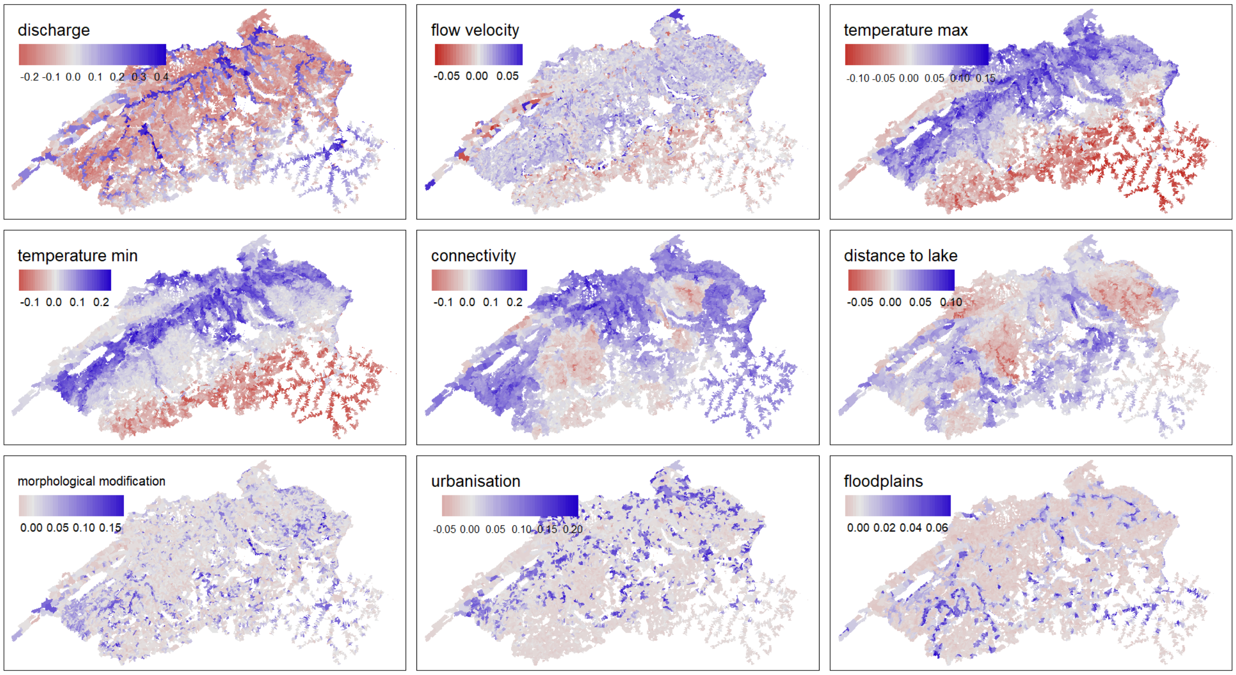

Figure 10. Map of Shapley values across Aare-Rhine subcatchments

3e. Relative contribution to environmental suitability in specific locations We demonstrate how Shapley values can be summarised to identify the main environmental constraints on species distributions in a specific sub-catchment. We first must identify the subcatchments of interest, which here we choose the Sense river in Switzerland which has a relatively high degree of connectivity, natural and largely unmodified flow regime. We extracted the Shapley values for all variables for all subcatchments that fall in the Sense drainage and took the mean Shapley value across subcatchments.

First we identify the subcatchments that fall in the Sense river:

```
#### ESTIMATE SHAPLEY VALUE BASED ON SINGLE CATCHMENT

# take one subcatchment in the sense river
test_subcatchment <- 79104

# get the shapley values
shapley_sense <- fastshap::explain(

  # model object
  object = pa_rf_final,
  # names of features to explain
  feature_names = vars,
  # X data used to fit the model
  X = full_data[vars],
  # new data to predict on
  newdata = all_env_subcatchments %>%
    filter(TEILEZGNR == test_subcatchment) %>%
    select(vars),
  # predictive function
  pred_wrapper = pfun,
  # number of replicates
  nsim = 10000, # here should be set to as high as possible.
  # In the manuscript we used 10,000 taking a couple of days to run each time.
  )

shap_sense <- pivot_longer(data = data.frame(shapley_sense),
                           cols = vars)
```

```
#### SHOW SECOND METHOD FOR CALCULATING SHAPLEY VALUES (GIVES IDENTICAL RESULT)

# other approaches are available to calculate shapely value
# explanations, but here appear to give the same result.
library(kernelshap)
SHAP <- kernelshap(object = pa_rf_final,
          X = all_env_subcatchments %>%
            filter(TEILEZGNR == test_subcatchment) %>%
            select(vars),
          bg_X = full_data[vars],
          pred_fun = pfun,
          exact = T)
SHAP_df <- SHAP$S
SHAP_df <- data.frame(SHAP_df)
```

Next, we estimate the mean predicted environmental suitability which is our baseline on which the Shapley values modify environmental suitability in the Sense river:

```r
#### CALCULATE MEAN MODEL PREDICTION ACROSS ALL SUBCATCHMENTS

# create baseline as the mean prediction of the model output
baseline_prediction <- mean(as.numeric(predict(pa_rf_final))-1)
baseline_prediction
```

```
## [1] 0.1121231
```

Next, we estimate the mean predicted environmental suitability of the Sense catchment. Once the Shapley values are summed, this is what the estimated environmental suitability converges.

```r
#### CALCULATE LOCAL ENVIRONMENTAL SUITABILITY

sense_prediction <- mean(shap_final %>%
                            filter(TEILEZGNR == test_subcatchment) %>%
                            pull(suitability), na.rm = T)
sense_prediction
```

```
## [1] 0.6388616
```

We see clearly that the average prediction for the Sense is much higher than the average suitability across all other locations in the Aare and Rhine catchments. A critical question is, why is this habitat better for this species?

We can investigate this question by looking at the model predicted deviation from the mean suitability. This visualises the contribution of each variable to the environmental suitability in the Sense, given this rivers particular environmental conditions, which helps explain why the suitability score is predicted from the model.

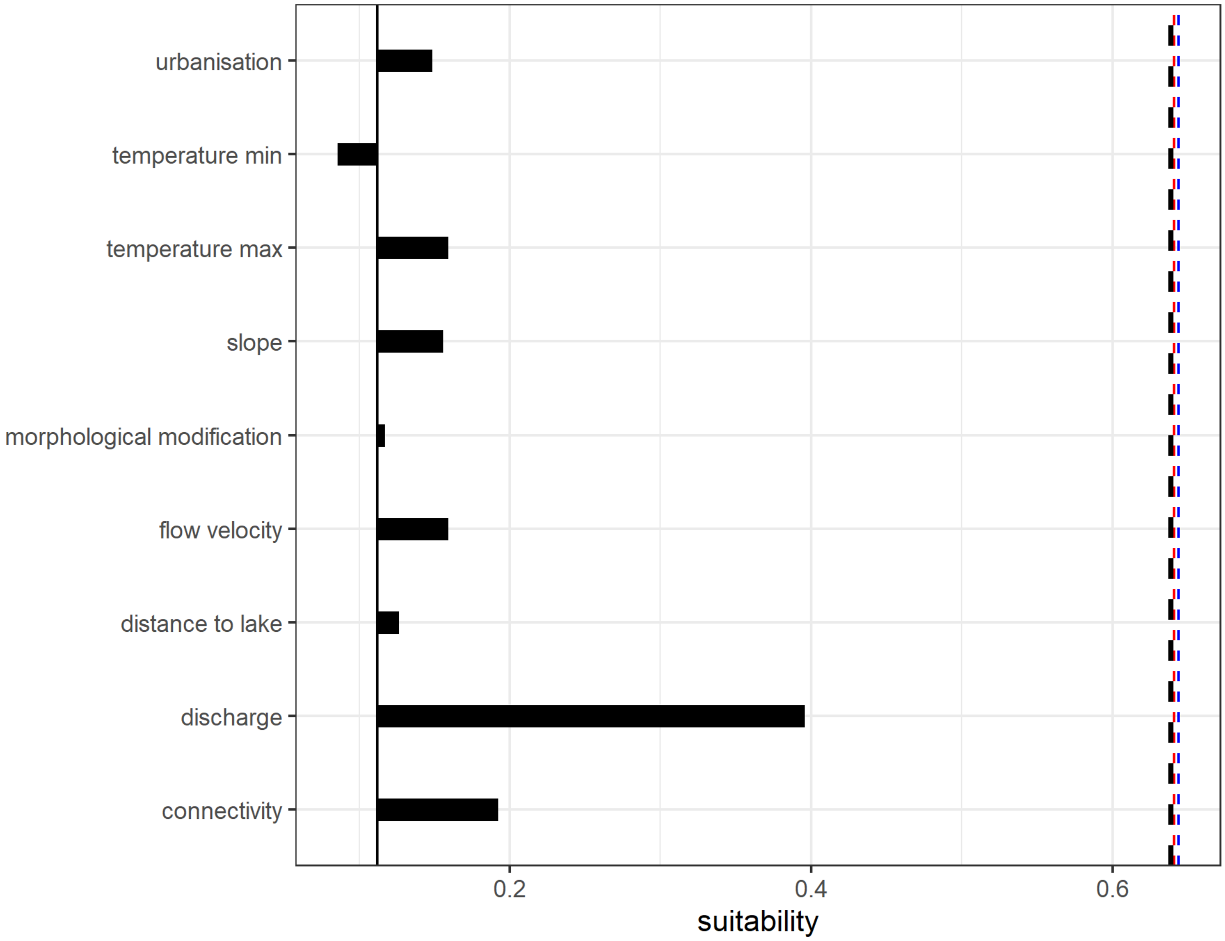

Figure 11. Shapley contributions to model local prediction (dashed lines) expressed as deviation from baseline model prediction. Red and blue dashed lines indicate the summed Shapley values + baseline prediction for fastshap (blue) and SHAP (red) methods, giving almost identical results.

The plot above indicates how each variable contributes to the deviation from the overall baseline prediction (0.11) to obtain the predicted suitability for this location (0.64). By estimating local explanations, we can understand why we obtained this prediction. The Shapley values highlight which variables at this location contributed to the difference between the baseline average prediction and the local suitability prediction. We observe that discharge and connectivity made positive contributions to the local prediction, along with urbanization, flow velocity, and slope, all contributing positively as well. Additionally, we notice a few negative contributions from habitat-related threats, indicating that these factors are unlikely to be limiting species occurrence in these locations. In our manuscript, we contrast two river systems with different ecological and anthropic conditions and their impact on species occurrence locally.

Section 4: Shadow distributions

We next demonstrate the calculation of shadow distributions based on Waldock et al. (2024). We introduce the concept of a shadow distribution as the areas where a species would be expected to occur but where threats negatively affected the species. We called this the "shadow" distribution to reflect that species are in the shadow of human influences.

Two types of shadow distributions We define two types of shadow distributions: the *"binary shadow distribution"* and the *"quantitative shadow distribution"*, described in more detail below. The key components to quantify each shadow distribution are:

i) a set of variables that define the ecological niche of the species, and

ii) a set of variables that define threats to a species.

In any given system, the factors defining the niche and the threats to species must be informed by well-grounded ecological theory and expert knowledge within the specific system.

Defining expected distributions from abiotic niche factors The ecological niche is defined by the relevant abiotic or biotic environmental factors that are expected to naturally determine individual fitness and population performance. The geographic space falling inside the ecological niche is defined as the *expected distribution.* The actual or realized distribution may deviate from this expected distribution due to human impacts within the species' distribution. The threat factors quantify human-related changes to the environment that are expected to reduce individual fitness and population performance, although it's important to note that for some species, threat effects may be positive.

In more specific terms shadow distribution is region within the expected distribution (defined by the natural niche) where human impacts negatively affect species. This property is expected to deviate strongly from traditional predictions from species distribution models (i.e., environmental suitability) if threat effects are important determinants of environmental suitability. This deviation occurs because the environmental suitability score combines the effects of all variables on a model prediction, including threats that reduce suitability in areas that would otherwise be suitable based on ecological niche factors alone. In contrast, we utilize Shapley values to calculate the contribution of each variable to the prediction. This separation of variable effects enables us to quantify the negative contribution of threats within areas where natural niche factors positively contribute to model predictions.

We will now outline how we calculate the expected distribution, as well as the species' binary shadow distribution and quantitative shadow distribution separately.

4a. Expected distribution to define baselines Before calculating the shadow distribution, we must quantify the expected distribution, and do so using binary and quantitative representations. The binary expected distribution refers to whether a location is inside or outside abiotic niche of species. The quantitative expected distribution is the environmental suitability of the areas inside the niche.

First we must define the natural niche factors. These are factors that we expect to define the natural conditions that constrain a species distribution, and therefore help define the realized ecological niche of the species. In many respects, this definition is somewhat subjective and should reflect your expertise on the focal species in addition to how well the variables represent the key non-human related ecological processes constraining a species distribution. We also opted to include habitat variables, such as the presence of "nationally important floodplains," as a threat in our exercise. This decision was taken because most subcatchments are not inside this category so the absence of floodplains can be perceived as having a negative impact on biodiversity.

```r
#### DEFINE THE NATURAL NICHE FACTORS (THAT ARE USED IN THE MODELS)

# define natural niche factors
natural_niche_factors = c("ecoF_discharge_max_log10_SHAP",  # discharge
                          "stars_t_mn_m_c_SHAP", # minimum temperature
                          "stars_t_mx_m_c_SHAP", # maximum temperature
                          "ecoF_flow_velocity_mean_SHAP", # flow velocity
                          "local_dis2lake_SHAP",  # distance to lake
                          "ecoF_slope_min_log10_SHAP") # slope

# subset the natural niche shapley values relevant to the specific species model
natural_niche_factors <- natural_niche_factors[natural_niche_factors %in%
                                                 names(shap_final)]
```

Here we next define areas as inside or outside of the natural niche of the species. We define subcatchmets as falling inside the natural niche of the species if the ***sum of the natural niche variable Shapley values is > 0.***

```r
#### DEFINE BINARY EXPECTED DISTRIBUTION

# is the sum of the natural niche variable Shapely values positive?
shap_final$natural_niche <-
  rowSums(st_drop_geometry(shap_final[,natural_niche_factors])) > 0

# what is the sum of the natural niche variable Shapley values?
shap_final$natural_niche_value <-
  rowSums(st_drop_geometry(shap_final[,natural_niche_factors]), na.rm = T)
```

We ask whether what proportion of subcatchments is the sum of natural niche factor Shapley values positive (i.e. > 0). This highlights a simple summary that around `round(sum(shap_final$natural_niche==0, na.rm = T) / sum(!is.na(shap_final$natural_niche), na.rm = T)*100)` of subcatchments are outside of the ecological niche of the species.

```r
#### MAKE TABLE OF INSIDE OR OUTSIDE OF NICHE

signif(table(shap_final$natural_niche) / nrow(na.omit(shap_final)), 3)
```

```
##
## FALSE  TRUE
##  0.62  0.38
```

We next look at a histogram of the summed of the natural niche Shapley value contributions. This is a continuous representation whether a subcatchment is inside or outside of the ecological niche. We see by how much subcatchments have negative or positive contributions of all natural niche variables to the overall environmental suitability prediction.

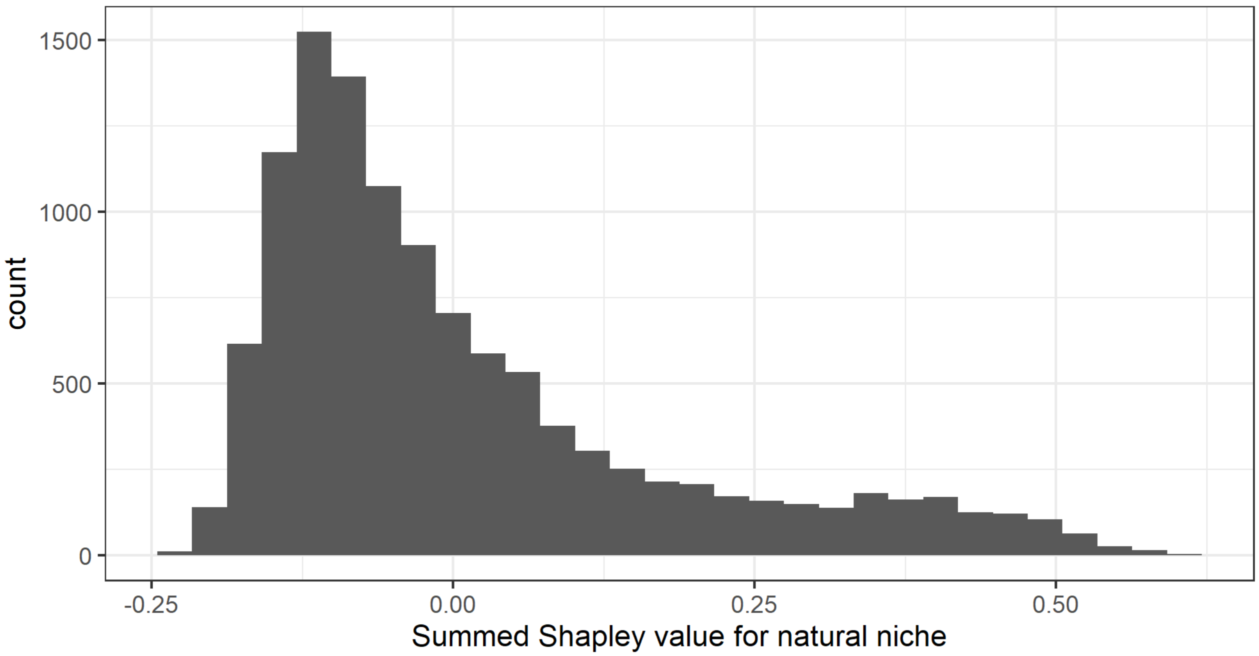

Figure 12. Distribution of summed Shapley value contributons to natural niche factors

The above summaries highlight, broadly, how natural niche factors contribute positively to environmental suitability predictions. Because each of the data points underlying these distributions is a geographic unit, we can therefore geographically map either binary areas or continuous areas where we may expected species to have suitable environments based on abiotic niche factors - the expected distribution. We can contrast this with the outputs of a traditional species distribution model which only shows the relative environmental suitability.

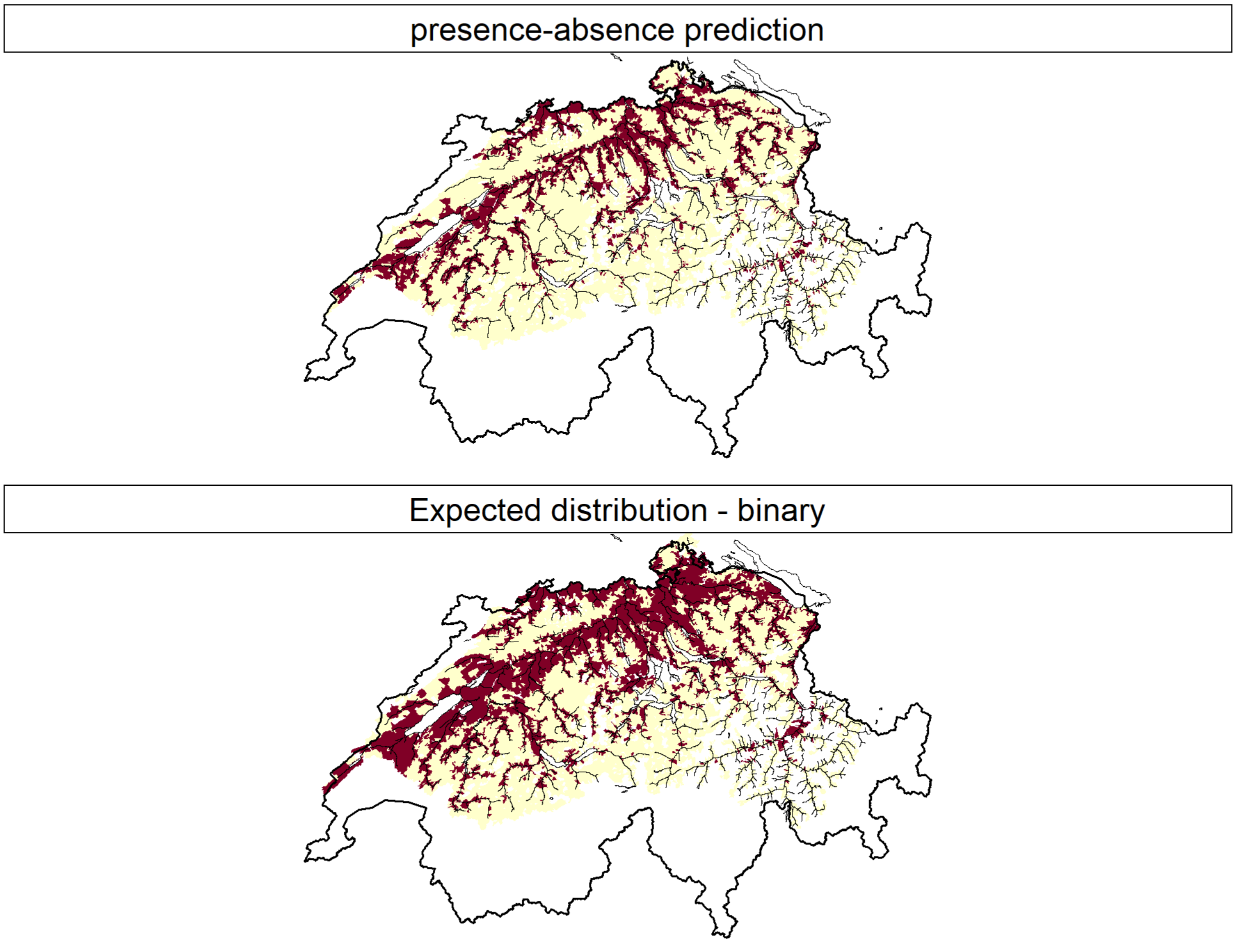

Figure 13. Comparison of predicted distribution of presence-absence from traditional species distribution model and the expected distribution quantified by Shapley values for natural niche factors

In the above plot, we see the contrast between the environmental suitability scores and species predicted presence (red) or absence (yellow) by thresholding these scores. These two plots take into account all variables effects at once on the environmental suitability and presence-absence prediction. In contrast, we define the binary expected distribution only by the natural niche variables.

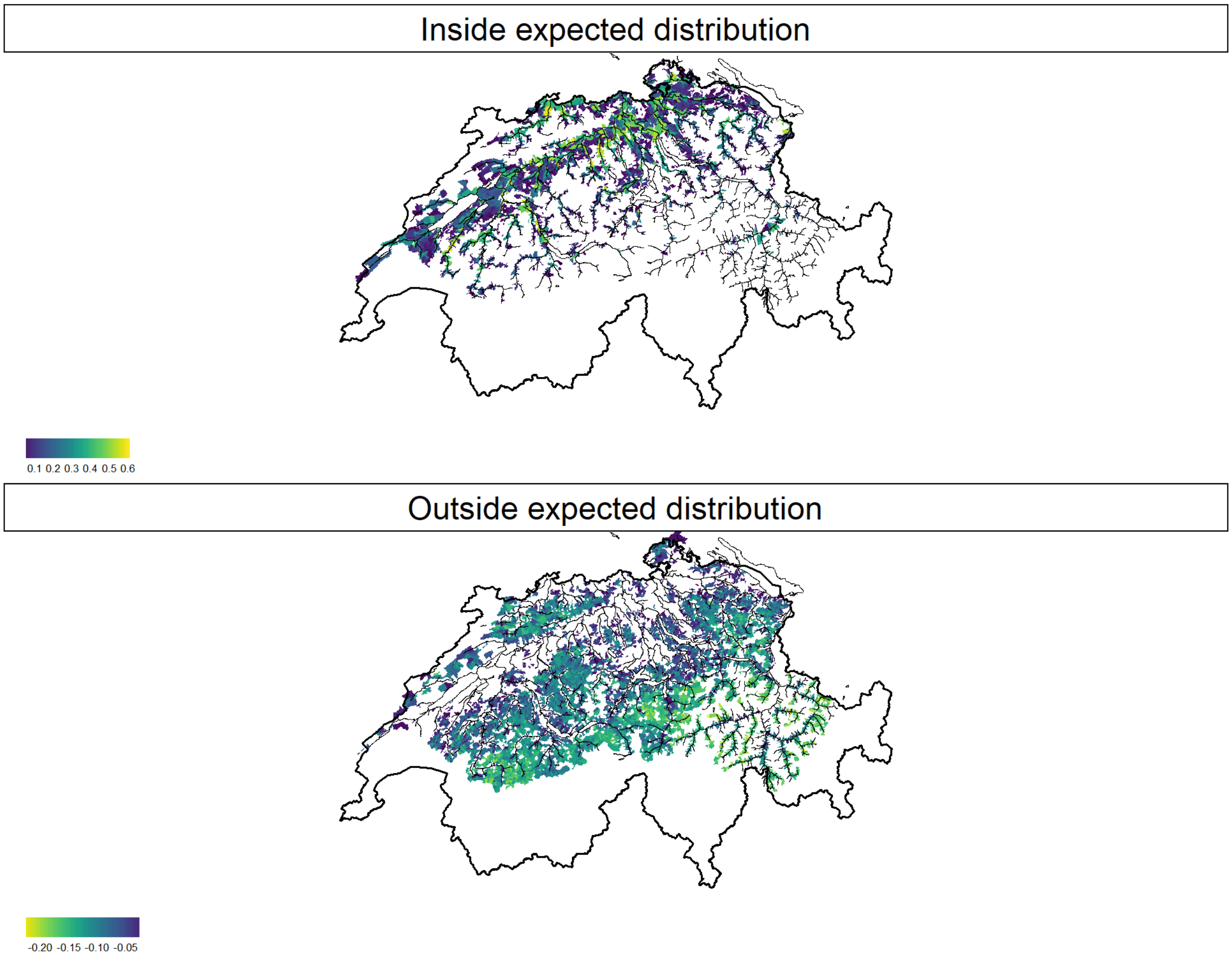

Figure 14. Comparison of areas inside and outside of binary expected distribution expressed as the environmental suitability scores inside and outside of the expected distribution.

The distributions in the above plot highlights the novelty of producing expected distributions, in addition to realized environmental suitability predictions, because we can now determine where a species should occur, but does not (i.e., the shadow distribution). Note that, fitting a species distribution model with only the niche related factors also does not solve this challenge. This is because the variables related to human factors still influence the species distribution. Therefore, simply ignoring these variables can confound the fitted model, the recovered response curves for the niche factors, and the resulting environmental suitability predictions. Instead, we fit the model including all the variables and then partition their individual effects at a local scale using the Shapley values to recover the regions where niche factors positively contribute to species distributions.

Now that we have defined the expected distribution, we can define the shadow distribution by expressing the effect of threats within the expected distribution.

4b. Binary shadow distribution We define the binary shadow distribution as the presence or absence of negative threat factor Shapley values within the expected distribution. As such, for a given sub-catchment, we first determine if the distribution first falls within expected distribution. If within the expected distribution, we determine within the catchment there is a negative Shapley value for a threat factor, indicating this threat negatively contributes to the environmental suitability prediction, despite a positive contribution of natural niche factors.

We determine two threat categories of "habitat-loss" threats and "connectivity" threats. The following variables were habitat-loss related threat factors in our framework: (low) floodplain cover, (low) wetlands cover, (high) river morphological modification index, and (high) urbanisation. We considered these together by average the net effect of habitat threats. We considered the connectivity alone and therefore take the raw Shapley value.

Define threat factors:

```
# define the variables that fall into each threat category
habitat_threat <- c("local_wet_SHAP", # wetland proportion cover
                    "local_flood_SHAP",  # floodplain proportion cover
                    "local_imd_log10_ele_residual_SHAP", # urbanisation proportion cover
                    "ecoF_eco_mean_ele_residual_SHAP") # river anthopic modification index

connectivity_threat <- c("local_asym_cl_log10_SHAP")
```

```
# add column to identify whether contribution of habitat
# variable to environmental suitability is positive or negative
shap_final$neg_habitat <-
  rowMeans(st_drop_geometry(shap_final[which(names(shap_final) %in% habitat_threat)])) < 0

# add column to identify whether contribution of connectivity
# variable to environmental suitability is positive or negative
shap_final$neg_con <-
  rowMeans(st_drop_geometry(shap_final[connectivity_threat])) < 0
```

Proportion of catchments inside niche with negative habitat effect (==T):

```
table(shap_final$neg_habitat) / sum(!is.na(shap_final$neg_habitat))
```

```
##
##     FALSE      TRUE
## 0.4757013 0.5242987
```

Proportion of catchments inside niche with negative connectivity effect (==T)

```
table(shap_final$neg_con) / sum(!is.na(shap_final$neg_con))
```

```
##
##     FALSE      TRUE
## 0.7393181 0.2606819
```

Binary shadow distribution: define threat categories We then used these contribution scores to demonstrate the areas of a species distributions that fall into the following categories:

1. outside of the expected distribution
2. inside the expected distribution - with no threats
3. inside the expected distribution - negative mean contribution of habitat variables
4. inside the expected distribution - negative mean contribution of connectivity variable
5. inside the expected distribution - negative mean contribution of habitat and connectivity threats

We would define the categories 3-5 as falling inside the shadow distribution of the species. We chose the above categories to help understand the major threats that lead to a location falling into the shadow distribution. For any given system, species, or threat landscape, these categories can be adapted.

```
# We used the following logical statements to develop this categorization - which of course should be a
  shap_final$niche_categories <- as.factor(

    ifelse(shap_final$natural_niche == T &
             shap_final$neg_con == F &
             shap_final$neg_habitat == F,

           "2. inside expected distribution + no threat",

           ifelse(shap_final$natural_niche == T &
                    shap_final$neg_con == T &
                    shap_final$neg_habitat == F,

                  "3. shadow - poor connectivity (C)",

                  ifelse(shap_final$natural_niche == T &
                           shap_final$neg_con == F &
                           shap_final$neg_habitat == T,

                         "4. shadow - poor habitat (H)",

                         ifelse(shap_final$natural_niche == T &
                                  shap_final$neg_con == T &
                                  shap_final$neg_habitat == T,

                                "5. shadow - poor C + H",

                                ifelse(shap_final$natural_niche == F,
                                       "1. outside ecological niche",
                                       NA))))))
```

We can investigate the proportion of the landscape that falls into each of the above categories:

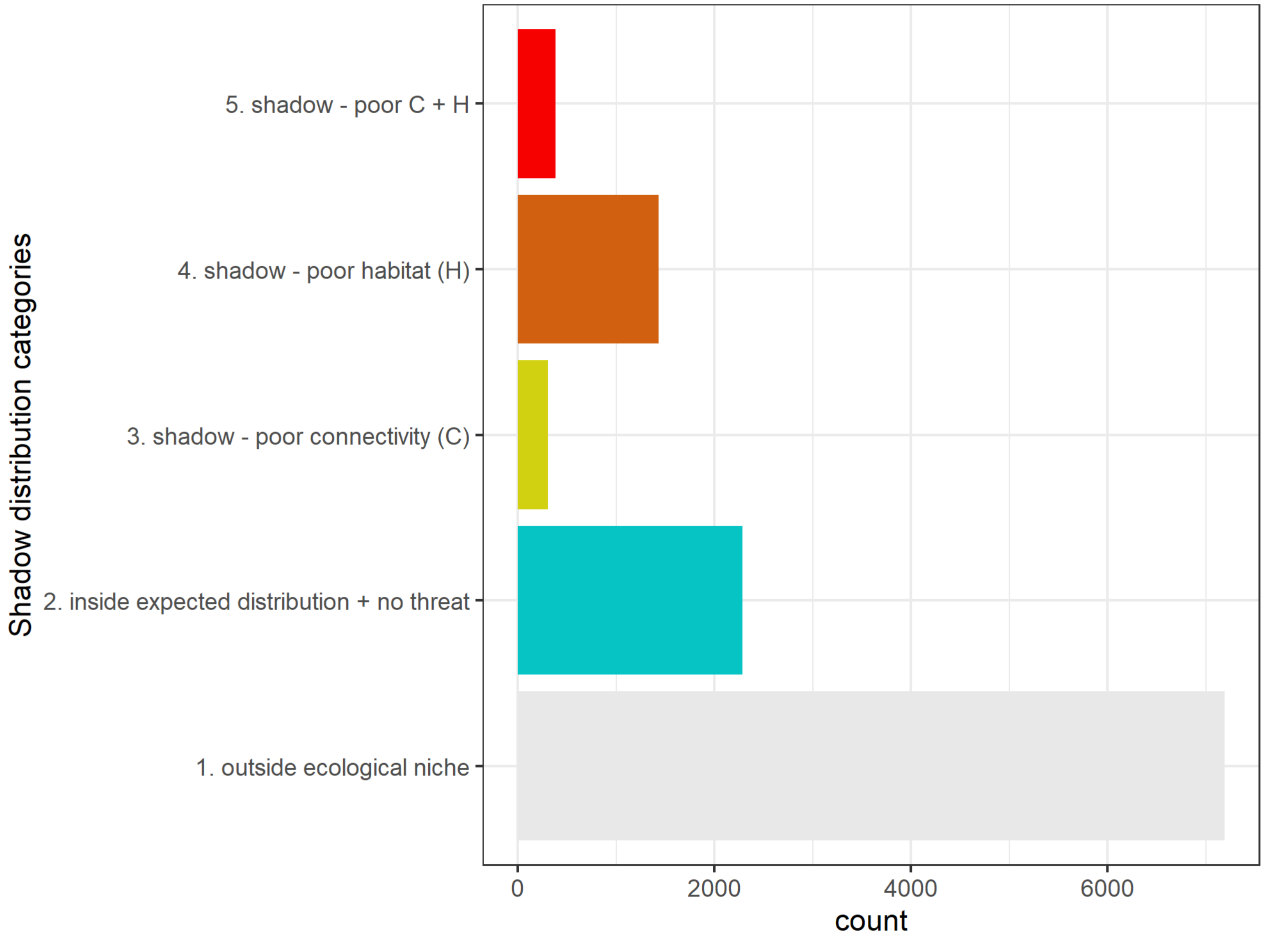

Figure 15. Relative proportion of subcatchments falling into different categories of the expected and shadow distribution

In the above plot we see relative proportions of each distribution category. Categories 1-2 represent inside or outside of the expected distribution, whereas categories 3-5 indicate the shadow distribution inside the expected distribution. We can also spatially map these categories to see the overall spatial distribution of different threats and their combinations, and also regions where threats are expected to have a limited effect on species distributions.

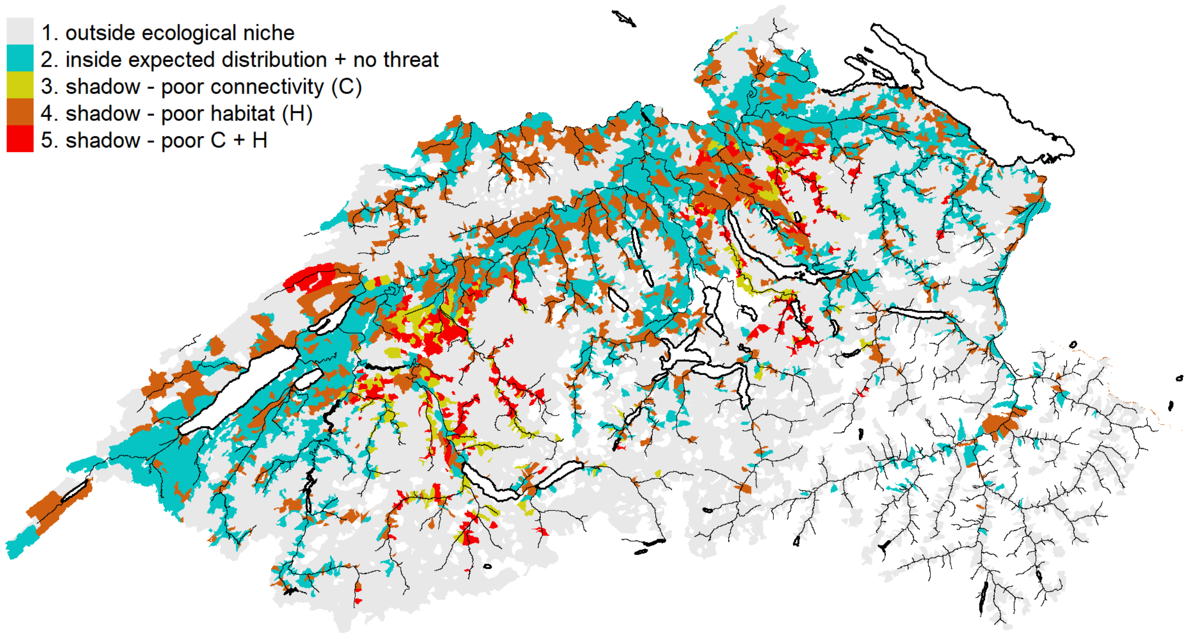

Figure 16. Map of expected and shadow distribution types across subcatchments

We interpret the above distribution as representing the shadow distribution of the species in this qualitative way. The areas coloured red, green or brown indicate the shadow distribution of the species. The areas in blue indicate the expected distribution that is not in the shadow distribution, and the areas in gray are outside of the expected distribution. In this way, we can visualise and summarise the spatial distribution of negative threat effects o n s pecies. W e e xpect s uch w orkflows co uld pr ovide im portant fe ature inputs to systematic conservation planning exercises such as features to zonation, marxan or prioritizr, especially where threats are to be alleviated as highlighted in (30).

4c. Shadow distribution - quantitative While the summary of areas inside or outside of the expected distributions and shadow distribution is helpful as a broad overview of areas potentially influence by threats, it is also important to demonstrate the magnitude of reduction in environmental suitability due to threat factors. This can be achieved through summarising the **quantitative shadow distribution** of a species.

The quantitative shadow distribution estimates the loss of environmental suitability due to human threats in areas falling within the expected distribution. We estimate the quantitative shadow distribution by calculating the difference between environmental suitability scores of the expected distribution and the observed distribution. We express this as a proportional loss of environmental suitability in the observed distribution.

The observed distribution could simply be the model prediction (i.e., raw output) of the species distribution model, giving an indicator of environmental suitability. However, as a minor technicality, to maintain consistency in approaches we calculate the model prediction from the sum of all the Shapley values for all the variables in the model + a baseline prediction - which in theory gives the model prediction for an observation. Deviations can occur due to the bootstrapped nature of the Shapley value but we observed these to be very minor (r^2 between both approaches ~0.99).

The following steps are necessary to calculate the quantitative shadow distribution:

1. *Definition of a baseline value*
2. *Prediction of environmental suitability*
3. *Partition Shapley value contributions to threats vs. non-threats.*
4. *Decide on strategy to estimate quantitative expected distribution*
5. *Identify qualitative expected distribution*
6. *Calculate the difference between observed distribution and expected distribution - giving the quantitative shadow distribution*

Working through the code to calculate quantitative shadow distribution

1. *Definition of a baseline value.* We must define a baseline value as the average of the predicted values. This is the reference value that a Shapley value is added to (+ or -) to determine the contribution to the local prediction of a given variable.

```
### ----
# step 1: Definition of baseline value
baseline_value <- mean(shap_final$suitability, na.rm = T)
```

2. *Prediction of environmental suitability.* Here we sum the Shapley values across all variables which calculates the prediction from the model (environmental suitability) for a given observation (sub-catchment).

```
### ----
# step 2: Prediction of environmental suitability
# we calculate environmental suitability from sum of Shapley values
shap_final$shap_all_sum <- rowSums(
  st_drop_geometry(
    shap_final[,names(shap_final) %in%
                 c(natural_niche_factors,
                   connectivity_threat,
                   habitat_threat)]
    ),
  na.rm = T)

# The environmental suitability is the shapley sum + the baseline value.
shap_final$shap_suit_baseline <- shap_final$shap_all_sum + baseline_value
# Here note that values < 0 or > 1 can occur in the Shapley
# version of environmental suitability due to the bootstrap
# nature of the Shapley values. As you approach infinity
# shapley value runs the simulated environmental suitability
# approaches the predicted environmental suitability of the
# model. We found no important changes in the shapley values
# between 1000 runs and 10,000 runs that we used in our final analysis.
```

3. *Partition Shapley value contributions to threats vs. non-threats.* We sum the Shapley values for threats and non-threats separately, to obtain the independent contributions to environmental suitability scores for different types of environmental variables.

```
### ----
# Step 3. Partition shapley value contributions to threats vs. non-threats

# Define threat factors
threat_factors <- c(connectivity_threat,
                    habitat_threat)

# Create matrix of only threat columns
threat_shaps <- st_drop_geometry(shap_final[,names(shap_final) %in%
                                              c(threat_factors)])
threat_shaps[threat_shaps<0] <- NA
# remove areas where threat level benefit species.
# This occurs in unthreatened locations where threats area already alleviated.
str(threat_shaps, 1)
```

```
## 'data.frame':    15130 obs. of  4 variables:
##  $ local_asym_cl_log10_SHAP         : num  0.1439 0.0544 NA 0.1018 0.1099 ...
##  $ ecoF_eco_mean_ele_residual_SHAP  : num  0.0179 0.04846 NA 0.01739 0.00023 ...
##  $ local_imd_log10_ele_residual_SHAP: num  0.00283 0.08812 NA 0.14517 NA ...
##  $ local_flood_SHAP                 : num  NA 0.0449 NA NA NA ...
```

```
# We calculated natural niche before
shap_final$nn_sum_mask <- ifelse(shap_final$natural_niche < 0,
                                 NA,
                                 shap_final$natural_niche)
```

4. *Decide on strategy to estimate quantitative expected distribution.* Depending on the users needs, there are different ways to compare the quantitative expected distribution to the observed distribution. These approaches vary in their assumption about the alleviation of threats to move from the observed environmental suitability (with threat effects) to the expected environmental suitability (with threat effects removed). We expect the most accurate representation of the expected suitability is to calculate expected suitability when threats are removed completely, i.e., simulating a baseline or reference state of the subcatchment. To simulate this alleviation of threats, we followed three strategies that converted Shapley values for threats to positive values:

   - Converting negative threat Shapley values to a maximum positive Shapley value for that threat (a best case-scenario). For caution, and to avoid spuriously large positive Shapley values in unusual sites, we used the 95th quantile of Shapley values per threat as our correction as our measure of the maximum.
   - Converting negative Shapley values to 0 which indicates if threats no longer have a negative contribution to environmental suitability but also do not support environmental suitability (very conservative scenario, assuming little impact of threat allevation).
   - Converting negative Shapley values to the mean positive Shapley values (conservative baseline scenario, assuming threat alleviation is inefficient).

These scenarios help quantify uncertainty the change in environmental suitability when "alleviating" threats under different assumptions.

The definition of the expected suitability inside the expected distribution is the key step in the definition of the shadow distribution. This step sums the natural niche Shapley values and the baseline prediction of the model with the **corrected** or **alleviated** threat values. In this way, we simulate the removal of the threat from the model prediction, which, if threats act negatively, then increases the predicted environmental suitability.

5. *Identify binary expected distribution.* We next remove all areas from consideration that are outside of the expected distribution (defined by positive summed natural niche Shapley values), and therefore inside the ecological niche, of the species. This step is important to ensure that only the areas where we expect a species to naturally occur are included in the calculation of the species shadow distribution.

```
### ----
# Step 4 Estimate quantitative expected distribution
# Step 5 Identify qualitative niches (through natural_niche column)

# Calculate for the threat shaps
corrected_threat_shaps <- rowSums(apply(threat_shaps, 2, function(x){
      x[is.na(x)] <- quantile(x, 0.95, na.rm = T)
      return(x)}))
```

```r
# Add the alleviated effects of the threats back to the observed distributions
shap_final$expected_distribution <- shap_final$natural_niche_value +
  corrected_threat_shaps +
  baseline_value

# Mask the expected suitability by the qualitative definition
# as inside or outside of the expected distribution / natural niche of the species
shap_final$expected_distribution <- ifelse(shap_final$natural_niche_value < 0,
                                           NA,
                                           shap_final$expected_distribution)

# remove small number of shapley values which due to stochastic calculation generate
# values > 1
shap_final$expected_distribution[shap_final$expected_distribution > 1] <- 1
```

6. *Calculate the difference between observed distribution and expected distribution - giving the quantitative shadow distribution.* This estimates the environmental suitability loss due to human threats that fall within the natural niche of the species.

```r
### ---
# Step 6 Calculate the quantitative shadow distribution

# We must first define the observed distribution, which we
# do as the predicted environmental suitability inside the
# natural niche of the species. Above we already . Remember,
# that this prediction has both the effects of the natural
# niche factors and the threat factors within the estimate
# of the environmental suitability.
shap_final$observed_distribution <- ifelse(is.na(shap_final$nn_sum_mask),
                                           NA,
                                           shap_final$shap_suit_baseline)

# Here, we expect the most important summary of the shadow
# distribution is the percentage loss of environmental suitability
# as a result of threats.
shap_final$SD_OratioE <- shap_final$observed_distribution /
  shap_final$expected_distribution

# However, we can also calculate the raw different between
# expected and observed distributions.
shap_final$SD_OminusE <- shap_final$observed_distribution -
  shap_final$expected_distribution

# And, the difference as a percentage of the expected distribution.
shap_final$SD_OpercentOfE <- (shap_final$SD_OminusE) /
  shap_final$expected_distribution
```

Next we can spatially map the shadow distribution of the species.

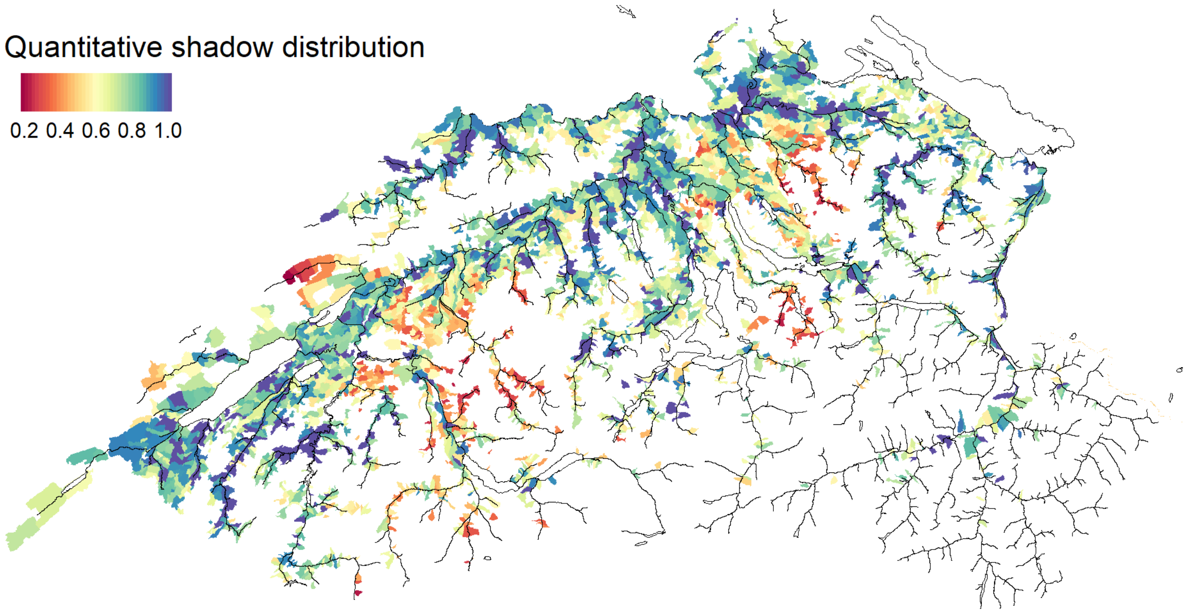

Figure 17. Species quantitative shadow distribution expressed as the ratio of the observed environmental suitability to the expected environmental suitability

```r
print(sessionInfo())
```

```
## R version 4.1.2 (2021-11-01)
## Platform: x86_64-w64-mingw32/x64 (64-bit)
## Running under: Windows 10 x64 (build 19045)
##
## Matrix products: default
##
## locale:
## [1] LC_COLLATE=English_United Kingdom.1252
## [2] LC_CTYPE=English_United Kingdom.1252
## [3] LC_MONETARY=English_United Kingdom.1252
## [4] LC_NUMERIC=C
## [5] LC_TIME=English_United Kingdom.1252
##
## attached base packages:
## [1] stats     graphics  grDevices utils     datasets  methods   base
##
## other attached packages:
##  [1] kernelshap_0.3.8     fastshap_0.0.7       pROC_1.18.0
##  [4] Boruta_8.0.0         randomForest_4.7-1.1 sf_1.0-9
##  [7] tmap_3.3-3           terra_1.7-3          lubridate_1.9.2
## [10] forcats_1.0.0        stringr_1.5.0        dplyr_1.0.10
## [13] purrr_1.0.1          readr_2.1.4          tidyr_1.3.0
## [16] tibble_3.1.8         ggplot2_3.4.2        tidyverse_2.0.0
## [19] pacman_0.5.1
##
## loaded via a namespace (and not attached):
##   [1] leafem_0.2.0          colorspace_2.0-3      deldir_1.0-6
##   [4] class_7.3-19          mclust_6.0.0          leaflet_2.1.1
##   [7] htmlTable_2.4.1       base64enc_0.1-3       dichromat_2.0-0.1
##  [10] rstudioapi_0.15.0     proxy_0.4-27          farver_2.1.1
##  [13] earth_5.3.1           mvtnorm_1.1-3         fansi_1.0.3
##  [16] ranger_0.14.1         codetools_0.2-18      splines_4.1.2
##  [19] cachem_1.0.7          knitr_1.43            ade4_1.7-20
##  [22] Formula_1.2-4         jsonlite_1.8.4        mda_0.5-3
##  [25] tmaptools_3.1-1       cluster_2.1.2         png_0.1-8
##  [28] compiler_4.1.2        backports_1.4.1       assertthat_0.2.1
##  [31] Matrix_1.5-3          fastmap_1.1.0         cli_3.5.0
##  [34] htmltools_0.5.4       tools_4.1.2           gtable_0.3.3
##  [37] glue_1.6.2            reshape2_1.4.4        Rcpp_1.0.9
##  [40] PresenceAbsence_1.1.11 jquerylib_0.1.4      raster_3.6-14
##  [43] vctrs_0.6.1           ape_5.6-2             nlme_3.1-153
##  [46] iterators_1.0.14      leafsync_0.1.0        crosstalk_1.2.0
##  [49] lwgeom_0.2-10         xfun_0.39             maxnet_0.1.4
##  [52] timechange_0.1.1      lifecycle_1.0.4       gtools_3.9.4
##  [55] ecospat_3.4           XML_3.99-0.13         MASS_7.3-58.1
##  [58] scales_1.2.1          hms_1.1.3             parallel_4.1.2
##  [61] RColorBrewer_1.1-3    yaml_2.3.7            gridExtra_2.3
##  [64] TeachingDemos_2.12    sass_0.4.5            rpart_4.1-15
##  [67] latticeExtra_0.6-30   reshape_0.8.9         stringi_1.7.8
##  [70] foreach_1.5.2         plotrix_3.8-2         checkmate_2.1.0
##  [73] permute_0.9-7         e1071_1.7-12          poibin_1.5
```

```
##  [76] rlang_1.1.1         pkgconfig_2.0.3       pracma_2.4.2
##  [79] evaluate_0.21       lattice_0.20-45       labeling_0.4.2
##  [82] ks_1.14.0           htmlwidgets_1.6.1     tidyselect_1.2.0
##  [85] gbm_2.1.8.1         biomod2_4.2-1         plyr_1.8.8
##  [88] magrittr_2.0.3      R6_2.5.1              Hmisc_4.7-2
##  [91] generics_0.1.3      DBI_1.1.3             foreign_0.8-81
##  [94] mgcv_1.9-0          pillar_1.9.0          withr_2.5.2
##  [97] units_0.8-1         stars_0.6-0           survival_3.2-13
## [100] abind_1.4-5 ##      sp_1.5-1              nnet_7.3-16
[103] interp_1.1-3 ##     KernSmooth_2.23-20    utf8_1.2.2
[106] tzdb_0.3.0 ##       rmarkdown_2.23        nabor_0.5.0
[109] jpeg_0.1-10 ##      grid_4.1.2            data.table_1.14.6
[112] vegan_2.6-4 ##      plotmo_3.6.2          digest_0.6.31
[115] classInt_0.4-8 ##   munsell_0.5.0         viridisLite_0.4.2
[118] bslib_0.5.0
```

Supplementary References

1. Araújo, M. B. *et al.* Standards for distribution models in biodiversity assessments. *Sci. Adv.* **5**, eaat4858 (2019).
2. Zurell, D. *et al.* A standard protocol for reporting species distribution models. *Ecography* **43**, 1261–1277 (2020).
3. Guillera-Arroita, G. *et al.* Is my species distribution model fit for purpose? Matching data and models to applications. *Glob. Ecol. Biogeogr.* **24**, 276–292 (2015).
4. Sillero, N. & Barbosa, A. M. Common mistakes in ecological niche models. *Int. J. Geogr. Inf. Sci.* **35**, 213–226 (2021).
5. Valavi, R., Elith, J., Lahoz-Monfort, J. J. & Guillera-Arroita, G. Modelling species presence-only data with random forests. *Ecography* **44**, 1731–1742 (2021).
6. Valavi, R., Guillera-Arroita, G., Lahoz-Monfort, J. J. & Elith, J. Predictive performance of presence-only species distribution models: a benchmark study with reproducible code. *Ecol. Monogr.* **92**, (2022).
7. Brodersen, J., Hellmann, J. & Seehausen, O. Erhebung der Fischbiodiversität in Schweizer Fliessgewässern. Progetto Fiumi Schlussbericht. (2023) doi:10.55408/eawag:30020.
8. Alexander, T. & Seehausen, O. *Diversity, Distribution and Community Composition of Fish in Perialpine Lakes. "Projet Lac" Synthesis Report*. (2021). doi:10.55408/eawag:24051.
9. Vonlanthen, P. *et al.* Genetic analysis of potential postglacial watershed crossings in Central Europe by the bullhead (Cottus gobio L.). *Mol. Ecol.* **16**, 4572–4584 (2007).
10. Lucek, K., Keller, I., Nolte, A. W. & Seehausen, O. Distinct colonization waves underlie the diversification of the freshwater sculpin (Cottus gobio) in the Central European Alpine region. *J. Evol. Biol.* **31**, 1254–1267 (2018).
11. Valavi, R., Elith, J., Lahoz-Monfort, J. J. & Guillera-Arroita, G. blockCV: An r package for generating spatially or environmentally separated folds for k-fold cross-validation of species distribution models. *Methods Ecol. Evol.* **10**, 225–232 (2019).
12. Peterson, E. & Hoef, J. V. STARS: An ArcGIS Toolset Used to Calculate the Spatial Information Needed to Fit Spatial Statistical Models to Stream Network Data. *J. Stat. Softw.* **56**, 1–17 (2014).
13. Kattwinkel, M., Szöcs, E., Peterson, E. & Schäfer, R. B. Preparing GIS data for analysis of stream monitoring data: The R package openSTARS. *PLOS ONE* **15**, e0239237 (2020).
14. Wood, S. Fast stable restricted maximum likelihood and marginal likelihood estimation of semiparametric generalized linear models. *J. R. Stat. Soc. B* **73**, 3–36 (2011).
15. Kursa, M. B. & Rudnicki, W. R. Feature Selection with the Boruta Package. *J. Stat. Softw.* **36**, 1–13 (2010).
16. Arif, S. & MacNeil, M. A. Predictive models aren't for causal inference. *Ecol. Lett.* **25**, 1741–1745 (2022).
17. Morrissey, M. B. ; R. Multiple Regression Is Not Multiple Regressions: The Meaning of Multiple Regression and the Non-Problem of Collinearity. *Philos. Theory Pract. Biol.* **10**, (2018).
18. Thuiller, W. *et al.* biomod2: Ensemble Platform for Species Distribution Modeling. (2023).
19. Lucas, T. C. D. A translucent box: interpretable machine learning in ecology. *Ecol. Monogr.* **90**, e01422 (2020).
20. Ryo, M. *et al.* Explainable artificial intelligence enhances the ecological interpretability of black-box species distribution models. *Ecography* **44**, 199–205 (2021).
21. Wadoux, A. M. J.-C. & Molnar, C. Beyond prediction: methods for interpreting complex models of soil variation. *Geoderma* **422**, 115953 (2022).
22. Shapley, L. S. A value for n-person games. in *Contributions to the Theory of Games* vol. II 31–40 (Princeton University Press, Princeton, 1953).
23. Štrumbelj, E. & Kononenko, I. Explaining prediction models and individual predictions with feature contributions. *Knowl. Inf. Syst.* **41**, 647–665 (2014).
24. Strumbelj, E. & Kononenko, I. An Efficient Explanation of Individual Classifications using Game Theory. *J. Mach. Learn. Res.* **11**, 1–18 (2010).

25. *xxAI - Beyond Explainable AI: International Workshop, Held in Conjunction with ICML 2020, July 18, 2020, Vienna, Austria, Revised and Extended Papers*. vol. 13200 (Springer International Publishing, Cham, 2022).
26. Molnar, C. *Interpretable Machine Learning: A Guide for Making Black Box Models Interpretable*. (2022).
27. Lundberg, S. M. *et al.* Explainable machine-learning predictions for the prevention of hypoxaemia during surgery. *Nat. Biomed. Eng.* **2**, 749–760 (2018).
28. Greenwell, B. fastshap: Fast Approximate Shapley Values. (2021).
29. Lundberg, S. M. & Lee, S.-I. A Unified Approach to Interpreting Model Predictions. in *Advances in Neural Information Processing Systems* vol. 30 (Curran Associates, Inc., 2017).
30. Salgado-Rojas, J., Hermoso, V. & Álvarez-Miranda, E. prioriactions: Multi-action management planning in R. *Methods Ecol. Evol.* (2023) doi:10.1111/2041-210X.14220.
