# Supplementary material for: Deconstructing the geography of human impacts on species’ natural distribution
Source: Nat Commun. 2024 Oct 14;15:8852. doi: 10.1038/s41467-024-52993-0 (PMC11473693; doi:10.1038/s41467-024-52993-0)
Supplement: Supplementary file 3 — Reporting Summary [file 41467_2024_52993_MOESM3_ESM.pdf]

nature portfolio

Corresponding author(s): Conor Waldock

Last updated by author(s): Jun 11, 2024

Reporting Summary

Nature Portfolio wishes to improve the reproducibility of the work that we publish. This form provides structure for consistency and transparency in reporting. For further information on Nature Portfolio policies, see our Editorial Policies and the Editorial Policy Checklist.

Statistics

For all statistical analyses, confirm that the following items are present in the figure legend, table legend, main text, or Methods section.

n/a	Confirmed	
☐	☒	The exact sample size (*n*) for each experimental group/condition, given as a discrete number and unit of measurement
☐	☒	A statement on whether measurements were taken from distinct samples or whether the same sample was measured repeatedly
☐	☒	The statistical test(s) used AND whether they are one- or two-sided<br>*Only common tests should be described solely by name; describe more complex techniques in the Methods section.*
☐	☒	A description of all covariates tested
☐	☒	A description of any assumptions or corrections, such as tests of normality and adjustment for multiple comparisons
☐	☒	A full description of the statistical parameters including central tendency (e.g. means) or other basic estimates (e.g. regression coefficient) AND variation (e.g. standard deviation) or associated estimates of uncertainty (e.g. confidence intervals)
☐	☒	For null hypothesis testing, the test statistic (e.g. *F*, *t*, *r*) with confidence intervals, effect sizes, degrees of freedom and *P* value noted<br>*Give P values as exact values whenever suitable.*
☐	☒	For Bayesian analysis, information on the choice of priors and Markov chain Monte Carlo settings
☐	☒	For hierarchical and complex designs, identification of the appropriate level for tests and full reporting of outcomes
☐	☒	Estimates of effect sizes (e.g. Cohen's *d*, Pearson's *r*), indicating how they were calculated

Our web collection on statistics for biologists contains articles on many of the points above.

Software and code

Policy information about availability of computer code

Data collection	No data were collected using computer code.
Data analysis	The code associated with reproducing the analysis and figures in this manuscript are provided here, https://doi.org/10.5281/zenodo.1362664988, with the full code pipeline to fit SDMs also available at https://doi.org/10.6084/m9.figshare.2478722787.<br><br>Random forests were fit using the R package randomForest (version 4.7-1.1). Generalized additive models were fitted using the R package 'mgcv' (version 1.8-38). To select variables in randomForests we used the BORUTA algorithm in the R package 'BORUTA' (version 7.0.0). For spatial cross validations we used the R package 'blockCV' (version 3.1-4). We calculate SHAP values using the R package 'fastshap' (version 0.1.1).

For manuscripts utilizing custom algorithms or software that are central to the research but not yet described in published literature, software must be made available to editors and reviewers. We strongly encourage code deposition in a community repository (e.g. GitHub). See the Nature Portfolio guidelines for submitting code & software for further information.

Data

Policy information about availability of data

All manuscripts must include a data availability statement. This statement should provide the following information, where applicable:

- Accession codes, unique identifiers, or web links for publicly available datasets
- A description of any restrictions on data availability
- For clinical datasets or third party data, please ensure that the statement adheres to our policy

The raw and processed data are available at https://doi.org/10.6084/m9.figshare.2478722787.

Research involving human participants, their data, or biological material

Policy information about studies with human participants or human data. See also policy information about sex, gender (identity/presentation), and sexual orientation and race, ethnicity and racism.

Reporting on sex and gender	NA
Reporting on race, ethnicity, or other socially relevant groupings	NA
Population characteristics	NA
Recruitment	NA
Ethics oversight	NA

Note that full information on the approval of the study protocol must also be provided in the manuscript.

Field-specific reporting

Please select the one below that is the best fit for your research. If you are not sure, read the appropriate sections before making your selection.

☐ Life sciences ☐ Behavioural & social sciences ☒ Ecological, evolutionary & environmental sciences

For a reference copy of the document with all sections, see nature.com/documents/nr-reporting-summary-flat.pdf

Ecological, evolutionary & environmental sciences study design

All studies must disclose on these points even when the disclosure is negative.

Study description	We used a species distribution modelling approach to model the environmental suitability across the spatial distribution of nine fish species in Switzerland using 11 environmental variables. We next applied model agnostic explainable artificial intelligence tools to these models.
Research sample	Presence-absence records came from quantitative and semi-quantitative field surveys conducted by scientific researchers, cantonal and national monitoring agencies, ecological consultancies.<br>• Project fieldwork data (CW, DJ, BW, BC, OS): Data were collected in the context of the University of Bern, Federal Office for the Environmental, Kanton Bern and Wyss Academy for Nature LANAT-3 project titled "Stopping the biodiversity loss of water bodies ¬- despite climate change". Data were collected under the Swiss animal experimentation licence (permit numbers 34546 BE11/2022 and 34150 BE95/2021).<br>• Projetto Fiumi: The main objective of Progetto Fiumi (Erhebung der Fischbiodiversität in Schweizer Fliessgewässern) was to survey fish biodiversity and its distribution in Swiss rivers and surveys were undertaken between 2013 and 2017.<br>• Module Stufen Konzept electrofishing data: Compiled by Dr. Pascal Vonlanthen and Dr. Sébastien Lauper in the context of compiling data collected under the biomonitoring protocol 'Module Stufen Konzept' (https://modul-stufen-konzept.ch).<br>• Kanton Berne electrofishing data: The Canton of Bern has provided us with an extract from its fisheries database for the period 1994 to 2022.
Sampling strategy	Sampling was undertaken using semi- and quantitative sampling. Sample sizes for species distribution models are acceptable above >30 presence-only records which was exceeded in our survey design.
Data collection	• Project fieldwork data (DJ, BW, BC, CW, OS): Electrofishing campaign from August to October 2022. We stratified sampling of sites based on 9 classes combining three human impacts levels (low, medium, high) and three elevation strata (low, medium, high) based on equal sized strata. From these strata, we sampled 46 sites and attempted to balance site selection across strata. We fished 100 meter stretches of wadeable streams with electrofishing at 46 sites in Switzerland. All fish were identified in the field, as best as possible, to species level, measured, and weighed. The determination of species from difficult groups was later confirmed in the laboratory or on the basis of the photos. Approximately 10-15 individuals of all species (license permitting) were retained to form

long-term research collections at the Naturhistorisches Museum Bern.
• Projetto Fiumi: Sites were surveyed between 2013 and 2017. Site selection was intended to representatively survey the diversity of Swiss watercourses. Surveys were conducted in September and October each year. A stretch of approximately 100 meters was quantitatively fished when rivers were small. Waterbodies that were too wide, too deep, or too fast-flowing were fished qualitatively, taking into account all habitats as much as possible. Non-wadeable, large rivers were sampled by boat equipped with an anode rake using the strip fishing method. Captured fish were stocked separately by strip, or by passage in the case of quantitative surveys. All fish were identified in the field, as best as possible, to species level, measured, and weighed. The determination of species from difficult groups was later confirmed in the laboratory or on the basis of the photos.
• Module Stufen Konzept electrofishing data: These data were compiled if they conformed to the electro-fishing component of the 'Module Stufen Konzept' (modular stepwise procedure) providing methods for the analysis and assessment of surface waters in Switzerland (https://modul-stufen-konzept.ch). These methods are developed to ensure assessment of watercourse status is in accordance with water protection legislation and was developed in collaboration with the federal government, cantonal government, EAWAG and the VSA. All data provided in this compilation had at least two electrofishing 'runs' recording all fish species encountered with the aim to describe the present species community (personal communication Dr. Pascal Vonlanthen and Dr. Sebastien Lauper).
• Kanton Bern electrofishing data: This mainly contains data collected at all construction sites during this time-period as well as some data from long-term monitoring sites.

Timing and spatial scale

Sampling spans from 2010 to 2022 across the Aare-Rhein catchment inside Switzerland.

Data exclusions

We did not exclude data

Reproducibility

We did not conduct experiments so no measures were taken to ensure reproducibility of experiments. We provide code and reproducible examples to facilitate researchers reproducing the analytical components of our work.

Randomization

A fundamental aim of our work is providing interpretable (understanding inner workings) and explainable (understanding why a prediction is made) models. Multi-collinearity induces challenges in interpreting the independence of variable effects and interpretation of SHAP values 40. Through the below procedure our final variables were highly decoupled having a median absolute correlation of 0.05, a 95th quantile of 0.26 (Figure S3). We therefore limit the impact of multicollinearity in our modelling (see Appendix 1 for full details). We first checked bi-plots and Spearman's rank correlations between variables and identified potentially confounding factors that would lead to misinterpretation of focal variable effects. We found elevation, discharge, slope and distance to lakes were often strongly related to 8 variables (morphological modification, urbanisation, livestock, nitrogen, phosphorous, insecticide, cropland, tree cover). We then fitted GAMs to relate these variables with the potential confounders and used the residuals from GAMs in our random forests. We retained only residual morphological modification and residual urbanisation which had biologically realistic relations with environmental suitability. The interpretation of these processed variables is the relative value of the variable given the site's elevation, discharge, slope and distance to lake. GAMs were fitted using the R package 'mgcv' (version 1.8-38). From our final pre-selected set of variables, we then identified and used only those that were statistically supported using the BORUTA algorithm in the R package 'BORUTA' (version 7.0.0). This method was developed to provide a statistically valid approach to remove variables that do not sufficiently improve the fit of random forest models 41.

Blinding

Our work was not experimental and blinding was not required.

Did the study involve field work? ☒ Yes ☐ No

Field work, collection and transport

Field conditions

New data were generated during electrofishing campaign from August to October 2022 by CW, DJ, BW, and BBC. We stratified sampling of sites based on 9 classes combining three human impacts levels (low, medium, high) and three elevation strata (low, medium, high) based on equal sized strata. From these strata, we sampled 46 sites and attempted to balance site selection across strata. We fished 100 meter stretches of wadeable streams with electrofishing at 46 sites in Switzerland. All fish were identified in the field, as best as possible, to species level, measured, and weighed. The determination of species from difficult groups was later confirmed in the laboratory or on the basis of the photos. Approximately 10-15 individuals of all species (license permitting) were retained to form long-term research collections at the Naturhistorisches Museum Bern.

Location

46 sites in the Aare-Rhein drainage inside switzerland.
Latitudes and longitudes of all sites surveyed are provided below:
47.11744444 , 7.492861111
47.09091667 , 7.399777778
47.02019444 , 7.491083333
47.05822222 , 7.54025
47.113589 , 7.53096
47.10330556 , 7.546194444
47.15277778 , 7.538638889
47.16511111 , 7.555027778
47.08788889 , 7.576305556
47.032957 , 7.639281
47.19275 , 7.580194444
46.9625 , 8.387222222
46.87980556 , 8.376138889

47.21472222 , 8.425027778
46.82819444 , 6.677527778
46.57658333 , 7.913638889
46.69877778 , 8.232944444
46.54158333 , 7.659666667
46.70297222 , 7.608888889
46.99661111 , 7.439277778
46.93833333 , 6.674
46.96108333 , 6.841416667
46.84880556 , 7.825472222
46.82705556 , 7.39875
46.94041667 , 7.208777778
46.71644444 , 7.327972222
46.82669444 , 7.322416667
47.01975 , 7.727611111
46.90636111 , 7.007694444
47.33352778 , 8.519861111
47.17158333 , 8.664416667
47.23683333 , 8.836888889
47.17880556 , 9.029111111
47.20888889 , 9.083611111
47.09441667 , 9.337833333
46.77555556 , 7.122222222
46.78375 , 7.11825
46.99288889 , 8.097055556
47.25191667 , 7.893583333
47.37086111 , 8.173527778
47.29886111 , 8.398333333
47.17575 , 8.989416667
47.19794444 , 8.859027778
47.17763889 , 7.790833333
47.09113889 , 7.311333333
47.19880556 , 8.814472222

Access & import/export

Data were collected under the Swiss animal experimentation licence (permit numbers 34546 BE11/2022 and 34150 BE95/2021).

Disturbance

Physical disturbance to the local habitat (100m stretch of river) was limited to only walking through the river to perform electrofishing samples. We limited harm and stress to fish by replacing water frequently, aerating and oxygenating water, ensuring adequate water per individual, and limiting stress during handling by using MS-222 as an anaesthetic and humanly euthanizing individuals using an overdose of MS-222.

Reporting for specific materials, systems and methods

We require information from authors about some types of materials, experimental systems and methods used in many studies. Here, indicate whether each material, system or method listed is relevant to your study. If you are not sure if a list item applies to your research, read the appropriate section before selecting a response.

Materials & experimental systems

n/a	Involved in the study
x	Antibodies
x	Eukaryotic cell lines
x	Palaeontology and archaeology
	x Animals and other organisms
x	Clinical data
x	Dual use research of concern
x	Plants

Methods

n/a	Involved in the study
x	ChIP-seq
x	Flow cytometry
x	MRI-based neuroimaging

Animals and other research organisms

Policy information about studies involving animals; ARRIVE guidelines recommended for reporting animal research, and Sex and Gender in Research

Laboratory animals	No laboratory animals used in this survey
Wild animals	We capture fish using standard backpack electro fishing protocols. Fish were exposed to an electrical current for a short period of time to immobilize and capture. Fish were kept for under 2 hours if released. After capture all fish were anesthetized using MS222 before identification and measurement taking around <1 minute per individual. Fish were kept in well-oxygenated containers with cold water in the shade. 10-15 individuals of all species captured at the 46 locations were kept for further taxonomic and morphological research (beyond the scope of this manuscript). In such cases, fishes were euthanised by an overdose of MS222 using 100-200mg/l concentration.
Reporting on sex	No reporting on sex relevant to this study.
Field-collected samples	Data were collected under the Swiss animal experimentation licence (permit numbers 34546 BE11/2022 and 34150 BE95/2021).
Ethics oversight	Data were collected under the Swiss animal experimentation licence (permit numbers 34546 BE11/2022 and 34150 BE95/2021).

Note that full information on the approval of the study protocol must also be provided in the manuscript.

Plants

Seed stocks	NA
Novel plant genotypes	NA
Authentication	NA
